# Genome-Wide High-Resolution Mapping of UV-Induced Mitotic Recombination Events in *Saccharomyces cerevisiae*

**DOI:** 10.1371/journal.pgen.1003894

**Published:** 2013-10-31

**Authors:** Yi Yin, Thomas D. Petes

**Affiliations:** Department of Molecular Genetics and Microbiology and University Program in Genetics and Genomics, Duke University Medical Center, Durham, North Carolina, United States of America; CABIMER, Universidad de Sevilla, Spain

## Abstract

In the yeast *Saccharomyces cerevisiae* and most other eukaryotes, mitotic recombination is important for the repair of double-stranded DNA breaks (DSBs). Mitotic recombination between homologous chromosomes can result in loss of heterozygosity (LOH). In this study, LOH events induced by ultraviolet (UV) light are mapped throughout the genome to a resolution of about 1 kb using single-nucleotide polymorphism (SNP) microarrays. UV doses that have little effect on the viability of diploid cells stimulate crossovers more than 1000-fold in wild-type cells. In addition, UV stimulates recombination in G1-synchronized cells about 10-fold more efficiently than in G2-synchronized cells. Importantly, at high doses of UV, most conversion events reflect the repair of two sister chromatids that are broken at approximately the same position whereas at low doses, most conversion events reflect the repair of a single broken chromatid. Genome-wide mapping of about 380 unselected crossovers, break-induced replication (BIR) events, and gene conversions shows that UV-induced recombination events occur throughout the genome without pronounced hotspots, although the ribosomal RNA gene cluster has a significantly lower frequency of crossovers.

## Author Summary

Nearly every living organism has to cope with DNA damage caused by ultraviolet (UV) exposure from the sun. UV causes various types of DNA damage. Defects in the repair of these DNA lesions are associated with the human disease xeroderma pigmentosum, one symptom of which is predisposition to skin cancer. The DNA damage introduced by UV stimulates recombination and, in this study, we characterize the resulting recombination events at high resolution throughout the yeast genome. At high UV doses, we show that most recombination events reflect the repair of two sister chromatids broken at the same position, indicating that UV can cause double-stranded DNA breaks. At lower doses of UV, most events involve the repair of a single broken chromatid. Our mapping of events also demonstrates that certain regions of the yeast genome are relatively resistant to UV-induced recombination. Finally, we show that most UV-induced DNA lesions are repaired during the first cell cycle, and do not lead to recombination in subsequent cycles.

## Introduction

Recombination occurs in both meiotic and mitotic cells. In budding yeast, there are about 100 meiotic crossovers per cell [1]. Although mitotic recombination events in *S. cerevisiae* are about 10^5^-fold less frequent than meiotic exchanges [2], homologous recombination (HR) is important for the repair of double-stranded DNA breaks (DSBs) that occur spontaneously or that are induced by DNA damage. Yeast strains that lack HR grow more slowly than wild-type strains, and are sensitive to DNA damaging agents [3]. In HR events in diploid cells, the broken chromosome is repaired utilizing an intact sister chromatid or homolog as a template. Most organisms also have a pathway termed “non-homologous end-joining” (NHEJ) in which the broken ends are re-joined by a mechanism that does not require sequence homology. In diploid cells of *S. cerevisiae*, HR is much more important than NHEJ for repair of DNA damage [4]. We will first discuss pathways of HR, followed by a description of UV-induced DNA damage, and the recombinogenic effects of this damage.

**Figure 1:**
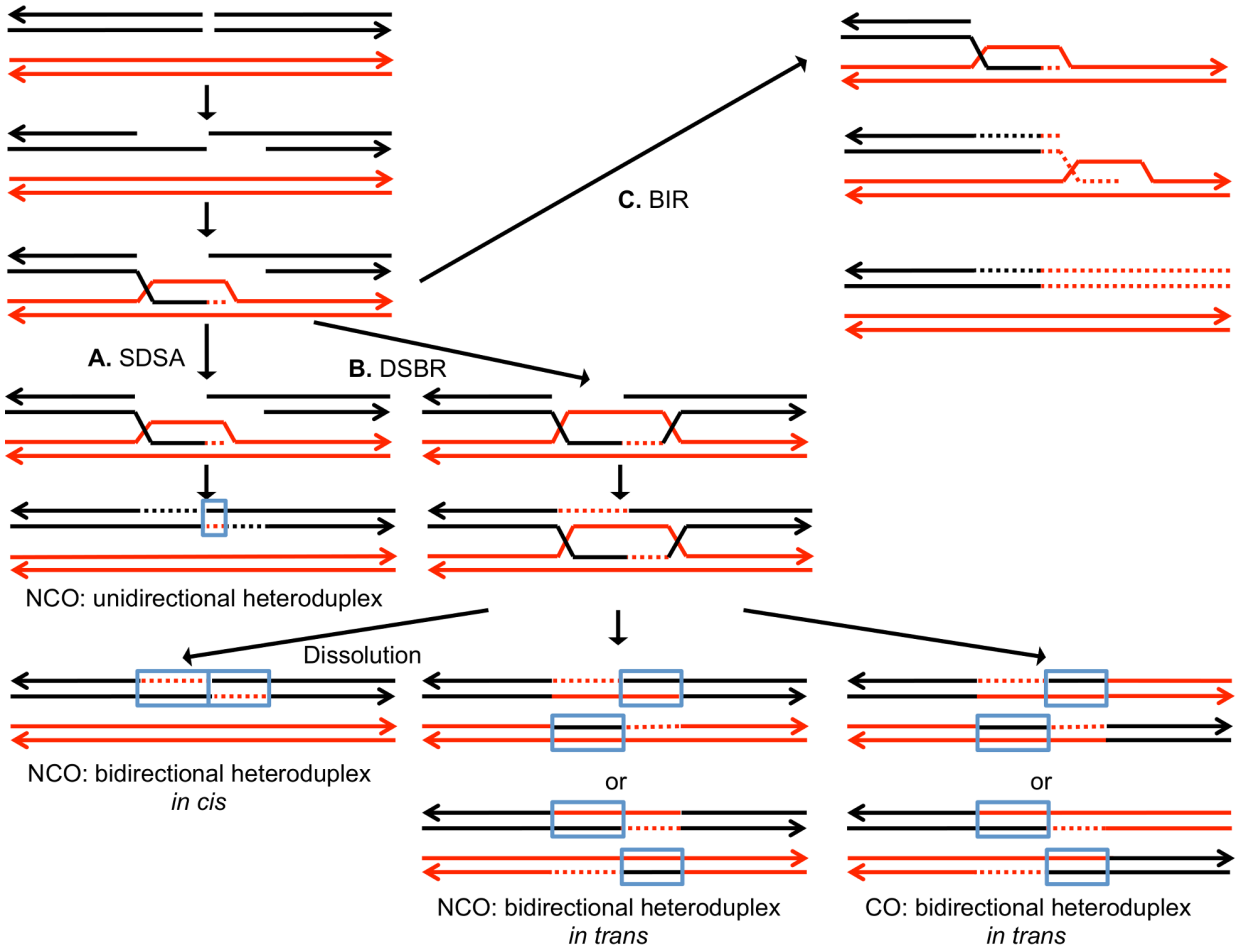
Mechanisms of homologous recombination. The two interacting DNA molecules are shown as double-stranded. The recombination event is initiated by a double-stranded DNA break (DSB) on the black molecule, and the broken ends are resected 5′ to 3′. In this depiction, the left processed end invades the red molecule, forming a heteroduplex. Dotted lines show DNA synthesis associated with the recombination event. A. Synthesis-dependent strand annealing (SDSA). The 3′ end of the invading strand is used as a primer for DNA synthesis, extending the D-loop. Dissociation of the D-loop, followed by re-annealing of the two broken ends results in a region of heteroduplex (outlined in blue) with flanking markers in the parental non-crossover (NCO) configuration. B. Double-strand break repair (DSBR). Following expansion of the D-loop, pairing occurs between the displaced strand and the right end of the broken chromosome, resulting in two regions of heteroduplex. The resulting double Holliday junction can be resolved by topoisomerase-mediated dissolution or by cleavage of the Holliday junctions to yield an NCO or a crossover (CO). C. Break-induced replication (BIR). In this pathway, the right broken chromosome is lost and the left molecule invades the homologous chromosome, resulting in duplication of sequences distal to the point of invasion.

DSBs can be repaired by a number of different HR pathways [5]. For all of these pathways, the broken DNA ends are processed by 5′ to 3′ degradation, followed by invasion of the processed chromosome end into either a sister chromatid or a homolog (Figure 1). In the synthesis-dependent strand annealing (SDSA) pathway, after strand invasion and DNA synthesis, the invading broken end is displaced and reanneals to the other broken end. The resulting product has a region of heteroduplex DNA and mismatches within the heteroduplex can be repaired to yield a gene conversion event unassociated with a crossover (Figure 1A). Alternatively, the broken ends can both engage in pairing with the intact chromosome resulting in a double Holliday junction (Figure 1B). This structure can be resolved to yield a crossover or non-crossover. As in the SDSA pathway, mismatches within the heteroduplex region can be repaired to generate a conversion event. Lastly, invasion of one broken end can result in the generation of a replication structure that duplicates sequences from the other chromosome from the point of invasion to the end of the chromosome (break-induced replication, BIR; Figure 1C).

**Figure 2:**
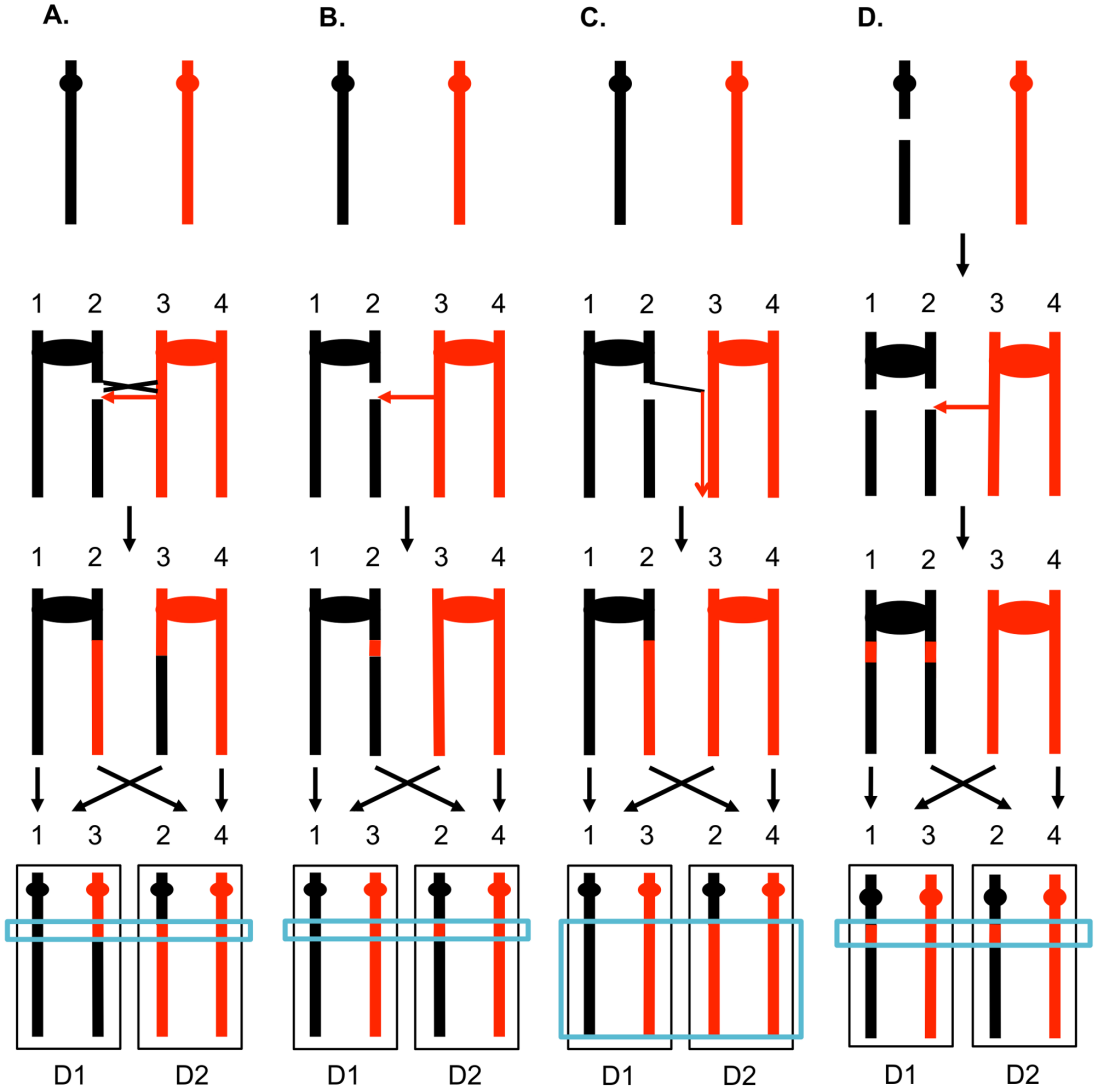
Patterns of mitotic gene conversions and crossovers. Chromosomes are shown as thick lines with the two homologs in red or black. Circles indicate the centromeres and blue boxes show gene conversions. A. Conversion and crossover associated with a DSB formed in S or G2 on one of the black chromatids. Since the broken chromosome acts as the recipient in a gene conversion event [60], sequence information derived from the red chromatid is non-reciprocally transferred in conjunction with the crossover. This type of conversion is termed “3∶1” since, at the site of the conversion, three of the four chromosomes of the two daughter cells (D1 and D2) have information derived from one of the homologs. If chromatids 1 and 3, and 2 and 4 co-segregate, any markers distal to the crossover will be homozygous. B. S or G2 conversion event unassociated with a crossover. If the DSB is repaired to generate a conversion unassociated with a crossover, a region of 3∶1 segregation will be observed, but the markers distal to the conversion event will remain heterozygous. C. Break-induced replication. The chromosome fragment centromere-distal to the DSB is lost, and the centromere-containing fragment invades and replicates the red chromosome to the telomere. D. Repair of a G1-induced DSB. A black chromosome with a G1-induced DSB is replicated to yield two sister-chromatids with breaks at the same positions. Gene conversion events are generated at the sites of repair, and the repair of one of the DSBs is associated with a crossover. Within the chromosomes of the two daughter cells, there is a region that is derived from only the red homolog (a “4∶0” conversion tract shown in the blue rectangle).

One consequence of mitotic recombination is to cause loss of heterozygosity (LOH) for markers near the initiating lesion (gene conversions) or extending distal from the initiating lesion to the end of the chromosome (crossovers and BIR events). In Figure 2, we show the repair of DSBs in diploid mitotic cells by HR involving the homolog. In Figure 2A, we show the repair of a single broken chromatid (G2 event) using the homolog as a template. The red and black colors indicate that the two homologs have single-nucleotide polymorphisms (SNPs) that allow the detection of recombination events. Figure 2A shows a crossover between chromatids 2 and 3. If chromatids 1 and 3 segregate into one daughter cell (D1), and 2 and 4 segregate into the other (D2), a reciprocal pattern of LOH would be observed. Segregation of unrecombined chromatids 1 and 4 into one cell and the recombined chromatids 2 and 3 into the other would not lead to LOH. These two patterns of segregation are equally frequent in yeast [6]. Our previous studies [2], [7] showed that most (80%) crossovers are associated with gene conversion events (indicated by boxes in Figure 2). In Figure 2B, we show a conversion event unassociated with a crossover which produces an interstitial LOH event in one of the daughter cells. The conversion events shown in Figure 2A and 2B are termed “3∶1” events since three of the chromatids have one type of SNP and one has the other within the boxed region. A BIR event produces a region of LOH that extends to the telomere in one but not both daughter cells (Figure 2C).

The 3∶1 conversion events shown in Figures 2A and 2B are expected from the repair of a single DSB generated in S or G2 of the cell cycle. In addition, since the chromosome with the DSB acts as a recipient of information derived from the intact chromosome, these conversion events have the pattern expected if the recombinogenic DSB was on the black chromosome [4]. We observed previously, however, that over half of the mitotic conversion events had a different form from that shown in Figures 2A and 2B. In Figure 2D, we show a conversion event unassociated with a crossover in which both daughter cells have an interstitial region of LOH that is homozygous for the same SNPs; these events are called “4∶0” conversions. We interpret 4∶0 events as resulting from the repair of two broken sister chromatids in which the DSBs are located at the same positions. One simple mechanism to obtain this pattern of breakage is that the recombinogenic DSB is generated in G1, the broken chromosome is replicated, and the two resulting broken chromatids are repaired in G2 (Figure 2D). The alternative model in which the DSB is generated and repaired in G1 is ruled out because such events would not be associated with LOH for markers located distal to the conversion event [8]. If the two broken chromatids are repaired to generate conversion tracts of the same lengths, a 4∶0 event is generated. If one conversion tract is longer than the other, repair of two broken sister chromatids can also generate hybrid 3∶1/4∶0 conversion tracts [2], [7]. Our previous studies indicated that most spontaneous crossovers had conversion events consistent with a G1-initiated DSB rather than a G2-initiated DSB [9], [10], and spontaneous events resembled those induced by gamma rays in G1-synchronized yeast cells [11].

UV results in DNA lesions that are both mutagenic and recombinogenic. The primary types of lesions caused by low dosages of UV-C (∼254 nm) are pyrimidine dimers including cyclobutane dimers (CPDs) and (6-4) photoproducts (6-4 PPs) [3]. Although CPDs can be reversed in yeast by the action of photolyase, the repair of most lesions in wild-type cells likely reflects nucleotide excision repair (NER). In NER, multiple proteins act to excise a short oligonucleotide containing the damaged bases. The resulting 30-nucleotide gap is filled in by DNA polymerase delta and/or epsilon [12], and the remaining nick is sealed by Lig1p. In yeast, as in many other organisms, UV-induced lesions are more quickly repaired in transcribed genes than in non-transcribed regions [3].

Although most UV-induced lesions are removed quickly by this error-free process, a small fraction of the 30-nucleotide gaps are expanded by the action of Exo1p, resulting in large RPA-coated gaps [13], [14]. These RPA-coated regions recruit Mec1p/Ddc2p and the 9-1-1 complex, followed by subsequent recruitment of other components of the DNA damage checkpoint [15]. In addition to checkpoints triggered by the action of Exo1p, if unrepaired lesions persist into the S-phase, single-stranded regions may also be generated during the re-start of blocked replication forks. Strong activation of Mec1p by UV is observed in S-phase cells, presumably by this mechanism [16].

Although it is clear from many previous studies that UV greatly elevates the frequency of mitotic recombination in yeast [17]–[23], the recombinogenic mechanism is not well understood. There are two types of models. First, it is possible that the recombinogenic lesion is generated by NER. Consistent with this model, Galli and Schiestl (1999) [20] observed that UV of G1-synchronized cells was not recombinogenic unless the cells were allowed to replicate. They concluded that the recombinogenic lesion was likely to represent an NER-associated gap that was replicated to produce the recombination-stimulating DSB. This model predicts that the gene conversion events associated with UV-treatment of G1-synchronized cells would be exclusively 3∶1 conversion events (Figure 2A). In a preliminary study [7], however, we found that about half of the observed UV-induced conversions were 3∶1 events and about half were 4∶0 events (Figure 2D). This observation is inconsistent with the simplest form of the model proposed by Galli and Schiestl.

An alternative model is that the unexcised dimers and other DNA lesions are the recombinogenic lesion. For example, replication forks stalled at an unexcised dimer may engage in replication re-start or be broken. Although both re-start and the repair of an S-phase DSB would be expected to involve an interaction with the sister chromatid [24], some fraction of these events could involve the homolog, resulting in LOH. Kadyk and Hartwell (1993) [21] showed that UV stimulates recombination between both sister-chromatids and homologs in NER-proficient cells. In *rad1/rad1* (NER-deficient) diploids, conversions, but not crossovers, were stimulated by UV in a replication-dependent manner [21]. One complication in interpreting this result is that Rad1p is involved with multiple recombination-related reactions [25]–[27] in addition to its role in NER. Regardless of this ambiguity, it is likely that unexcised dimers are recombinogenic. The summary of studies performed thus far is that some fraction of UV-induced recombination events reflects lesions resulting from NER and another fraction reflects unexcised dimers.

In the experiments described below, we examine mitotic crossovers and gene conversion events induced by UV in diploid cells. In G1-synchronized cells treated with high doses of UV, most of the events reflect the repair of two broken sister chromatids whereas at low doses, most events reflect repair of a single broken chromatid. We also show that UV induces crossovers more efficiently than BIR events. We mapped the distribution of about 100 UV-induced LOH events selected on chromosome V and about 400 unselected LOH events throughout the genome. We found that the unselected events were widely distributed throughout the genome with no very strong hotspots. The ribosomal RNA gene cluster, however, was significantly “cold” for crossovers compared to the rest of the genome.

## Results

### Detection and mapping of mitotic crossovers and gene conversions

**Figure 3:**
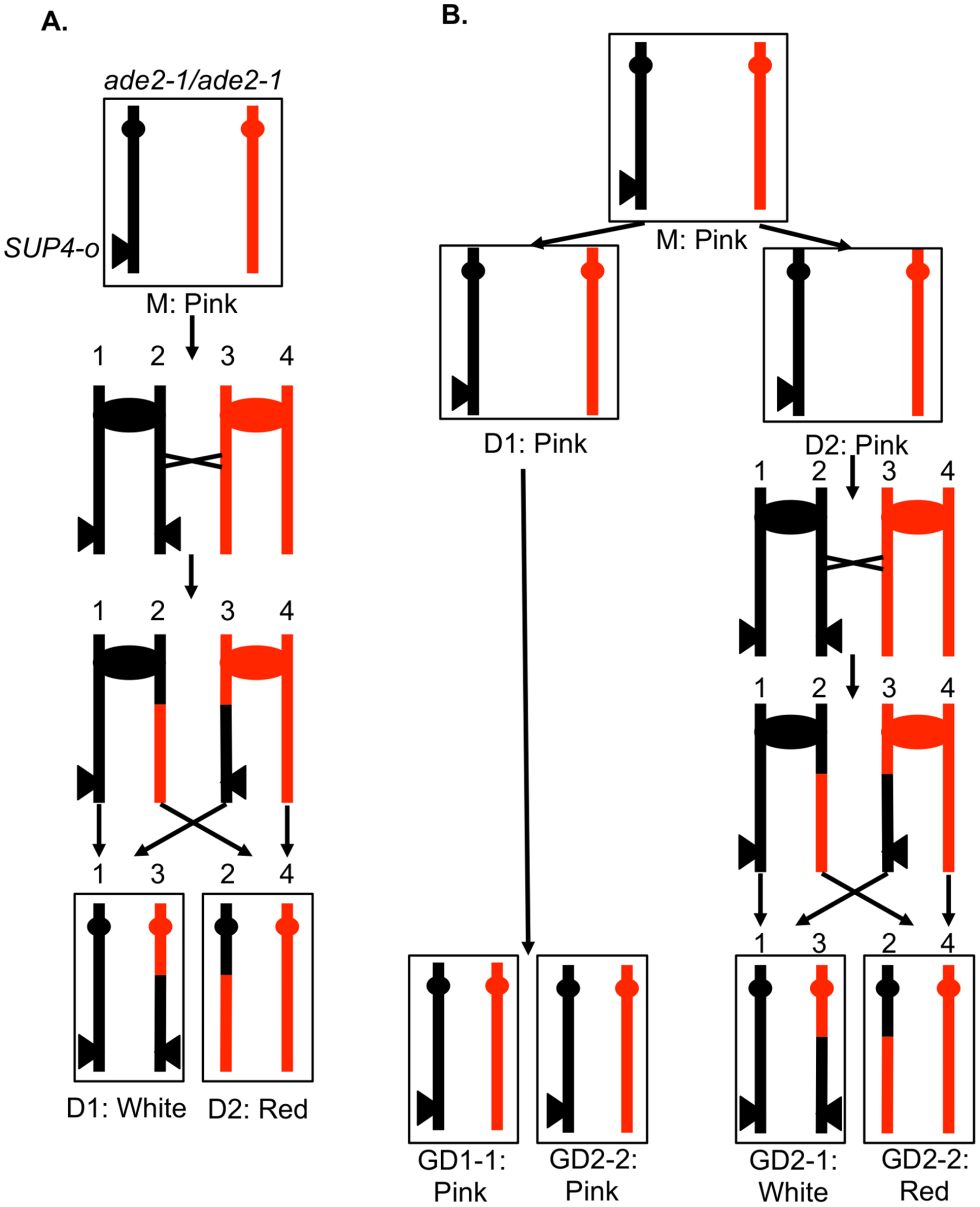
A system for detecting mitotic crossovers by a colony sectoring assay. G1-synchronized diploid cells were treated with UV and immediately plated on solid medium. The diploid is homozygous for the *ade2-1* mutation, an ochre mutation that when unsuppressed results in a red colony. The diploid has one copy of the ochre suppressor gene *SUP4-o* inserted near the telomere of chromosome IV on the black homolog. Strains with zero, one, and two copies of *SUP4-o* form red, pink, and white colonies, respectively. A. Crossover in G2 of the first division following irradiation. A DSB in one chromatid repaired during G2 will generate a red/white sectored colony, the white sector derived from daughter cell 1 (D1) and the red sector derived from daughter cell 2 (D2). B. Crossover delayed to G2 of the second division. If DNA damage induced in G1 is not repaired during the first division, a pink/white/red sectored colony would be generated. The abbreviation “GD” indicates the granddaughter of the irradiated cell.

In order to determine different types of mitotic recombination and to determine whether the conversion events are of the 3∶1 or 4∶0 configuration, we used a method of identifying recombination events that allows the recovery of both daughter cells with the recombinant chromosomes. The system used in the present study (Figure 3) is similar to that employed previously [2], [28]. Near the telomere of chromosome V, one homolog (shown in black in Figure 3A) has an insertion of *SUP4-o*, an ochre-suppressing tRNA gene. The diploid is also homozygous for the *ade2-1* ochre mutation. Diploids homozygous for the *ade2-1* mutation and zero, one or two copies of *SUP4-o* form colonies that are red, pink, and white, respectively [28].

In most of the experiments described below, G1-synchronized diploid cells were plated and immediately irradiated with UV. If the resulting DNA damage induces a crossover between the heterozygous *SUP4-o* gene and the centromere of chromosome V before the first cell division, a red/white sectored colony will be formed (Figure 3A). Since formation of a sectored colony requires a crossover, followed by the segregation pattern in which each daughter cell receives one recombined chromosome and one unrecombined chromosome (Figures 2A and 3A), only half of the crossovers induced in the first division following irradiation result in LOH. If the UV-induced DNA damage is not repaired in the first cell cycle but persists into subsequent cell cycles, a pink/white/red sectored colony could be produced (Figure 3B). As described below, most of the events induced by UV treatment in G1-synchronized cells generate a red/white sectored colony rather than a tri-colored colony. Neither gene conversion events unassociated with a crossover nor BIR events on chromosome V result in a red/white sectored colony. As will be shown below, such events can be detected as unselected events in cells that have a selected crossover on chromosome V.

The transition between heterozygous markers and homozygous markers in the sectored colony locates the position of the crossover. To detect the position of the selected crossover on chromosome V and to detect unselected LOH events throughout the genome, we used a diploid strain (PG311) derived from mating two sequence-diverged haploid strains: W303a and YJM789 [2], [7], [29]. These two strains differ by about 52,000 SNPs. We detect LOH using microarrays that examine 13,000 of these SNPs [7], allowing mapping of most events to a resolution of about 1 kb. Each SNP is represented by four 25-bp probes, two with W303a sequences (Watson and Crick) and two with YJM789 sequences. At the hybridization temperature optimized for the whole probe set, W303a genomic DNA hybridizes strongly to W303a oligonucleotides with very weak cross-hybridization to the corresponding YJM789 oligonucleotides, and vice versa for YJM789 genomic sequences. Genomic DNA is isolated from each sector of red/white sectored colonies, labeled with Cy5-tagged nucleotides, and competitively hybridized to the SNP microarray with genomic DNA from the untreated strain labeled with Cy3-tagged nucleotides. By assaying the ratio of hybridization of the differentially-tagged samples to each oligonucleotide [7], we can readily map LOH events. The transition between heterozygous and homozygous markers should be located near the site of the recombinogenic DNA lesion.

**Figure 4:**
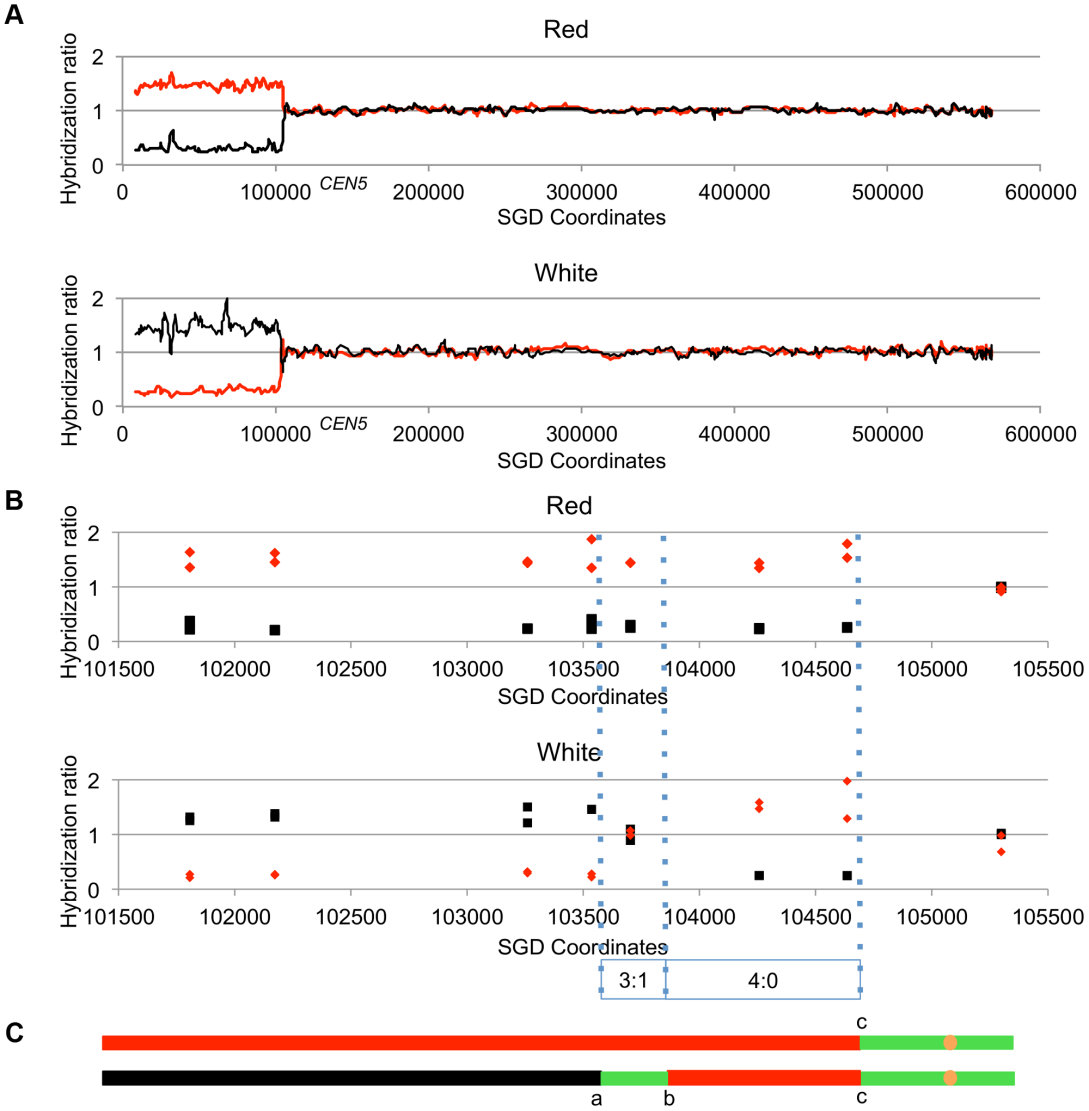
Mapping crossovers on chromosome V by SNP arrays. Genomic DNA was isolated from the red and white sectors of a sectored colony derived from UV-treated cells. The ratio of hybridization of SNP-specific oligonucleotides (relative to the hybridization levels of DNA from a fully heterozygous strain) for each sample was measured (details in text) and is shown on the Y axis. A hybridization level of about 1 indicates that the strain was heterozygous. The X axis shows the SGD coordinates of chromosome V beginning at the left telomere. Black and red lines indicate the normalized hybridization ratio to the YJM789- and W303a-specific oligonucleotides, respectively. The crossover and conversions associated with the sectors are diagrammed with the upper and lower panels showing patterns of LOH in the red and white sectors, respectively. A. Low-resolution depiction of the LOH events in the red and white sectors. The transition is at about SGD coordinate 105 kb. B. High-resolution depiction of LOH events (same event as shown in Figure 4A). Black squares show hybridization to YJM789-specific oligonucleotides and red diamonds show hybridization to W303a-specific oligonucleotides. The red sector has a single transition between heterozygous and homozygous SNPs, whereas the white sector has three transitions. The pattern of transitions is consistent with a 3∶1/4∶0 hybrid tract associated with a crossover. C. Summary of patterns of heterozygous and homozygous markers with the top line showing the red sector and the bottom line showing the white sector. The black, red, and green lines indicate regions homozygous for YJM789-derived SNPs, homozygous for W303a-derived SNPs, and heterozygous regions, respectively. The orange circles show the position of the centromeres. The lengths of the line segments showing the LOH region associated with the crossover and the heterozygous region centromere-proximal to the crossover are not shown to scale.

Figure 4 shows the analysis of one red/white sectored colony (59RW). In this figure, we show the normalized ratio of hybridization of genomic sequences to W303a- and YJM789-specific oligonucleotides on chromosome V with red lines and black lines, respectively; *CEN5* is located near coordinate 152 kb. In the top part of Figure 4A, we depict the pattern of hybridization of genomic DNA isolated from the red sector. The ratio of hybridization is about 1 for all SNPs from coordinate 105 kb to the right telomere, indicating that SNPs in this region are heterozygous. In the red sector, SNPs centromere-distal to coordinate 105 kb on the left arm are homozygous for the W303a-derived SNPs whereas the genomic DNA from the white sector becomes homozygous at approximately the same position for YJM789-derived SNPs. In Figure 4B, the same recombination event is depicted at higher resolution; each square and diamond shows the level of hybridization to an individual YJM789-specific or a W303a-specific SNP, respectively. As shown in this figure, the red sector has a single transition between heterozygous and homozygous SNPs whereas the white sector has three transitions. The pattern of these transitions indicates that the crossover is associated with a 3∶1/4∶0 hybrid conversion tract.

### Analysis of LOH events induced by UV-irradiation of cells with a dose of 15 J/m^2^

Most of our experiments involve UV treatment of G1-synchronized cells with 15 J/m^2^; the experimental parameters used for each experiment are in Table S1. PG311 is hemizygous at the *MAT* locus (*MAT* **a** */MAT*α::*NAT*), allowing its synchronization in G1 using the alpha pheromone [11]. The synchronized cells were plated onto solid medium and immediately irradiated at doses varying between 1 and 15 J/m^2^. Even at the maximum dose of UV, cell viability was 70%. No sectored colonies were observed in cells that were not treated with UV. Based on our earlier study of spontaneous crossovers in the same strain [2], the rate of crossovers in untreated cells is 1.1×10^−6^/division in the 120 kb interval between *CEN5* and the *SUP4-o* marker. Relative to this rate, UV treatment stimulated sector formation by factors of 1500 (1 J/m^2^), 1600 (5 J/m^2^), 5000 (10 J/m^2^), and 8500 (15 J/m^2^). The strong stimulation of mitotic crossovers by UV is consistent with previous studies [23].

In some studies [2], [4], [28], the frequency of mitotic recombination events is higher in diploids that express both mating types than in diploids that express only one mating type. Consequently, we compared the frequency of red/white sectored colonies in G1-synchronized cultures of PG311 and PSL101 (the *MAT* **a** */MAT*α progenitor of PG311). Because PSL101 cannot be synchronized in G1 using alpha pheromone, both strains were synchronized in G1 by growing the cells into stationary phase (Text S1). After treatment of the G1-synchronized cells with 15 J/m^2^ of UV, 0.4% (0.2–0.9%, 95% confidence limits) of the PG311 colonies formed red/white sectors compared to 0.6% (0.4–1%) of the PSL101 colonies. Although the confidence limits are wide, these results indicate that mating type heterozygosity does not have a large effect on the frequency of UV-induced mitotic crossovers in our system.

In addition to red/white sectored colonies, in the irradiated samples, we also observed pink/red and pink/white/red colonies. Such colonies could represent non-reciprocal recombination events (for example, BIR events), persistence of recombinogenic DNA damage beyond the first cell cycle, or an artifact (two closely-located independent cells). To exclude sectors formed artifactually, we micromanipulated individual G1-irradiated (15 J/m^2^ dose) single cells to specific positions on plates with solid medium, and monitored their subsequent development to form sectored or unsectored colonies. From a total of 970 isolated irradiated single cells, we observed eleven sectored colonies of the following types: seven red/white colonies, two pink/red colonies, and two pink/white/red colonies. From our SNP microarray analysis of the LOH patterns on chromosome V in these colonies (described in Text S1 and Figure S1), we found that all seven of the red/white colonies represented crossovers induced during the first cell cycle. The two pink/red sectored colonies reflected chromosome loss, resulting in a monosomic red sector and a pink sector. Only one of the pink/white/red colonies was a consequence of a UV-induced recombination event in the second division (Figure 3B, Figures S1 and S2). In summary, of the nine sectored colonies in which sectoring reflected a UV-induced crossover, eight occurred prior to the first cell division and only one occurred after the first cell division, indicating that most UV-induced DNA lesions are rapidly repaired.

We used SNP microarrays to analyze 47 sectored colonies of G1-synchronized cells treated with 5, 10 or 15 J/m^2^ of UV (Tables S2 and S3). 80% of the colonies were from cells treated with 15 J/m^2^. Nine of these colonies were derived from the single-cell experiments described above. 45 of the 47 sectored colonies examined had patterns of LOH on chromosome V consistent with a reciprocal crossover on the left arm of chromosome V. In one of the two exceptional colonies, there was a loss of one copy of chromosome V. In the other colony, there were two independent conversions that resulted in LOH events that were unassociated with a crossover. These two sectored colonies were not used in our subsequent analysis of selected events on chromosome V, although data from these colonies were used to analyze unselected recombination events.

In addition to the selected LOH events on chromosome V, we observed an average of eight unselected LOH events per sectored colony. As described below, our analysis of the 45 selected and 381 unselected events (300 gene conversion events unassociated with crossovers, 60 crossovers, and 21 BIR events) allowed us to determine several important features of the UV-induced recombination events: 1) the patterns of gene conversion in selected and unselected recombination events, 2) the lengths of gene conversion tracts associated or unassociated with crossovers, and 3) the locations of selected and unselected recombination events induced by UV. Since the frequency of selected sectored colonies in cells irradiated with 15 J/m^2^ was about 1%, and the selected interval on chromosome V is about 1% of the genome, we expect about one unselected crossover per irradiated cell, roughly the observed frequency (60 unselected crossovers/47 sectored colonies).

#### Patterns of gene conversion in selected and unselected recombination events

Our conclusions are based on SNP analysis of both sectors of the sectored colonies. As we have done previously [7], [29], the data from each sectored colony are presented as a pair of lines. For events on chromosome V, the top line represents the pattern observed in the red sector and the bottom line represents the white sector. Within each line, the three possible outcomes for SNPs (heterozygous, homozygous for the YJM789-derived SNP, and homozygous for the W303a-derived SNP) are shown by green, black, and red segments, respectively. Within each line, transitions between different colors are designated by a small letter. For example, the red sector of the colony in Figure 4C is shown as a line with two colors and one transition, whereas the white sector has three colors and three transitions. The transition labeled “c” is at the same position in both sectors. All recombination events, both selected and unselected, for cells irradiated with 15 J/m^2^ are depicted in an analogous manner in Table S2. The sectored colony in Figure 4 is 59RW and the pattern of the associated gene conversion events is given in Class I7 of Table S2. The SGD coordinates for each transition in Table S2 are given in Table S3; depictions and coordinates for one pink/white/red tri-sectored colony are shown in Tables S4 and S5.

**Figure 5:**
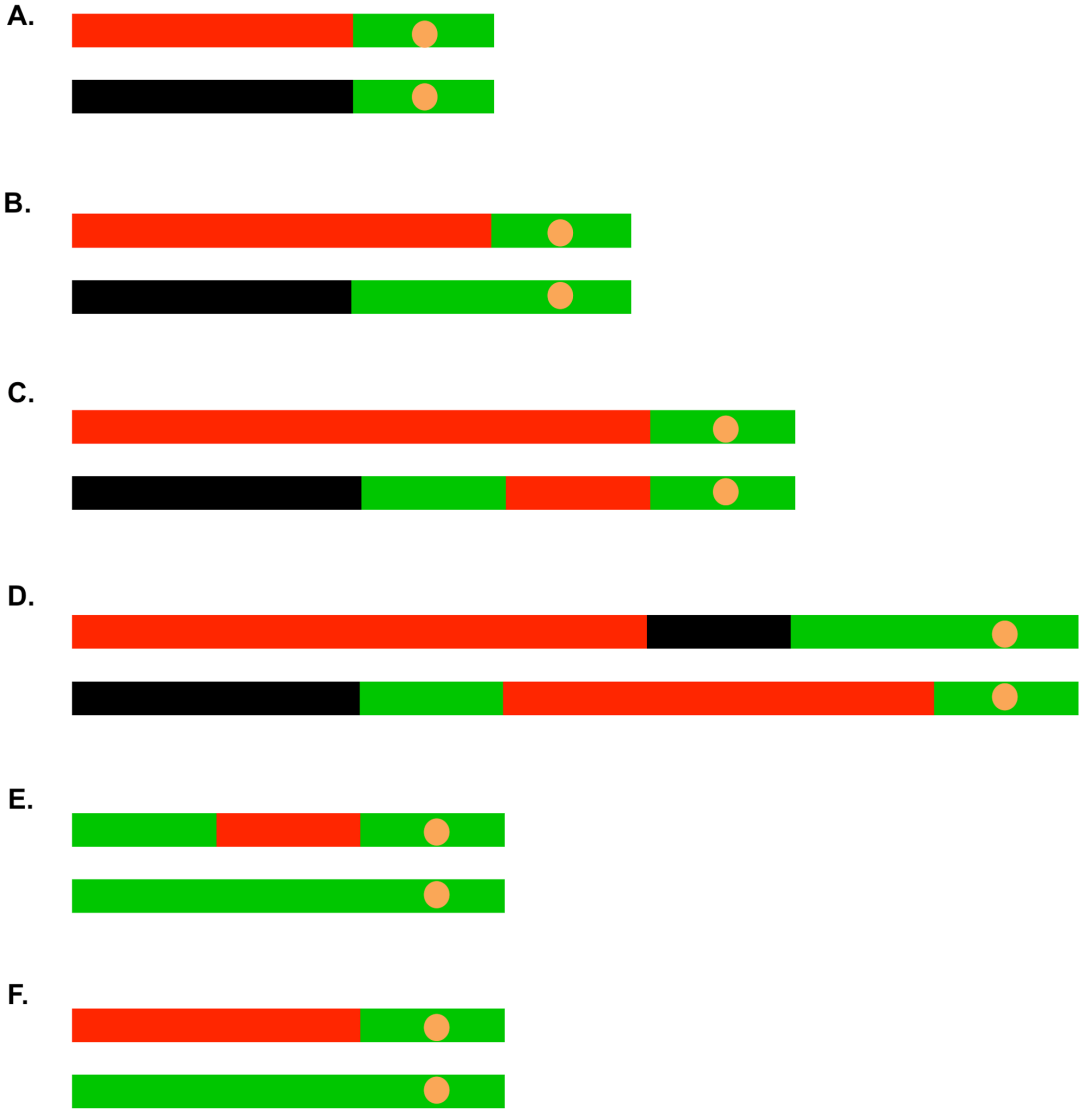
Common patterns of conversions and crossovers derived from UV-treated cells. The key is described in Figure 4C. A. Simple crossover unassociated with conversion. B. Crossover associated with a 3∶1 conversion event. C. Crossover associated with a 3∶1/4∶0 hybrid conversion tract. D. Complex crossover/conversion event. E. 3∶1 conversion unassociated with a crossover. F. BIR event.

Most (41 of 45) of the selected crossovers were associated with gene conversion events. In Figure 5, we show some of the common LOH patterns observed for the selected crossovers as well as for unselected recombination events. These depictions include a crossover unassociated with gene conversion (Figure 5A), a crossover associated with a 3∶1 conversion event (Figure 5B), a crossover associated with a 3∶1/4∶0 hybrid tract (Figure 5C), a crossover associated with a complex conversion tract (Figure 5D), a 3∶1 conversion unassociated with a crossover (Figure 5E), and a BIR event (Figure 5F). It should be noted that the patterns of gene conversion in sectored colonies can be used to infer which homolog was broken to initiate the recombination event. A chromosome that receives a DSB acts as a recipient during gene conversion [4]. Thus, in the event shown in Figure 5B, in which there is a red segment opposite a green segment, we can infer that the event was initiated by breaking the YJM789-derived (black) homolog.

As in our related studies [2], [7], [11], [29], we interpret the patterns of gene conversion and crossovers shown in Table S2 in the context of known pathways of DSB repair (Figure 1). All of these pathways involve regions of heteroduplex formation and, therefore, regions of duplex DNA that may contain mismatches. In wild-type strains, mismatches are usually efficiently removed by the mismatch repair (MMR) enzymes. In general, during mitotic recombination, MMR in yeast occurs directionally, removing the mismatched bases from the strand contributed by the broken chromosome [30]. In Figure 1A, the mismatches within the heteroduplex shown in the boxed region would usually be corrected to generate two red strands; this pattern of correction is termed “conversion-type” repair. Correction to generate two black strands for this heteroduplex is “restoration-type” repair. Also, in heteroduplexes involving multiple mismatches, all mismatches are removed from one strand with the other strand being used as a template for repair [30], although less frequent “patchy” repair events have also been described [7], [29]. For our study, we assume that the recombination events reflect the repair of one chromatid or two sister chromatids broken at the same position by the pathways shown in Figure 1. For the simple events, we assume non-patchy conversion-type repair and we assume that there is no branch migration of Holliday junction intermediates. Of the 300 conversion events induced by 15 J/m^2^ of UV, 276 were simple events, and of the 104 crossover events (most associated with conversion), 67 were simple events. The more complex events usually involve multiple transitions between heterozygous and homozygous SNPs and can often be explained as a consequence of patchy repair within a heteroduplex and/or branch migration. More detailed descriptions of complex events are given in Text S1.

As discussed in the Introduction, the patterns of gene conversion also provide important information about the timing of the recombinogenic DNA lesions during the cell cycle. In general (exceptions discussed in Text S1), 3∶1 conversion events likely reflect a single DSB producing a single broken chromatid in S or G2 of the cell cycle that was repaired in G2 (Figure 2A). Conversions with a 4∶0 tract or a 4∶0 segment of a more complex conversion tract likely reflect a DSB formed in G1. If such a broken chromosome is replicated to produce two broken chromatids that are then repaired in G2, a 4∶0 conversion tract would be generated (Figure 2D). We interpret 3∶1/4∶0 hybrid tracts of the type shown in Figure 4 as reflecting a G1 DSB. Below, we will refer to the conversion events that reflect a G2 DSB as single-chromatid breaks (SCBs) and those that reflect a G1 DSB as double-sister-chromatid breaks (DSCBs).

From most previous studies, we expected UV to generate SCBs. Removal of a pyrimidine dimer, followed by replication of the resulting DNA molecule before the gap was repaired would be expected to result in a G2 DSB [20]. Alternatively (or, in addition), since unrepaired pyrimidine dimers are recombinogenic [24], a DSB at the replication fork could occur; this lesion would also be expected to result in an SCB. In a preliminary study involving three UV-induced sectored colonies, however, we found about half of the events were DSCBs. In our current study, of 300 conversion events unassociated with crossovers (Classes A–G in Table S2), 167 (56%) were SCBs and 133 (44%) were DSCBs; these classifications are shown in Table S2 with the rationale for the classifications in Text S1. Of the 92 conversions associated with crossovers (selected and unselected), 35 (38%) were SCBs and 57 (62%) were DSCBs; the distribution of SCBs and DSCBs is significantly (p = 0.005) different than that observed for the conversions unassociated with crossovers. Our analysis supports the surprising conclusion that about half of the mitotic recombination events stimulated by high (15 J/m^2^) doses of UV in G1-synchronized cells involve two sister chromatids broken at the same position. As discussed below, a different result is obtained when cells are irradiated at low (1 J/m^2^) UV doses.

#### Recombination events induced by UV in G2-enriched cells

In addition to the comprehensive analysis of UV-induced recombination events in G1-synchronized cells, we did a preliminary analysis of the recombination events stimulated by 15 J/m^2^ UV in cells synchronized in G2. In one experiment, we examined the colonies derived from 2093 nocodazole-treated large-budded cells that were manipulated to defined locations on solid medium before irradiation. Although pink/red, and pink/white/red sectors were observed, we found no simple red/white sectors. The frequency of red/white sectors in G1-synchronized cells at the same radiation dose was 8/970, suggesting that the frequency of crossovers on chromosome V is at least 10-fold less in G2-irradiated cells than in G1-irradiated cells.

Because of the low frequency of events on chromosome V, we examined UV-induced recombination in G2 cells of the strain JSC25 in which the *SUP4-o* reporter gene is located near the end of chromosome IV [29]; the *CEN4-SUP4-o* interval is about eight-fold larger than the *CEN5-SUP4-o* interval. The frequency of red/white sectored colonies in G1-synchronized JSC25 cells irradiated with 15 J/m^2^ was 9×10^−2^ (127 sectors out of 1420 colonies). About 1% of the colonies formed red/white sectors in G2 cells synchronized with nocodazole (G2-A experiment) or in unsynchronized cells in which large budded cells were manipulated to defined positions before irradiation (G2-B experiment). We used microarrays to examine six and seven sectored colonies derived from the G2-A and G2-B experiments, respectively. Although we anticipated that most of the LOH events induced by G2 radiation would represent SCB conversions, among the G2-A LOH events, most (14 of 15) resembled DSCB conversions; among the G2-B LOH events, DSCB and SCB conversions were approximately equal in frequency (20 DSCB and 17 SCB). These results are difficult to interpret for a variety of reasons. First, the two protocols for obtaining G2 cells gave significantly different results; it should be pointed out that the nocodazole-induced synchronization is usually incomplete, with about 80–90% of the cells with the correct morphology [31]. Second, it is possible that nocodazole can induce LOH events. Third, some putative G2 cells, identified as having a doublet morphology, may not have completed the S-period. For all these reasons, we conclude that G2-enriched cells have lower levels of UV-induced recombination events than G1 cells, but we make no definitive conclusions about the nature of these events.

#### Lengths of gene conversion tracts associated or unassociated with crossovers

From the data shown in Tables S2 and S3, we derived the lengths of gene conversion tracts in the samples irradiated with 15 J/m^2^. We measured tract length by averaging the maximal length (the distance between heterozygous SNPs flanking the LOH region) and the minimal length (the distance between the first and last LOH SNP within the tract). For hybrid or complex tracts, regions both 4∶0 and 3∶1 segments were included as part of a single tracts as were short heterozygous regions within the tract. The median length of all conversion tracts (95% confidence limits in parentheses) was 5.6 kb (5.0–6.2 kb). The median length of conversion tracts associated with crossovers was 7.6 kb (6.4–9.6 kb); this median length is similar to that observed previously for spontaneous crossover-associated conversions (6.1 kb; [7]). Conversion tracts unassociated with crossovers were significantly shorter than crossover-associated conversions, 4.9 kb (4.1–5.6 kb) (p<0.00001; Mann-Whitney test). As noted previously, the lengths of mitotic conversion tracts are considerably larger than the average meiotic conversion tract (2 kb; [1]).

#### Location of LOH events induced by UV on chromosome V and throughout the genome

**Figure 6:**
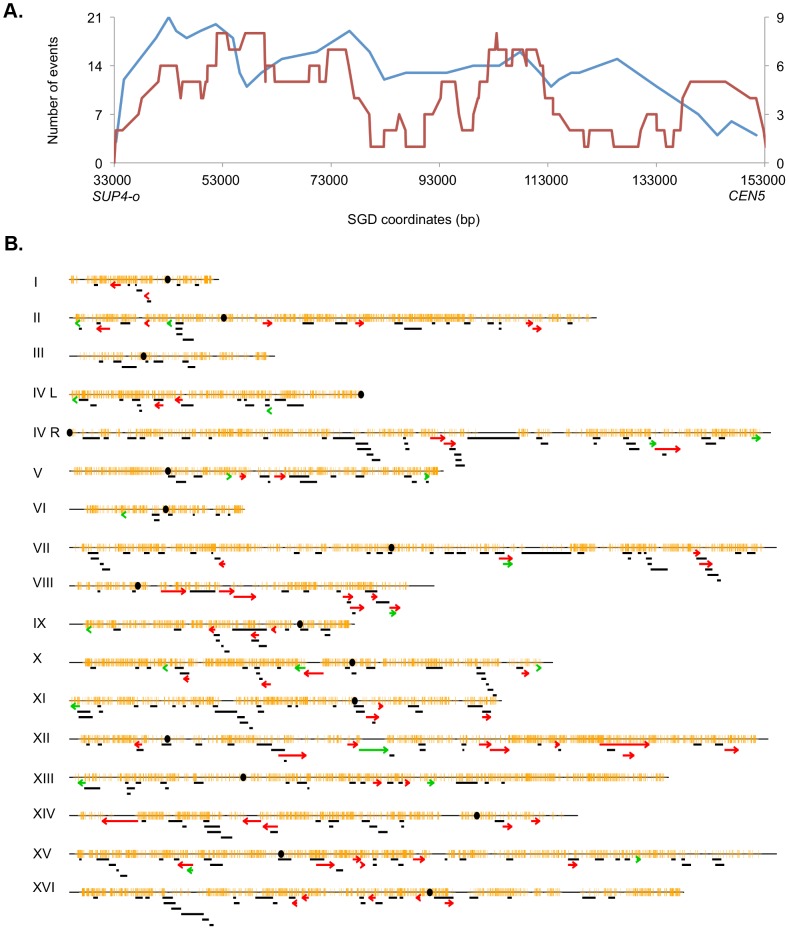
Patterns of selected events on chromosome V and unselected events throughout the genome. A. Selected events on the left arm of chromosome V. All events were based on red/white sectored colonies. The blue and red lines represent spontaneous [2] and UV-induced (15 J/m^2^), respectively. The X-axis shows the number of events that include specific SNPs with the left scale indicating the number of spontaneous events and the right scale showing the number of UV-induced events. The Y axis shows the SGD coordinates on V between the *can1-100/SUP4-o* markers (near coordinate 33 kb) and *CEN5* (near coordinate 152 kb). B. Location of unselected conversion and crossovers. Events on the left arm of chromosome V are not shown, since these events were selected. Centromeres are shown as black circles, and SNPs are indicated as short orange bars. Gene conversion tracts unassociated with crossovers are depicted by black line segments, with the length of the line approximately equivalent to the tract length. Red arrows show the positions of conversions associated with crossovers, and BIR events are indicated by green arrows. The lengths of the chromosomes are shown to scale; chromosome I is about 230 kb.

The pattern of UV-induced crossovers in the 119 kb interval between the *SUP4-o* marker and *CEN5* is shown in Figure 6A. In this figure, the X-axis shows SGD coordinates between *SUP4-o* (33 kb) and *CEN5* (152 kb). The Y-axes show the number of times individual SNPs were included within conversion tracts associated with crossovers with the blue and red lines indicating spontaneous [2] and UV-induced events, respectively. The number of times that a specific SNP will be included within a conversion tract is a function of the frequency with which recombination initiates near the SNP and the length of the conversion tract emanating from the initiating lesion [29]. To simplify the analysis of the distribution of recombination events, we determined the mid-point of each conversion tract. We then determined whether the distributions of these midpoints were significantly different in spontaneous and UV-induced events by comparing the numbers of events in four equal-sized intervals (33–63 kb, 63–93 kb, 93–123 kb, and 123–153 kb). By Fisher exact test, the distributions of spontaneous and UV-induced events are not significantly different. In addition, the distribution of UV-induced events on chromosome V is not significantly different from a random distribution.

The map positions of unselected LOH events in cells treated with UV doses of 15 J/m^2^ are shown in Figure 6B. There were 60 crossovers, 21 BIR events, and 300 gene conversion events unassociated with crossovers. These events are widely distributed throughout the genome with the number of LOH events correlating strongly with the size of the chromosome (r^2^ = 0.90, p = 2.4×10^−8^). Although none of the individual chromosomes, normalized for size, were significantly hot or cold for LOH events, the right arm of chromosome VIII had a significantly elevated frequency of crossovers as determined by chi-square analysis (p = 0.03), correcting for multiple comparisons by the method of Hochberg and Benjamini (1990) [32]. In addition, based on the total number of unselected crossovers in the dataset of Table S3 (60) and the fraction of the genome that is the rRNA gene cluster (10%), there is significant suppression of UV-induced crossovers within the rDNA; we expected six crossovers, but observed no crossovers. By chi-square analysis, this difference is significant (p = 0.02). Further support for this conclusion will be presented below.

We also analyzed whether certain chromosomal elements or sequence motifs are enriched or underrepresented within the conversion tracts of unselected recombination events. The details of this analysis are described in Text S1. When all conversion events are examined, there was a significant under-representation of long terminal repeats (LTR, p = 0.0003), and tRNA genes (p = 0.002); the p values remain significant after correction for multiple comparisons. We also examined the representation of these elements when SCB conversions and DSCB conversions were analyzed separately. In this analysis, non-coding RNAs were significantly over-represented among the DSCB class of conversions. The significance of these results will be examined in the Discussion.

We also observed a bias in the distribution of BIR events. Of the 21 events, eight were within 50 kb of the telomeres. Based on the genome size and the number of the telomeres, the expected number of BIR events within 50 kb of the telomeres is three. The difference between the observed and expected distribution is significant (p = 0.005). The interpretation of this observation will be discussed below.

Since the induction of recombination by UV is assumed to reflect the location of processed or unprocessed pyrimidine dimers and since most (68%) of the dimers formed involve TT [33], we examined the frequencies of AA/TT dinucleotides in unselected conversion events (0.218), selected conversion tracts on chromosome V in cells irradiated with 1 J/m^2^ (0.217) or 15 J/m^2^ (0.221). These frequencies were similar to those observed for the whole genome (0.217) or the left arm of chromosome V (0.219). In contrast, the frequency of AA/TT dinucleotides in the ribosomal DNA is very significantly (p<0.0001, Fisher exact test) reduced compared to the rest of the yeast genome, 0.190 compared to 0.217.

#### Analysis of UV-induced crossovers within the ribosomal DNA

**Figure 7:**
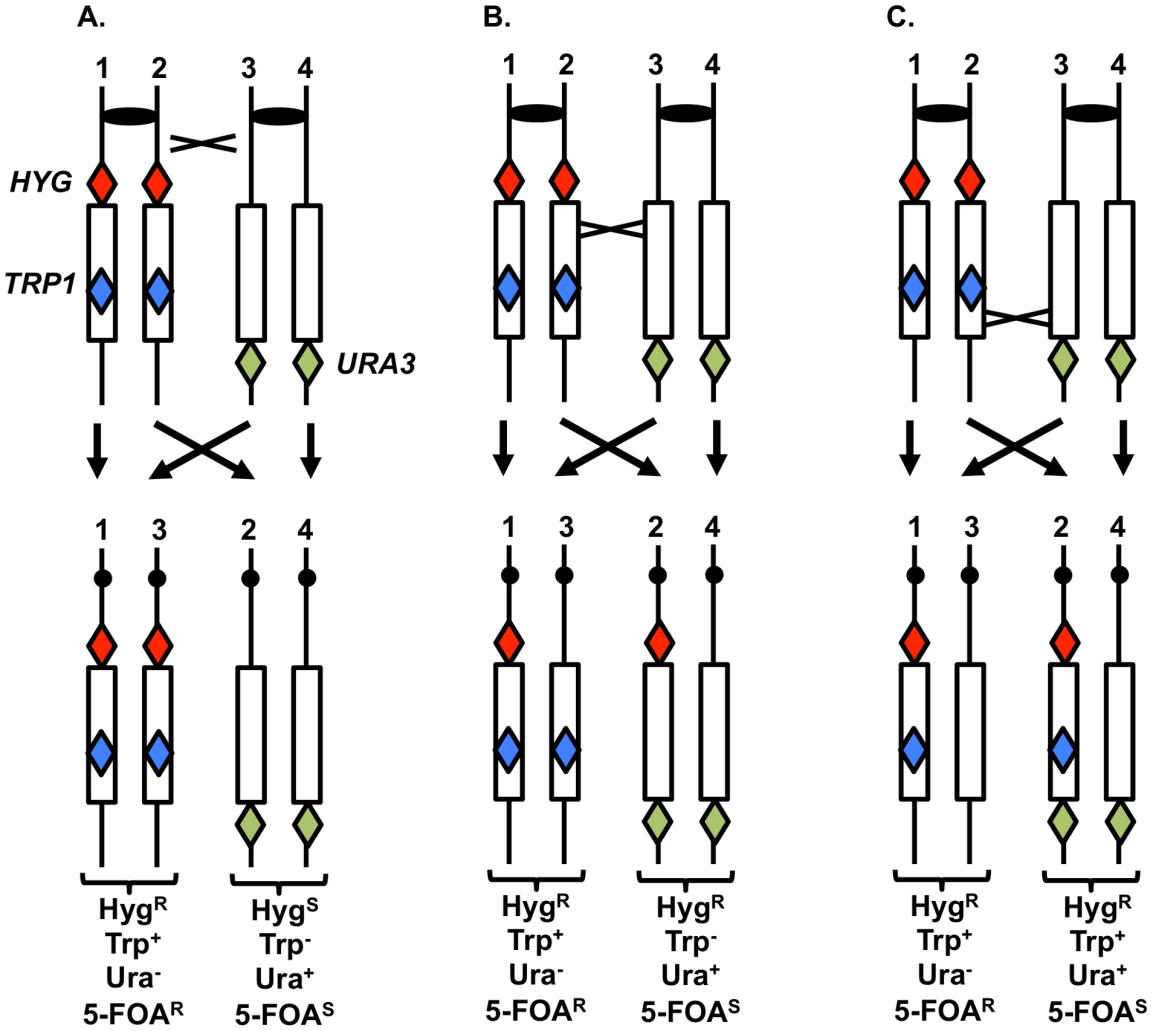
System to monitor crossovers on chromosome XII. A diploid was constructed with heterozygous markers immediately centromere-proximal to the ribosomal RNA (rRNA) gene clusters (*HYG*), within the rRNA gene cluster (*TRP1*), and immediately centromere-distal to the cluster (*URA3*) [34]. G1-synchronized cells were treated with UV, plated on solid medium and grown non-selectively. The resulting colonies were replica-plated to medium lacking uracil, tryptophan, or containing hygromycin as described in the text. A. Sectoring pattern expected for a crossover centromere-proximal to the *HYG* marker. B. Sectoring pattern expected for a crossover within the rRNA gene cluster centromere-proximal to *TRP1*. C. Sectoring pattern expected for a crossover within the rRNA gene cluster centromere-distal to *TRP1*.

To support the conclusion that UV treatment is less recombinogenic for the rDNA than for other genomic sequences, we analyzed UV-induced recombination in a diploid strain YYy13 with heterozygous markers flanking the rDNA cluster (*HYG* and *URA3*) and within the cluster (*TRP1*) (Figure 7); the *TRP1* gene within the cluster is located about 230 kb from the centromere-distal end of the cluster [34]. This strain is in a different genetic background than PG311, but is a *MATα* deletion derivative of AMC45, a strain in which mitotic crossovers were examined previously [34]. G1-synchronized YYy13 cells were irradiated with 15 J/m^2^ UV and plated non-selectively on omission medium. The resulting colonies were replica-plated to medium containing hygromycin, medium lacking tryptophan, and medium lacking uracil, and the phenotypes of sectored colonies were scored. Crossovers centromere-proximal to *HYG* result in one Hyg^R^ Trp^+^ Ura^−^ sector and one Hyg^S^ Trp^−^ Ura^+^ sector, whereas crossovers between *HYG* and *TRP1* produce one Hyg^R^ Trp^+^ Ura^−^ sector and one Hyg^R^ Trp^−^ Ura^+^ sector (Figures 7A and 7B). The expected sectoring pattern for a strain with a crossover in the rDNA between the *TRP1* insertion and the *URA3* marker is shown in Figure 7C.

In addition to sectors formed by crossovers, BIR events can also produce sectored colonies (Figure S3). One distinction between crossovers and BIR events is that the Ura^+^ sector of crossovers is homozygous for the *URA3/URA3* wild-type alleles, whereas for BIR events, the Ura^+^ sectors are often heterozygous *ura3/URA3*. These two types of Ura^+^ strains can be distinguished by replica-plating cells grown non-selectively to medium containing 5-fluoro-orotate (5-FOA). 5-FOA poisons cells with wild-type *URA3* activity. Heterozygous strains form 5-FOA^R^ papillae on medium containing 5-FOA because the wild-type allele is readily lost by mitotic recombination or mutation; in contrast, strains that are homozygous for the wild-type allele very rarely produce 5-FOA^R^ papillae [34].

A third type of mitotic recombination that can be detected in irradiated YYy13 cells is intrachromosomal exchange. In this type of event, sectoring is observed for the *TRP1* marker, but not the flanking *HYG* and *URA3* markers (Figure S4). Loss of the *TRP1* marker could reflect either single-strand annealing [4], unequal crossing-over between sister chromatids, or “pop-out” of *TRP1* by an intrachromatid crossover flanking the insertion. In summary, by testing growth of the sectors on a variety of types of media, we can distinguish whether sectored colonies reflect crossovers, BIR events, or intrachromosomal exchanges (details in Text S1).

In addition to determining the frequencies of UV-induced recombination events in YYy13, we also determined the frequencies of spontaneous events. The summary of crossovers, BIR, and intrachromosomal events is shown in Table 1. In a sample of 3902 colonies derived from G1-synchronized irradiated cells, we observed 49 crossovers in the 300 kb *CEN12-HYG* interval and 92 crossovers in the 1170 kb *HYG-URA3* interval containing the rRNA gene cluster. After correcting for the expected number of spontaneous events and dividing the frequencies of crossovers by the size of the interval, we calculate that the frequencies of UV-induced crossovers in the *CEN12-HYG* and *HYG-URA3* interval are 8.2×10^−5^/kb and 3.8×10^−5^/kb, respectively. The frequency of UV-induced crossovers in the 120 kb *CEN5* to *SUP4-o* region is 1.5×10^−4^/kb. These results demonstrate that the frequency of UV-induced crossovers within the rDNA per kb is about two to four times less than observed for two other large chromosomal intervals (*CEN12-HYG* and *CEN5-SUP4-o*). By chi-square analysis, the rDNA is significantly colder for UV-induced crossovers than the *CEN12-HYG* interval (p<0.0001).

**Table 1:** Numbers of colonies with spontaneous and UV-induced crossovers between *CEN12* and the ribosomal DNA and within the ribosomal DNA.

	*CEN12-HYG* interval			*HYG-TRP1* interval			*TRP1-URA3* interval			ICR	# colonies examined
	RCO	BIR-U	BIR-H	RCO	BIR-U	BIR-H	RCO	BIR-U	BIR-H		
UV-treated cells	49	9	2	77	16	9	15	8	(8)	116	3902
Untreated cells	1	0	0	5	2	1	0	1	(1)	6	5197
Normalized UV-treated^a^	48	9	2	73	14	8	15	7	(7)	111	3902

In this table, we show data obtained from spontaneous and UV-induced cells derived from the strain YYy13. As shown in Figure 7 and Figure S3, from examining the segregation of markers, we can determine the location of crossover and BIR events on chromosome XII by detecting colonies that form sectors on media lacking uracil or tryptophan or media containing hygromycin. BIR-U and BIR-H indicate that the BIR event was initiated by a DSB located on the *URA3-* or *HYG*-containing chromosome XII homolog, respectively. Since a BIR event initiated on the *HYG*-containing chromosome between *TRP1* and *URA3* does not result in sectors on any of the media, the number of events in this category (shown in parentheses) was assumed to be the same as the number on the *URA3*-containing chromosome. The ICR column shows the number of intrachromosomal events (intrachromatid single-strand annealing, intrachromatid “pop-outs” or unequal sister-chromatid exchanges) as shown in Figure S4.

a To calculate the normalized number of UV-induced events, we multiplied the number of observed events in the untreated cells by the ratio of the colonies examined for the untreated and UV-treated cells (3902/5197), and subtracted that value from the observed number of UV-treated cells in each category.

We estimated 11 and 36 BIR events in the *CEN12-HYG* and rDNA, respectively (Table 1). Although the frequency of BIR events per kb in the rDNA is slightly less than in the *CEN12-HYG* interval, the difference is not statistically significant. We found that intrachromosomal recombination events were also substantially elevated by UV, 116 events/3902 treated cells versus 6 events in 5197 cells not exposed to UV. We did not determine whether the intrachromosomal events were a consequence of unequal sister-chromatid crossovers, intrachromatid “pop-outs” or single-strand annealing events. A high frequency of spontaneous mitotic intrachromosomal recombination events in the rDNA was previously observed in yeast [34]–[37]. In summary, although UV treatment elevates the frequency of crossovers, BIR events, and intrachromosomal recombination events in the rDNA relative to that observed in unirradiated strains, the magnitude of the induction of crossovers in the rDNA is significantly less than for other regions of the genome.

### Analysis of LOH events induced by UV-irradiation of G1-synchronized cells with a dose of 1 J/m^2^

One interpretation of our observation of frequent DSCBs in G1-irradiated cells is that the repair of two very closely-spaced single-stranded DNA lesions induced by 15 J/m^2^ results in DSCBs in the G1-synchronized cells, whereas SCBs reflect DNA lesions on one strand. Thus, the productions of DSCBs by this mechanism would be proportional to the square of UV dosage, whereas the frequency of SCBs would be linearly proportional to the UV dosage. By this model (details to be discussed below), one might expect that a low dose of UV should have a relatively higher frequency of SCBs. Consequently, we examined the frequency and types of recombination events induced in G1-synchronized cells by 1 J/m^2^. As expected, the frequency of red/white sectored colonies was reduced in cells irradiated with 1 J/m^2^ relative to cells irradiated with 15 J/m^2^ (1.6×10^−3^/division versus 9.4×10^−3^/division). Ten sectored colonies were examined by whole-genome microarrays. Only four unselected events were observed. This frequency (0.4 events/sectored colony) was about twenty-fold less than that observed in samples irradiated with 15 J/m^2^ (8 events/sectored colony). Consequently, in the additional thirty-six sectored colonies examined, we used microarrays specific for detecting LOH on chromosome V.

The depictions of the LOH events in the 1 J/m^2^ irradiated samples that had the same patterns as observed for the 15 J/m^2^ samples are shown in Table S2; the numbers of samples with specific classes of events are shown in parentheses in this table. Patterns of LOH that were unique to the 1 J/m^2^ samples are shown in Table S6. The coordinates for these LOH events are shown in Table S7. The distribution of the LOH events on chromosome V for the 1 J/m^2^ samples was not significantly different from that observed for the 15 J/m^2^ samples or the spontaneous events using the same “binning” procedure and statistical test described above. The median length of conversion events associated with crossovers on chromosome V in cells irradiated with 1 J/m^2^ was 4.3 kb (2.3 kb–8.2 kb; 95% confidence limits) kb. In cells irradiated with 15 J/m^2^, the median length of conversion tracts associated with crossovers on chromosome V was 6.7 (4.2–13 kb). The distributions of tract lengths analyzed by the Mann-Whitney test showed that these distributions were not significantly different (p = 0.12).

**Figure 8:**
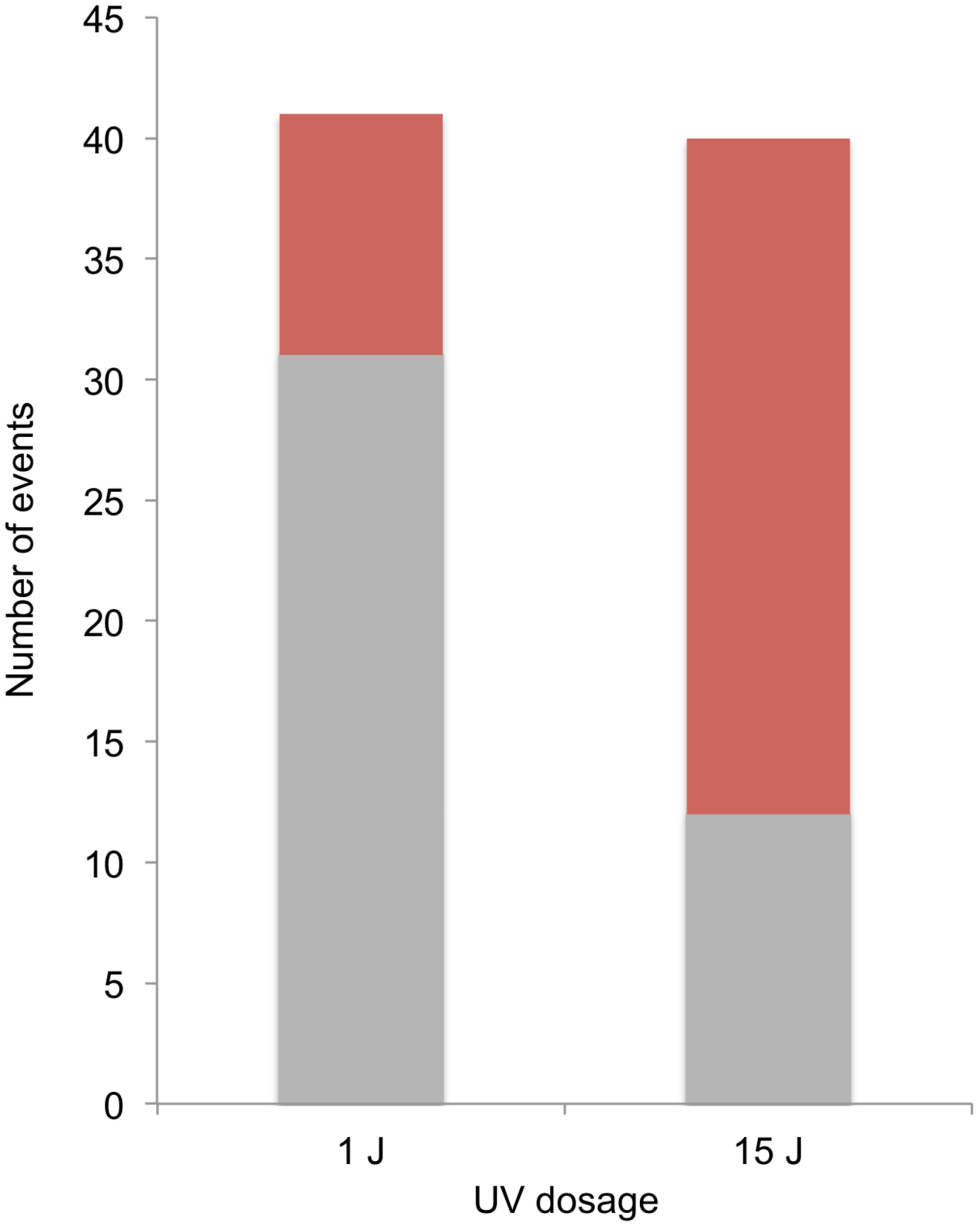
Selected SCB and DSCB conversions in strains treated with 1/m^2^ and 15 J/m^2^. SCB and DSCB events are indicated in gray and red, respectively.

A striking difference was observed in the distributions of events diagnostic of SCBs and DSCBs in cells irradiated with 1 and 15 J/m^2^ of UV. Of selected events on chromosome V in cells irradiated with 1 J/m^2^, we observed 5 crossovers unassociated with conversion, 31 SCB events, and 10 DSCB events. In contrast, in cells irradiated with 15 J/m^2^, most of the selected events on chromosome V were DSCB events (Figure 8). By the Fisher exact test, the difference in the numbers of SCB and DSCB events induced by the two different UV treatments is very significant (p<0.0001). The conclusion that G1-synchronized cells have different recombinogenic DNA lesions induced by different UV doses will be discussed further below.

## Discussion

Our main conclusions are: 1) UV induces high frequencies of crossovers and gene conversions in G1-synchronized yeast cells but is less recombinogenic in G2-enriched cells, 2) the LOH events induced by high doses of UV primarily involve the repair of two sister chromatids broken at the same position whereas most events induced by low doses (1 J/m^2^) involve repair of a single broken chromatid, 3) UV-induced LOH events are widely distributed throughout the genome, although some classes of repeated genes are significantly underrepresented, 4) most of the recombinogenic effects of UV in cells treated in G1-synchronized cells are manifested in the first cell cycle after irradiation, 5) in G1-synchronized cells, crossovers are induced about six-fold more frequently than BIR events, 6) about one-third of UV-induced conversion events are associated with crossovers, and 7) although UV effectively induces crossovers between homologs, chromosome rearrangements are not produced at detectable frequencies.

### Recombinogenic DNA lesions induced by UV

As noted in previous studies, UV very effectively induces mitotic recombination in yeast [7], [10], [20], [21], [23], [38]. In experiments involving heteroallelic recombination in synchronized cells, UV is somewhat more recombinogenic in G1-synchronized cells than in G2-synchronized cells [18], [21]; our results support these observations. Kadyk and Hartwell (1992) [24] concluded that DSBs induced by X-rays in G2-synchronized cells were repaired primarily by sister-chromatid recombination, whereas X-ray treatment of G1-synchronized cells effectively stimulated recombination between homologs. Our previous interpretation of both spontaneous and DNA damage-induced crossovers is also consistent with this conclusion [2], [7], [29]. We argue that most spontaneous crossovers between homologs are initiated by a DSB in G1 in one chromosome, and replication of the broken chromosome produces two sister chromatids broken at the same position. Since these lesions cannot be repaired by sister-chromatid recombination, they are repaired by recombination with the homolog. Although it is likely that DSBs formed in S or G2 are primarily repaired by sister-chromatid recombination, some DSBs generated in G2 are repaired by interaction with the homolog [11].

As observed in our previous studies [2], [7], [29], the mitotic conversion tracts are long compared to those observed in meiosis, and the tracts associated with crossovers are longer than the tracts unassociated with crossovers. Most of the conversion events are explicable as a consequence of repair of one broken chromatid or two sister chromatids broken at the same position by the standard HR pathways shown in Figure 1, with only conversion-type MMR and not restoration-type MMR. About 15% of the conversion events, however, are more complex, requiring “patchy” repair of mismatches within a heteroduplex (mismatches corrected by both conversion-type repair and restoration-type repair within one heteroduplex), and/or branch migration of the Holliday junction. The fraction of complex conversion tracts in the current study is similar to those observed in our previous studies [7], [29]. Although these events (described in detail in Text S1 and Figures S5, S6, S7, S8, S9, S10, S11, S12, S13, S14, S15) are explicable by modifications of the standard models shown in Figure 1, it is possible that some of these conversion events involve a substantially different mechanism such as multiple template switching events during BIR. In this context, template switching during BIR has been observed in experiments in which linear DNA fragments are transformed into yeast [39]. In addition to the complex tracts, it is possible that the very long conversion tracts reflect BIR rather than mismatch repair in a heteroduplex; 16% of the conversion events unassociated with crossovers are greater than 10 kb in length, and the longest exceeds 50 kb. Finally, it should be pointed that, although single BIR events would not be expected to generate crossovers, a model for production of a crossover by a double BIR event is shown in Figure S4 of Lee *et al.* (2009) [2].

**Figure 9:**
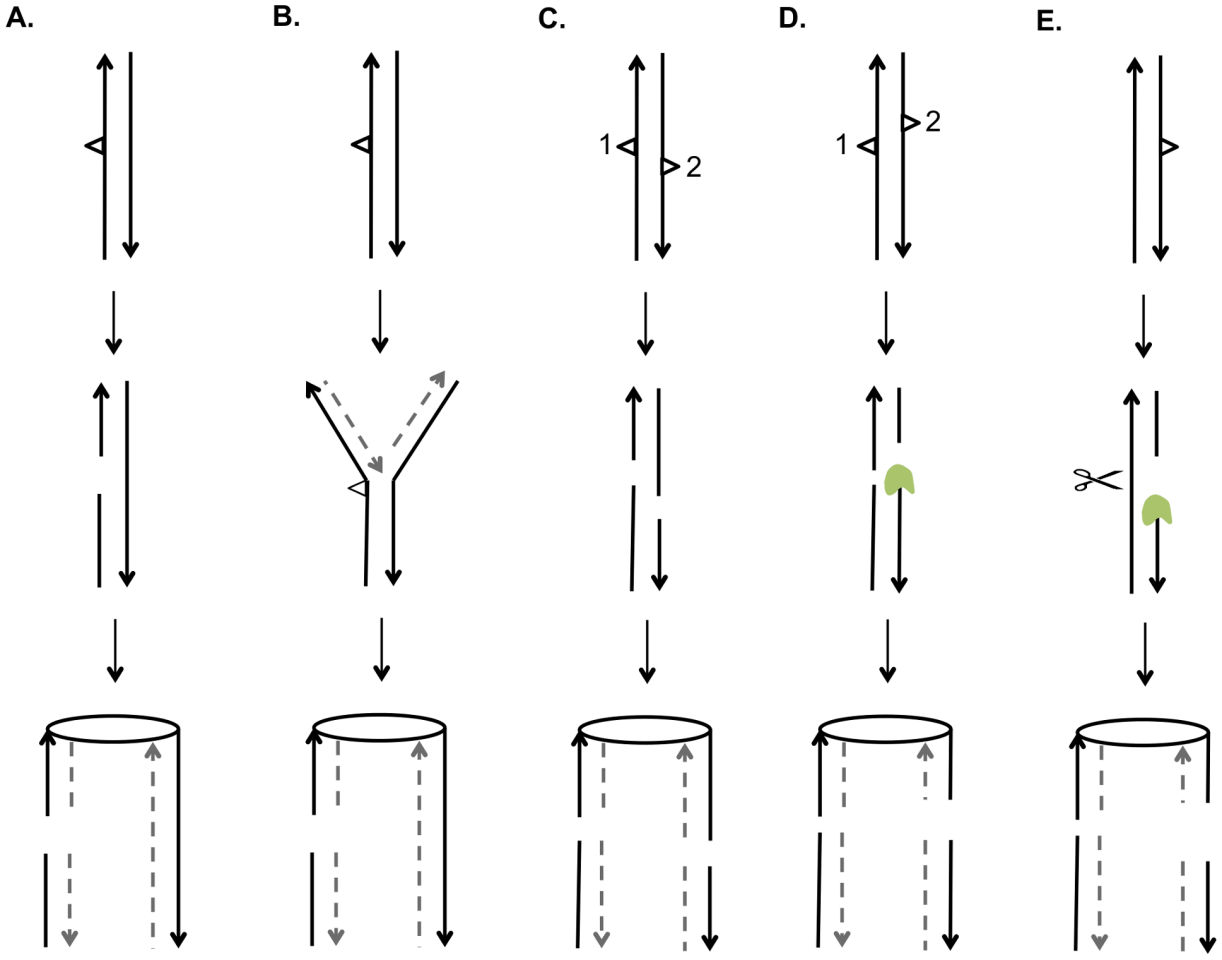
Mechanisms for generating UV-induced recombinogenic DSBs. At the top part of the figure, chromosomal DNA molecules are depicted as unreplicated double-stranded DNA molecules. Newly-synthesized DNA is depicted as gray dashed lines. UV-induced pyrimidine dimers are shown as triangles, and centromeres of replicated chromosomes are shown as ovals. A. Excision of a dimer results in a small gap and replication produces one broken and one unbroken sister chromatid. B. During replication of a DNA molecule with an unexcised dimer, a DSB occurs in one of the two sister chromatids. C. Excision of two closely-opposed dimers results in a short (<6 bp) unstable double-stranded region between the excision tracts. The resulting broken chromosome is replicated to form two broken sister chromatids. D. As in Figure 9C, two closely-opposed dimers are excised. One of the resulting short gaps is expanded by the 5′ to 3′ Exo1p nuclease (shown in green) to generate a broken chromosome. Replication of this chromosome results in two broken sister chromatids. E. The tract resulting form excision of a single dimer is expanded, leaving a large single-stranded DNA gap. An endonuclease cleaves this single-stranded region, resulting in two broken sister chromatids.

A central issue is the nature of the recombinogenic DNA damage generated by UV. Based on the mechanism of NER and on the observation that unrepaired pyrimidine dimers block replication, there are two obvious potential sources of DSBs [40]. First, if a DNA molecule with an unrepaired gap resulting from NER is replicated before filling-in of the gap and ligation, the net result would be a pair of sister chromatids with a single DSB (Figure 9A). Alternatively, if a replication fork encounters an unrepaired UV-induced lesion, breakage of the fork could also result in a single broken chromatid (Figure 9B). Based on the observation that UV treatment of G1- or G2-synchronized cells was not recombinogenic unless cells were allowed to divide, Galli and Schiestl (1999) [20] suggested that cell division was required to convert DNA lesions to recombinogenic lesions, consistent with both of the possibilities described above; their assay detected only intrachromatid deletions. Kadyk and Hartwell (1993) [21] found that unrepaired UV lesions stimulate gene conversion events between homologs, but have little effect on mitotic crossovers. This conclusion may be affected by the use of the *rad1* mutation to prevent dimer excision, since *rad1* strains have reduced frequencies of crossovers in some assays [41].

Both of the models discussed above predict that UV-induced DNA damage in G1-synchronized cells would produce primarily gene conversion events involving a single broken chromatid (SCBs). In our study, about two-thirds of the conversion events in which cells were irradiated with 15 J/m^2^ reflect two broken sister chromatids, but only one-quarter of the conversions reflect two broken sister chromatids in cells irradiated with 1 J/m^2^ (Figure 8). Thus, there is a qualitative change in the nature of the DNA lesion with increasing UV dose. In addition, since our single-cell experiments demonstrate that UV-induced lesions are recombinogenic during the first division following treatment, DSCBs cannot be explained as reflecting the segregation of a chromosome with an unrepaired G2-associated DSB from the previous division.

We suggest that most DSCBs are a consequence of a DSB in G1. Although UV damage is generally regarded as an agent that produces DNA nicks rather than DSBs, a gel-based detection of the conversion of a circular chromosome to a linear chromosome indicated that a dose of 40 J/m^2^ produces 5 to 10 DSBs in G2-synchronized cells [42]. There are several related mechanisms by which NER could produce a DSB in G1 cells. First, the excision tracts resulting from removal of two closely-opposed dimers could result in very short (<6 bp) unstable duplex regions between the repair tracts, resulting in a DSB (Figure 9C). A second model is that, following the removal of two closely-opposed dimers by NER, one or both of the resulting short gaps is expanded by Exo1p (Figure 9D). A third related model is that the excision tract generated by NER is expanded into a large single-stranded gap that is cleaved by an endonuclease to yield the DSB (Figure 9E).

Based on our results and those of others, it is likely that UV produces a variety of recombinogenic lesions. In our experiments, at a low dose of UV (1 J/m^2^), we observed primarily SCBs, consistent with the two models shown in Figures 9A and 9B. At 15 J/m^2^, we observed DSCBs more frequently than SCBs. This observation supports models shown in Figure 9C and 9D that require closely-opposed lesions, and argues against the model shown in Figure 9E in which the relative fraction of DSCB and SCB events would be expected to be independent of the density of the NER tracts. It can be calculated that diploid cell irradiated with 15 J/m^2^ have about 7500 dimers/genome [43]; if these dimers are distributed randomly, we expect about 35 closely-opposed (separated by ≤75 bases) dimers, enough to explain the detected DSCB events. It is likely that the number of closely-spaced dimers is greater than that determined by this calculation. Lam and Reynolds (1987) [43] found that the fraction of dimers located within 15 base pairs of each other is greater than expected from a random distribution, and this fraction is somewhat independent of UV dose. These dimers may be responsible for the DSCB events detected in strains treated with the 1 J/m^2^ UV dose.

In summary, we suggest that low doses of UV primarily result in SCBs as a consequence of replication of a chromosome with a NER-generated DNA gap in one strand, or an unrepaired dimer resulting in breakage of one arm of the replication fork. In contrast, we suggest that high doses of UV often result in DSCB events as a consequence of a G1-generated DSB, reflecting cellular enzymes acting on closely-opposed dimers. Although this explanation seems straightforward, we cannot exclude more complex explanations of our data. For example, it is possible that the very large number of UV-induced lesions at high doses may overwhelm the DNA repair systems, resulting in changes in the use of repair pathways. In addition, we stress that our analysis based on interhomolog recombination does not yield an estimate of the relative frequencies of UV-induced recombinogenic lesions produced in G1, S, and G2, since most recombinogenic lesions produced in S and G2 are likely repaired by sister-chromatid recombination [24], a mechanism that does not lead to LOH [7].

### Distribution of UV-induced LOH events

The UV-induced recombination events were broadly distributed throughout the genome; no strong recombination hotspots were detected. The distribution of UV-induced genomic LOH events is expected to be a function of multiple factors such as: 1) the distribution of DNA damage, particularly the distribution of closely-opposed dimers, 2) the relative frequency of dimer repair by recombinogenic and non-recombinogenic pathways, and 3) the relative frequency of repair of recombinogenic DNA damage by sister-chromatid recombination, non-homologous end-joining, and recombination between homologs. The regions on each chromosome that were examined for LOH events are in Table S8.

It has been shown recently that the distribution of UV-induced pyrimidine dimers observed *in vivo* in yeast is primarily a function of the density of TT, TC, CT, and CC sequences in the genome ([44], Sheera Adar and Jason Lieb, personal communication). As previously discussed, in cells irradiated with 15 J/m^2^, the calculated frequencies of AA/TT dinucleotides among our selected (0.221) and unselected (0.218) conversion events are very close to the frequencies on the left arm of chromosome V (0.219) and the whole genome (0.217). In contrast, the frequency of these dinucleotides in the ribosomal DNA (0.19) is very significantly less (p<0.0001) than the frequency in the whole genome. Thus, the observed reduction in UV-induced recombination in the ribosomal DNA, at least in part, may be a consequence of a reduced frequency of dimer formation. Interestingly, the two motifs that are underrepresented in the unselected conversion events (tRNA and solo LTRs in Table S9) also have frequencies of AA/TT dinucleotides that are considerably less than the genomic frequency (0.124 for tRNA genes and 0.205 for the solo LTRs). Since the tRNA genes and the solo LTRs are smaller than the average conversion tract size, however, it is unlikely that the relative lack of AA/TT dinucleotides is the only factor influencing the frequency of UV-induced conversion events that include these elements. Finally, we note that the frequency of AA/TT dinucleotides in non-coding RNA genes, which are significantly over-represented in the DSCB conversion tracts (Table S9), is also higher (0.23) than the frequency for the whole genome. A more detailed discussion of the relationship between various chromosome elements and conversion tracts (Tables S9 and S10) is given in Text S1.

Although dimer formation has a simple relationship to DNA sequence, the rate of NER-mediated repair of the dimers is enhanced by transcription and reduced by chromatin silencing and other aspects of chromatin structure [45]–[47]. Our discussion of dimer repair will be limited to NER, since our experiments were done under conditions in which photoreactivation was prevented. In general, dimer repair is rapid in yeast with the majority of dimers being removed within two hours [48]. Our observation that UV treatment of G1-synchronized cells primarily results in recombination in the first cell cycle following radiation is consistent with efficient dimer repair. Nonetheless, Teng *et al.* (2011) [44] found genomic regions in which dimer repair was delayed. To test whether these long-lasting lesions could be more recombinogenic than lesions that were quickly repaired, we determined whether the chromosome regions containing the long-lasting lesions were over-represented in our unselected gene conversion tracts (details of the analysis in Text S1). There was not a significant enrichment of the regions with long-lasting lesions in our unselected gene conversion tracts.

### UV-induced BIR events

Most previous studies of BIR involve transforming linear fragments of DNA or using strains in which the interacting homologous sequences are flanked by non-homologous regions [4], [49]. In contrast, our ability to distinguish BIR from crossovers is based on the recovery of both cells containing recombinant products. Among unselected LOH events examined in G1-synchronized cells irradiated with 15 J/m^2^, we observed 60 crossovers and 21 BIR events. By the microarray analysis, as described previously, we detect only half of crossovers. All BIR events, however, can be detected. We conclude, therefore, that crossovers are induced about six-fold more than BIR events. This conclusion is in agreement with previous observations of spontaneous recombination events [50], and events induced by the I-SceI endonuclease [51] performed by others.

About 60% of the UV-induced BIR events appear to be randomly distributed, whereas the remainder have a breakpoint located within 50 kb of the telomere. We suggest that there are two types of BIR events, the “classic” type in which one of the chromosome fragments is lost prior to second end capture, and a second type that is initiated by degradation of one of the two homologs beginning at the telomere. In two previous studies [52], [53], LOH events near the telomere were observed in strains with mutations in genes affecting telomere structure or replication (*CDC13* and *EST1*). It was not determined in these studies whether these LOH events were crossovers or BIR events. Since the BIR events in our study were induced by UV, one interpretation of our results is that high doses of UV are associated with telomere uncapping or some other telomere defect. An alternative explanation of our observation that BIR events are enriched near the telomere is that such events are more efficiently initiated and completed than events located more internally on the chromosome, as demonstrated by Donnianni and Symington [49].

### Association between conversion and crossovers

In meiosis in *S. cerevisiae*, roughly half of the conversion events are associated with crossovers [1], [54]. The fraction of conversions associated with crossovers varies in different studies from <5% to 50% [4]. In the unselected events induced by 15 J/m^2^, we observed 300 conversions without an observable crossover, and 60 crossovers. Because of the pattern of chromosome segregation, we expect only half of the crossovers will lead to LOH distal to the exchange. We calculate that there are likely 240 conversions unassociated with crossovers and 120 associated with crossovers. Thus, we conclude that about one-third of the unselected conversion events are associated with a crossover, similar to our previous conclusion based on a smaller number of events [7].

### Lack of UV-induced chromosome alterations

In our previous analysis of gamma ray-treated G2-synchronized diploids, we observed about two unselected chromosome aberrations per irradiated cell [31]. We found that most of these events reflected homologous recombination between Ty elements located at non-allelic positions. In our current study, although UV treatment induced about eight unselected LOH events per cell irradiated with 15 J/m^2^, we did not detect any large deletions, duplications, or translocations. The difference in these two studies is likely to reflect the total number of DSBs and other recombinogenic lesions generated by the two treatments. In the Argueso *et al.* study, the gamma ray dose (800 Gray) produces about 250 DSBs/cell. Based on the estimate that 40 J/m^2^ of UV results in 5–10 DSBs in G2-synchronized cells [42], we expect only about two DSBs/cell as the result of irradiating G1-synchronized cells with 15 J/m^2^. Since Ty elements, the main target for chromosome rearrangements, represents only a small fraction of the genome (2%), the likelihood of a UV-induced DSB within Ty elements is small, although DSBs located near Ty elements can also contribute to Ty-Ty exchanges [55]. Of the 360 unselected conversion and crossover events induced by UV, 14 included a Ty element; it is unclear whether these events initiated within or nearby the Ty. Although the lack of recombinogenic lesions within or near Ty elements may be sufficient to explain the dearth of chromosome rearrangements, other factors may also be important, since UV does not effectively stimulate recombination between non-allelic Ty elements [56], [57].

## Materials and Methods

### Yeast strains

Most of our experiments were done with the diploid strain PG311, a hybrid that is heterozygous for about 52,000 SNPs [2]. The PG311 genotype is: *MAT* **a**/*MATα::NATade2-1/ade2-1 can1-100/can1Δ*::*SUP4-o ura3-1/URA3 trp1-1/TRP1 his3-11,15/HIS3 leu2-3,112/LEU2 V9229::HYG/V9229 V261553::LEU2/V261553 GAL2/gal2 RAD5/RAD5*. Additional features of this strain are described in Text S1 (Supplemental Materials and Methods). We also describe the strains JSC24,JSC25 and PSL101, all of which are isogenic to PG311 except for the specified alterations. The experiments to measure recombination within the ribosomal RNA genes were done with the diploid AMC45 that is heterozygous for markers flanking the array and within the array [34]. The diploid YYy13 is a *MATα::NAT* derivative of AMC45. Unlike the other strains used in our analysis, AMY45 and YYy13 are not isogenic with PG311.

### Media and genetic methods

Standard rich growth medium (YPD) and omission media were used for these experiments [58]. We also used standard conditions for tetrad analysis, transformation, and DNA isolation.

### Cell synchronization and UV treatment

In most of our experiments, PG311 cells were synchronized in G1 using α-factor or in G2 using nocodazole as described by Lee and Petes [11]. After two hours of treatment with these agents, the cells were plated on medium lacking arginine and irradiated with UV using a TL-2000 UV Translinker; doses varied between 1 and 15 J/m^2^. Following the UV treatment, the plates were covered with foil to prevent light-associated removal of dimers, and incubated for two days to allow the formation of sectored colonies. In some experiments, modifications of this protocol were employed as described in Supplemental Materials and Methods.

### SNP microarray analysis

LOH events in PG311 and related strains were detected using SNP microarrays. For each SNP, these Agilent-constructed microarrays contain four oligonucleotides, one pair that hybridizes to the YJM789-derived SNP allele and another that hybridizes to the W303a-derived SNP allele [7]. About 13,000 SNPs distributed throughout the genome were examined. A short description of the use of SNP microarrays is in the Results section and additional details are given in St. Charles *et al.* (2012) [7]. In brief, genomic DNA from the experimental strain was labeled with Cy5-dUTP and control DNA from the fully heterozygous strain JSC24-2 was labeled with Cy3-dUTP. The two DNA samples were then hybridized in competition to the SNP microarrays. The microarray was examined using a GenePix scanner. By measuring the ratio of hybridization of the two differentially-labeled samples, we could determine which SNPs were heterozygous and which were homozygous.

### Statistical analyses

Most of our statistical analysis involved chi-square analysis, the Fisher exact test, or the Mann-Whitney test. These tests were done using the VassarStat Website (http://vassarstats.net/) or the functions associated with Excel. To calculate 95% confidence limits on the median, we used Table B11 of Altman (1990) [59].

## Supporting Information

Figure S1 Recombination and/or chromosome loss events leading to different types of sectored colonies. Red and white lines depict the different homologs with circles showing centromeres and triangles showing the location of the *SUP4-o* insertion. The diploid is homozygous for the *ade2-1* mutation and strains with zero, one, and two copies of *SUP4-o* produce red, pink, and white colonies respectively. A. Reciprocal crossover resulting in a red/white sectored colony. B. Chromosome loss at the first division producing a pink/red sectored colony. C. Reciprocal crossover in the first cell cycle, followed by chromosome loss in one of the daughters, producing a pink/white/red sectored colony. D. Reciprocal crossover in the second division resulting in a pink/white/red sectored colony.
(TIF)

Figure S2 LOH patterns in sectors derived from a pink/white/red sectored colony (78PWR). We isolated purified derivatives from each sector of 78PWR and analyzed their DNA by SNP microarrays. On chromosome II, the samples derived from the white (78W) and red (78R) sectors had identical interstitial LOH events, whereas the sample derived from the pink sector (78P) had an LOH event in which one transition occurred at the same position and the second at a different position from the events in the other two sectors. This pattern is consistent with a DSCB that was repaired in the first division to generate a hybrid 4∶0/3∶1 tract.
(TIF)

Figure S3 Patterns of marker segregation reflecting BIR events on the right arm of chromosome XII. The diploid strain YYy13 is heterozygous for a gene affecting resistance to hygromycin (*HYG*), *TRP1*, and *URA3*. G1-synchronized cells on rich growth medium were irradiated with UV. After colonies were formed, they were replica-plated to media containing hygromycin, lacking uracil, lacking tryptophan, or containing 5-fluoro-orotate (5-FOA). On plates containing 5-FOA, strains lacking *URA3* grow confluently and strains homozygous for *URA3* do not grow at all. Strains that are heterozygous for the *URA3* marker form 5-FOA-resistant papillae, reflecting the loss of the *URA3* marker by recombination or chromosome loss in a small fraction of the cells. A. Marker segregation pattern expected as a consequence of a BIR event initiated by a DSB centromere-proximal to the *HYG* marker on the homolog with the *HYG* and *TRP1* markers. B. Marker segregation pattern expected as a consequence of a BIR event initiated by a DSB centromere-proximal to the *HYG* marker on the homolog with the *URA3* marker. C. Marker segregation pattern expected as a consequence of a BIR event initiated by a DSB between the *HYG* and *TRP1* markers on the homolog with the *HYG* and *TRP1* markers. D. Marker segregation pattern expected as a consequence of a BIR event initiated by a DSB between the *HYG* and *TRP1* markers on the homolog with the *URA3* marker. E. Marker segregation pattern expected as a consequence of a BIR event initiated by a DSB between the *TRP1* and *URA3* markers on the homolog with the *HYG* and *TRP1* markers. F. Marker segregation pattern expected as a consequence of a BIR event initiated by a DSB between the *TRP1* and *URA3* markers on the homolog with the *URA3* marker.
(TIF)

Figure S4 Patterns of marker segregation in strains with intrachromosomal recombination in the ribosomal DNA. The strain depicted is YYy13 and the markers are shown in the same way as in Figure S3. A. Intrachromosomal deletion. A DSB occurs near the *TRP1* insertion, and the resulting processing of the broken ends results in loss of *TRP1*. Because the rDNA genes are repeated, the broken ends can reanneal (single-strand annealing). B. Unequal sister-chromatid recombination. A crossover between misaligned rDNA tandem arrays results in loss of the *TRP1* gene from one chromatid and its duplication in another.
(TIF)

Figure S5 Recombination pathway resulting in Class E1. In this figure, we show the chromosomes as double-stranded DNA molecules with terminal arrow indicating the 3′ ends. Red and black lines show sequences from W303a- and YJM789-derived homologs, respectively. Dotted lines indicate DNA synthesis, and blue ovals represent centromeres. The event is initiated by a DSB in G1, resulting in two black chromatids broken at the same place. Chromatid 1 is repaired by SDSA, and Chromatid 2 by formation of a double Holliday junction (processing of the junction indicated by blue triangles). Regions of heteroduplexes (pairing of black and red strands) are shown in blue rectangles; these regions are repaired to generate either two red strands or two black strands before chromosome segregation. The net result of these events is a 3∶1/4∶0/3∶1 conversion tract.
(TIF)

Figure S6 Recombination pathway resulting in Class E3. Symbols are identical to those used for Figure S5. Two sister chromatid breaks are repaired, one by SDSA and one by dissolution of a double Holliday junction (indicated by blue arrows). These events result in a 3∶1/4∶0/3∶1 conversion event similar to that shown in Figure S6. The difference between the two types of conversion tracts is that homozygous regions of the 3∶1 tracts are in the same sector in Class E3 and in different sectors in Class E2.
(TIF)

Figure S7 Patterns of LOH expected as a consequence of sister chromatids with DSBs that are close together, but not at the same position. In this figure, we show conversion tracts that are propagated unidirectionally from the DSB as observed by Mitchel *et al.* (2010) [30]. Assuming that conversion tracts from the two DSBs are propagated independently, four patterns (Figure S7A–S7D) are expected. One-quarter of the events should result in two 3∶1 conversion tracts separated by a region of heterozygous SNPs (Figure S7D).
(TIF)

Figure S8 Recombination pathway resulting in Class F1. The event is initiated by a single DSB on Chromatid 2. Formation of a double Holliday junction results in two regions of heteroduplex. If the double Holliday junction is cleaved symmetrically to form a non-crossover, and the left heteroduplex is corrected to yield two black strands and the right heteroduplex is corrected to yield two red strands, the net result of these events will be a sectored colony with a 3∶1 conversion event and a region (HOM) that is homozygous for the W303a-derived SNPs in one sector and for YJM789-derived SNPs in the other. Note that this pattern of LOH is different from that expected by a single crossover in which the LOH region extends to the end of the chromosome.
(TIF)

Figure S9 Generation of Class G1 by the SDSA pathway. In this figure, we show repair of a single DSB by the SDSA pathway. Mismatches formed in the heteroduplex during strand invasion are repaired. Following dissociation of the invading strand, there are two other regions of heteroduplex (boxed in blue). If the left heteroduplex is corrected to yield two red strands and the right heteroduplex is corrected to generate two black strands, a Class G1 sectored colony can be generated.
(TIF)

Figure S10 Generation of Class G1 by branch migration of a double Holliday junction. A single DSB is repaired by forming a double Holliday junction. Following its formation, branch migration occurs (shown by blue arrow), extending the length of the right-hand heteroduplex. Patchy repair within this heteroduplex produces the observed LOH pattern.
(TIF)

Figure S11 Mechanism to generate Class J1. As discussed in Text S1, the homozygous portion of a 3∶1 conversion tract is usually associated with the sector that is homozygous for SNPs from the same strain in the crossover LOH region. For example, in Class H3, the homozygous red portion of the 3∶1 tract is in the red sector. This pattern is expected for a conversion-associated crossover (Figure S14 of [7]). In Classes J1–J4, however, the conversion event is in the “wrong” sector. This pattern can be explained as a consequence of repair of two DSBs as shown in this figure. In this depiction, we assume that the very small conversion tracts occur between SNPs and are, therefore, not detectable. Similar events have been observed previously (Figure S1 of [2]).
(TIF)

Figure S12 Mechanism to generate Class J5. In this class, a 3∶1 conversion tract is separated from the crossover by a heterozygous region. This event involves the repair of two DSBs associated with the formation of two large regions of heteroduplex. Conversion-type repair of the left heteroduplex is associated with SDSA. The right heteroduplex undergoes restoration-type repair and is associated with a crossover.
(TIF)

Figure S13 Model to explain Class L1 and related sectors initiated with two independent DSBs. For sectors in which transitions between different LOH regions were greater than 15 kb apart, we assume that two independent initiation events are involved. A. Two independent recombination events producing Class L1. In this figure, we show one DSB that is repaired, resulting in a 3∶1 conversion tract associated with a crossover. The second DSB is repaired by forming a double Holliday junction and resolving the intermediate in the non-crossover mode similar to that shown in Figures 1B and S8. Restoration-type repair would result in a red patch within the black homolog and a black patch within the red homolog. B. Depiction of Class L1 showing regions of conversion in each sector.
(TIF)

Figure S14 Generation of Class M1 by BIR. One broken black chromatid initiates a BIR event that copies a red chromatid to the end of the chromosome.
(TIF)

Figure S15 Generation of Class P1 by two independent repair events. One of the broken chromatids is repaired by SDSA and the second is repaired by a BIR event.
(TIF)

Table S1 Conditions for generating the sectors (high UV dose). The UV-induced sectors were generated under experimental conditions that varied slightly (dosage, media, temperature of incubation, and plating method). The conditions under which each sectored colony was obtained are described in this table. ^1^G1-synchronized cells were treated with UV doses of either 5, 10 or 15 J/m^2^. ^2^Before UV treatment, synchronized cells were plated on omission medium lacking arginine with or without canavanine (CAN). ^3^Plates with the treated cells were incubated at either 30°C. or room temperature (R.T., approximately 23°C). ^4^Treated cells were either plated and allowed to form colonies without further manipulation (“plating”) or were plated on a restricted region of the plate and micromanipulated into defined positions before allowing them to form colonies (“single cell”). Other relevant details are in Text S1.
(XLS)

Table S2 Classes of UV-induced LOH events in sectored colonies resulting from treating G1-synchronized cells with a UV dose of 15 J/m^2^. ^1^Related classes of LOH events within sectored colonies were grouped. “SCB” indicates that the class is most readily explained as a consequence of a single-chromatid break, and “DSCB” indicates that the class is most readily explained as reflecting repair of two sister-chromatids broken at the same position. As described in the main text, each sectored colony is represented by a pair of lines; for selected crossovers on chromosome V, the top line shows the pattern in the red sector and the bottom line indicates the pattern in the white sector. Green, red, and black segments represent heterozygous SNPs, SNPs homozygous for the W303a-derived alleles, and SNPs homozygous for the YJM789-derived alleles, respectively. The lengths of the line segments are not proportional to the sizes of the LOH regions. Small letters indicate transitions between heterozygous and homozygous regions. Classes A–G depict gene conversion tracts unassociated with crossovers. Classes H–L are crossover events. Classes M–P are BIR events. Each of the Classes L and P events contains LOH events separated by more than 15 kb. We interpret them as two independently initiated events. The two initiation regions for these two classes are indicated by black boxes or black straight lines. Class Q is a chromosome loss event. See Text S1 for a detailed description of the classes. ^2^The number of events belonging to each class refers to those observed in 15 J/m^2^-treated G1 sectors. In Classes H–L, we include the number of events belonging to each class observed in 1 J/m^2^-treated G1 sectors in the parentheses.
(XLS)

Table S3 SGD coordinates for transitions in sectored colonies induced by a UV dose of 15 J/m^2^ in G1-synchronized cells. For each sectored colony, we show both selected and unselected LOH events. Information about the event class, line number, and transition labels are derived from Table S2. The numbers in the leftmost two columns represent SGD coordinates for SNPs flanking each transition, beginning at the left telomere; these coordinates are based on the values used for the 2009 version.
(XLS)

Table S4 Depiction of UV-induced LOH events in 15 J/m^2^-treated pink/white/red sector (78PWR) from a single-cell experiment. Genomic DNA was isolated from the pink (P), white (W), and red (R) portions of a tri-colored colony, and examined by SNP microarrays. The line depictions of LOH events in each sector use the same code employed in Table S2.
(XLS)

Table S5 SGD coordinates for transitions of UV-induced LOH events in the pink/white/red (78PWR) and pink/red (409PR) sectored colonies. The depictions of each class of event for 78PWR are shown in Table S4. All events had identical transitions in the red and pink sectors of 409PR. Transition coordinates were determined in the same way as in Table S3.
(XLS)

Table S6 Classes of UV-induced selected crossovers in 1 J/m^2^-treated G1 sectors that were not observed in 15 J/m^2^-treated G1 sectors. Most of the classes of events in cells treated with 1 J/m^2^ were the same as for the cells treated with 15 J/m^2^; these classes are shown in parentheses in Table S2. Classes unique to the cells treated with 1 J/m^2^ are shown in this table.
(XLS)

Table S7 SGD coordinates for transitions in UV-induced selected crossovers in 1 J/m^2^-treated G1 sectors. These transitions are for events depicted in Tables S2 and S6. The table has the same structure as Tables S3 and S5.
(XLS)

Table S8 SGD coordinates of the inclusive regions of each chromosome covered by the SNP microarrays and used for analyzing unselected LOH events. On the microarray, we did not include SNPs for repetitive regions. Most of these regions were in sub-telomeric locations. In this table, the first column shows the chromosome number, the second indicates the leftmost SGD coordinate represented by a SNP on the microarray, and the last column shows the rightmost SGD coordinate of the SNP on the microarray. Since selected events were located between the left telomere and centromere of chromosome V (positions 1 to 153229), this region was not used for examining unselected LOH events.
(XLS)

Table S9 Analysis of over- or under-represented SGD-annotated genome elements within unselected UV-induced conversion tracts. In this table, we summarize our analysis of associations of various SGD-annotated chromosome elements within unselected UV-induced conversion tracts. The details of this analysis are discussed in Text S1.
(XLS)

Table S10 Analysis of associations of various DNA motifs, damage-related genome elements, and transcription rates to UV-induced unselected conversion tracts. The details of this analysis and references for the various motifs are in Text S1.
(XLS)

Text S1 Supplemental materials and methods, and discussion.
(DOC)
