# Supplementary figures and images for: Genome-Wide High-Resolution Mapping of UV-Induced Mitotic Recombination Events in Saccharomyces cerevisiae
Source: PLoS Genet. 2013 Oct 31;9(10):e1003894. doi: 10.1371/journal.pgen.1003894 (PMC3814309; doi:10.1371/journal.pgen.1003894)

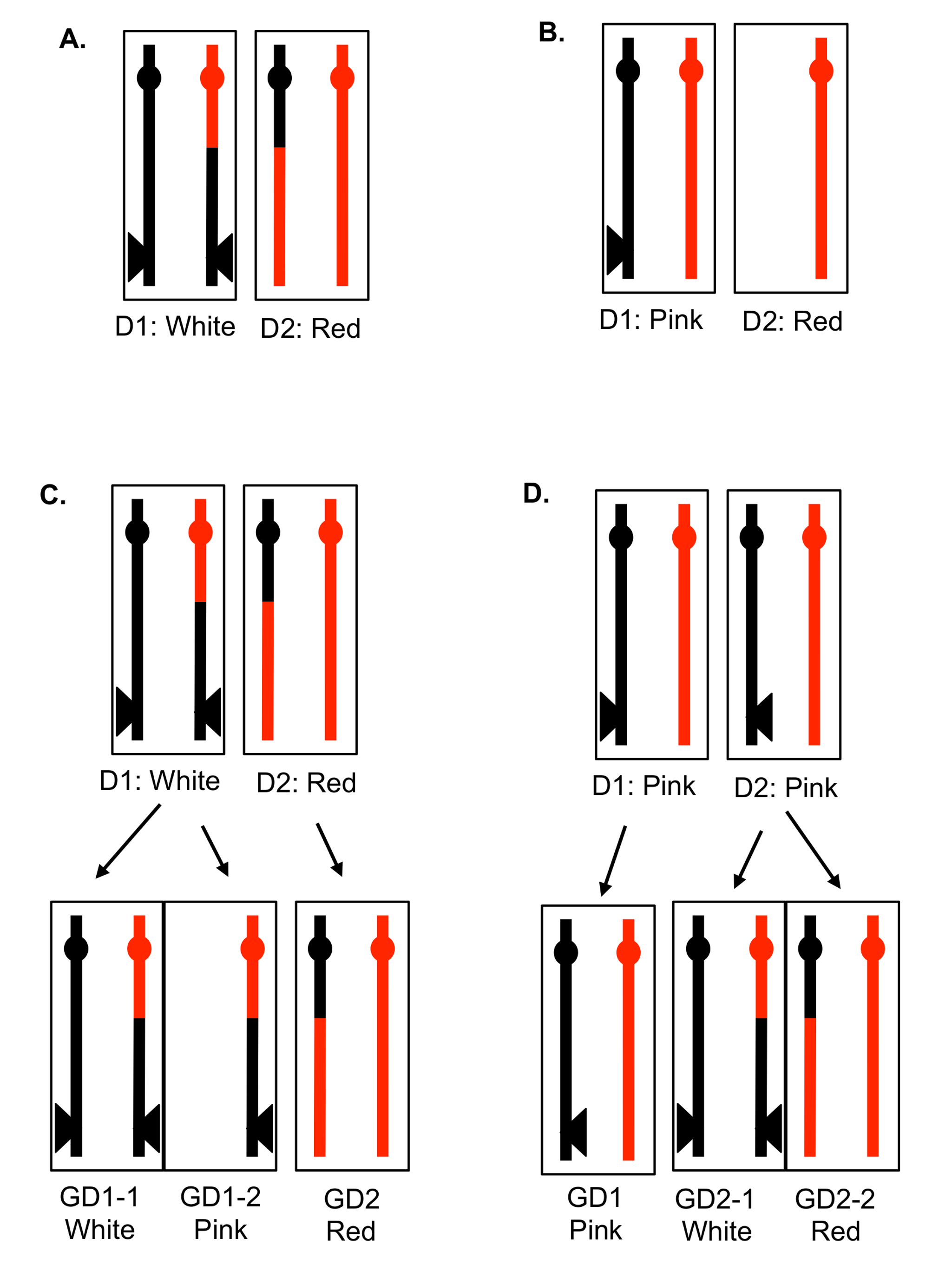

Supplement: Figure S1 — Recombination and/or chromosome loss events leading to different types of sectored colonies. Red and white lines depict the different homologs with circles showing centromeres and triangles showing the location of the SUP4-o insertion. The diploid is homozygous for the ade2-1 mutation and strains with zero, one, and two copies of SUP4-o produce red, pink, and white colonies respectively. A. Reciprocal crossover resulting in a red/white sectored colony. B. Chromosome loss at the first division producing a pink/red sectored colony. C. Reciprocal crossover in the first cell cycle, followed by chromosome loss in one of the daughters, producing a pink/white/red sectored colony. D. Reciprocal crossover in the second division resulting in a pink/white/red sectored colony. (TIF) [file pgen.1003894.s001.tif]

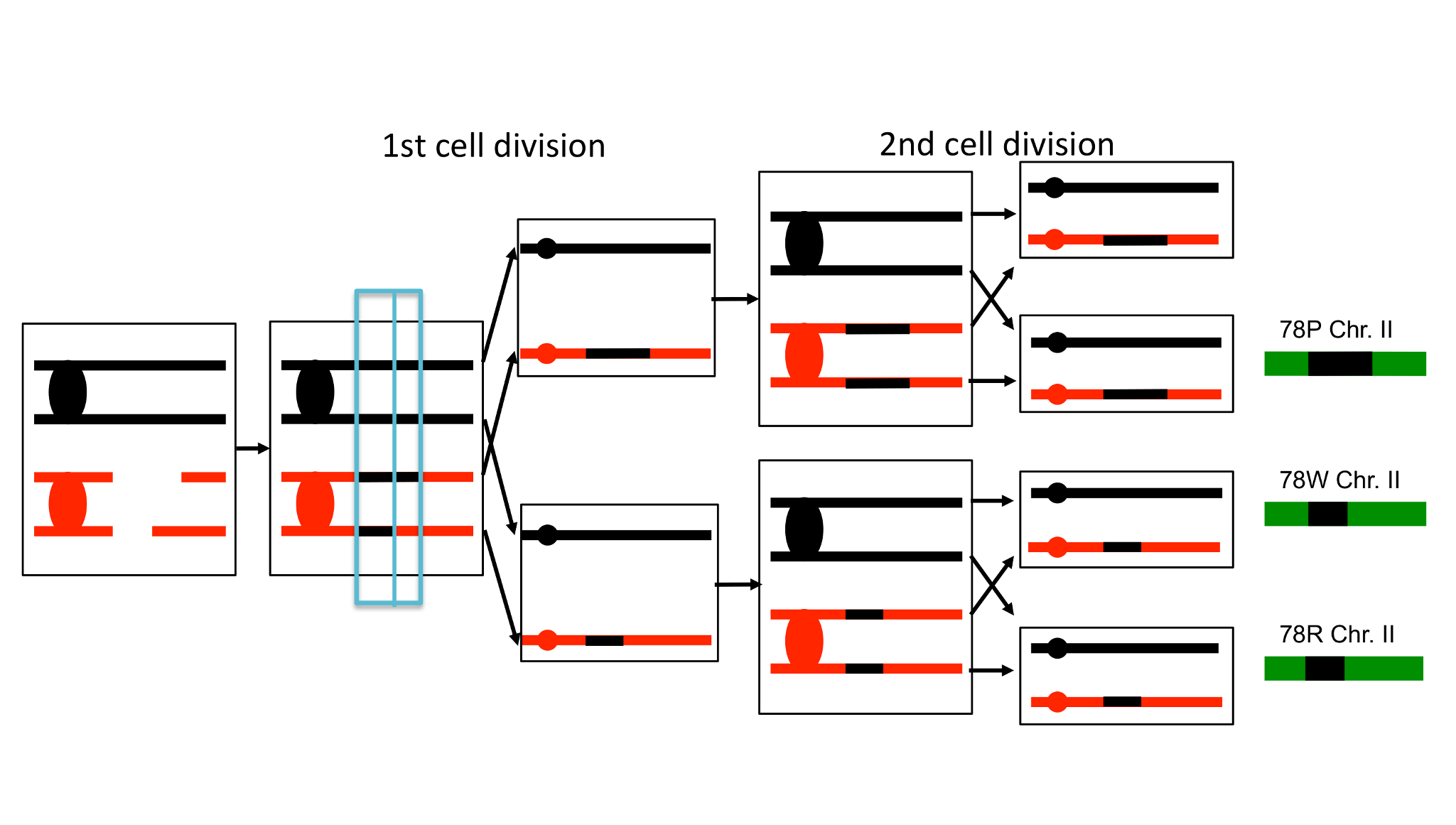

Supplement: Figure S2 — LOH patterns in sectors derived from a pink/white/red sectored colony (78PWR). We isolated purified derivatives from each sector of 78PWR and analyzed their DNA by SNP microarrays. On chromosome II, the samples derived from the white (78W) and red (78R) sectors had identical interstitial LOH events, whereas the sample derived from the pink sector (78P) had an LOH event in which one transition occurred at the same position and the second at a different position from the events in the other two sectors. This pattern is consistent with a DSCB that was repaired in the first division to generate a hybrid 4∶0/3∶1 tract. (TIF) [file pgen.1003894.s002.tif]

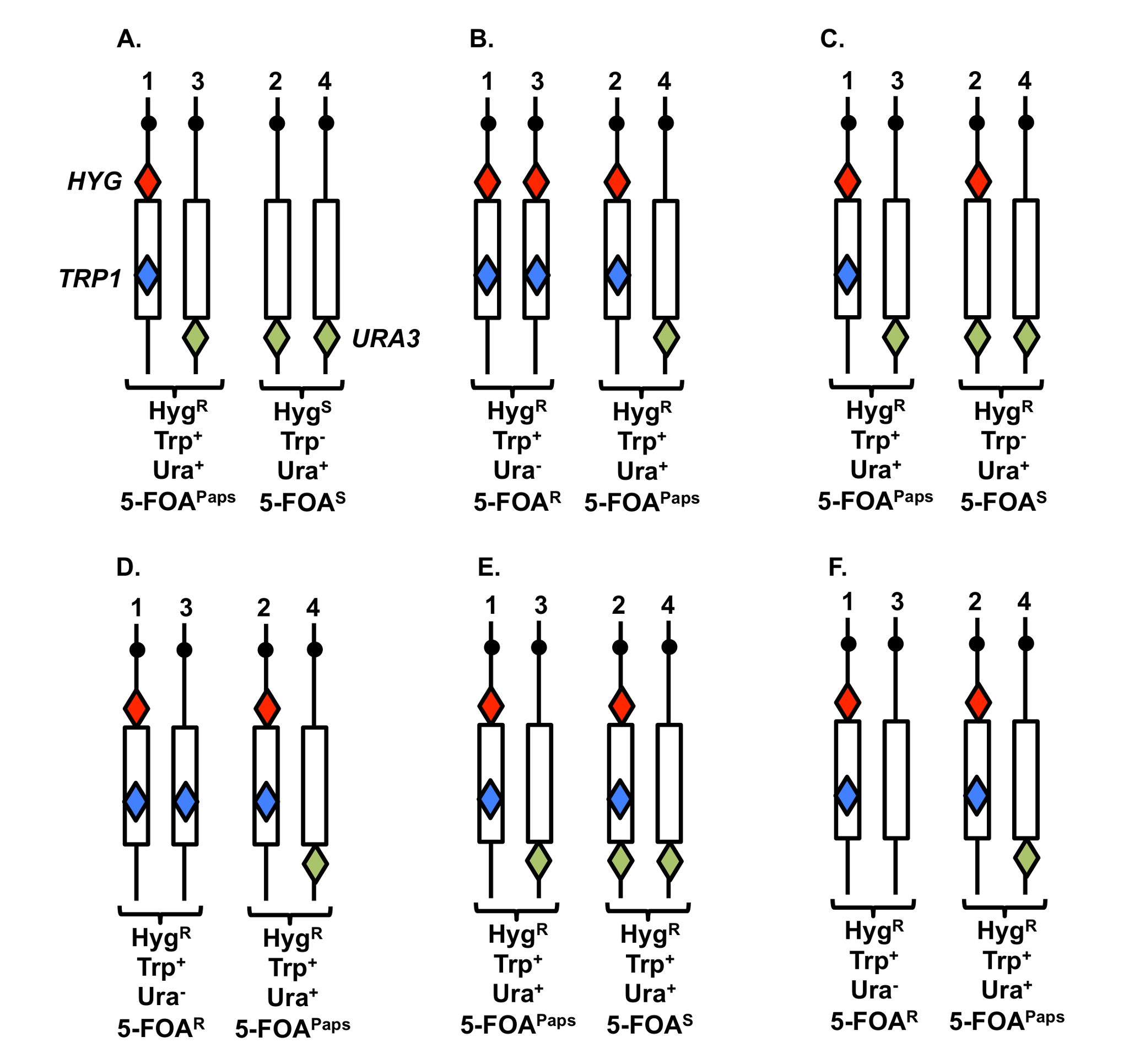

Supplement: Figure S3 — Patterns of marker segregation reflecting BIR events on the right arm of chromosome XII. The diploid strain YYy13 is heterozygous for a gene affecting resistance to hygromycin (HYG), TRP1, and URA3. G1-synchronized cells on rich growth medium were irradiated with UV. After colonies were formed, they were replica-plated to media containing hygromycin, lacking uracil, lacking tryptophan, or containing 5-fluoro-orotate (5-FOA). On plates containing 5-FOA, strains lacking URA3 grow confluently and strains homozygous for URA3 do not grow at all. Strains that are heterozygous for the URA3 marker form 5-FOA-resistant papillae, reflecting the loss of the URA3 marker by recombination or chromosome loss in a small fraction of the cells. A. Marker segregation pattern expected as a consequence of a BIR event initiated by a DSB centromere-proximal to the HYG marker on the homolog with the HYG and TRP1 markers. B. Marker segregation pattern expected as a consequence of a BIR event initiated by a DSB centromere-proximal to the HYG marker on the homolog with the URA3 marker. C. Marker segregation pattern expected as a consequence of a BIR event initiated by a DSB between the HYG and TRP1 markers on the homolog with the HYG and TRP1 markers. D. Marker segregation pattern expected as a consequence of a BIR event initiated by a DSB between the HYG and TRP1 markers on the homolog with the URA3 marker. E. Marker segregation pattern expected as a consequence of a BIR event initiated by a DSB between the TRP1 and URA3 markers on the homolog with the HYG and TRP1 markers. F. Marker segregation pattern expected as a consequence of a BIR event initiated by a DSB between the TRP1 and URA3 markers on the homolog with the URA3 marker. (TIF) [file pgen.1003894.s003.tif]

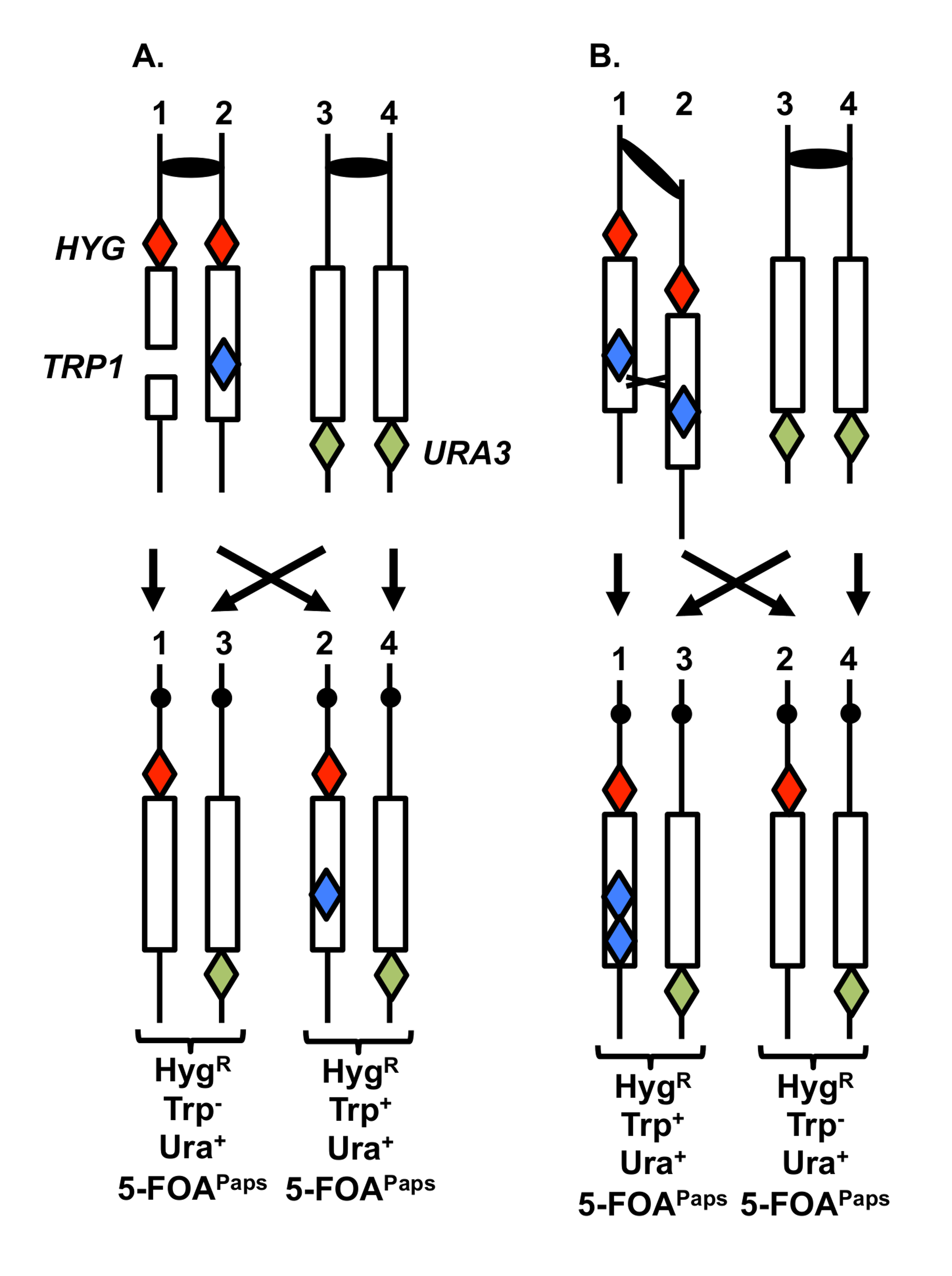

Supplement: Figure S4 — Patterns of marker segregation in strains with intrachromosomal recombination in the ribosomal DNA. The strain depicted is YYy13 and the markers are shown in the same way as in Figure S3. A. Intrachromosomal deletion. A DSB occurs near the TRP1 insertion, and the resulting processing of the broken ends results in loss of TRP1. Because the rDNA genes are repeated, the broken ends can reanneal (single-strand annealing). B. Unequal sister-chromatid recombination. A crossover between misaligned rDNA tandem arrays results in loss of the TRP1 gene from one chromatid and its duplication in another. (TIF) [file pgen.1003894.s004.tif]

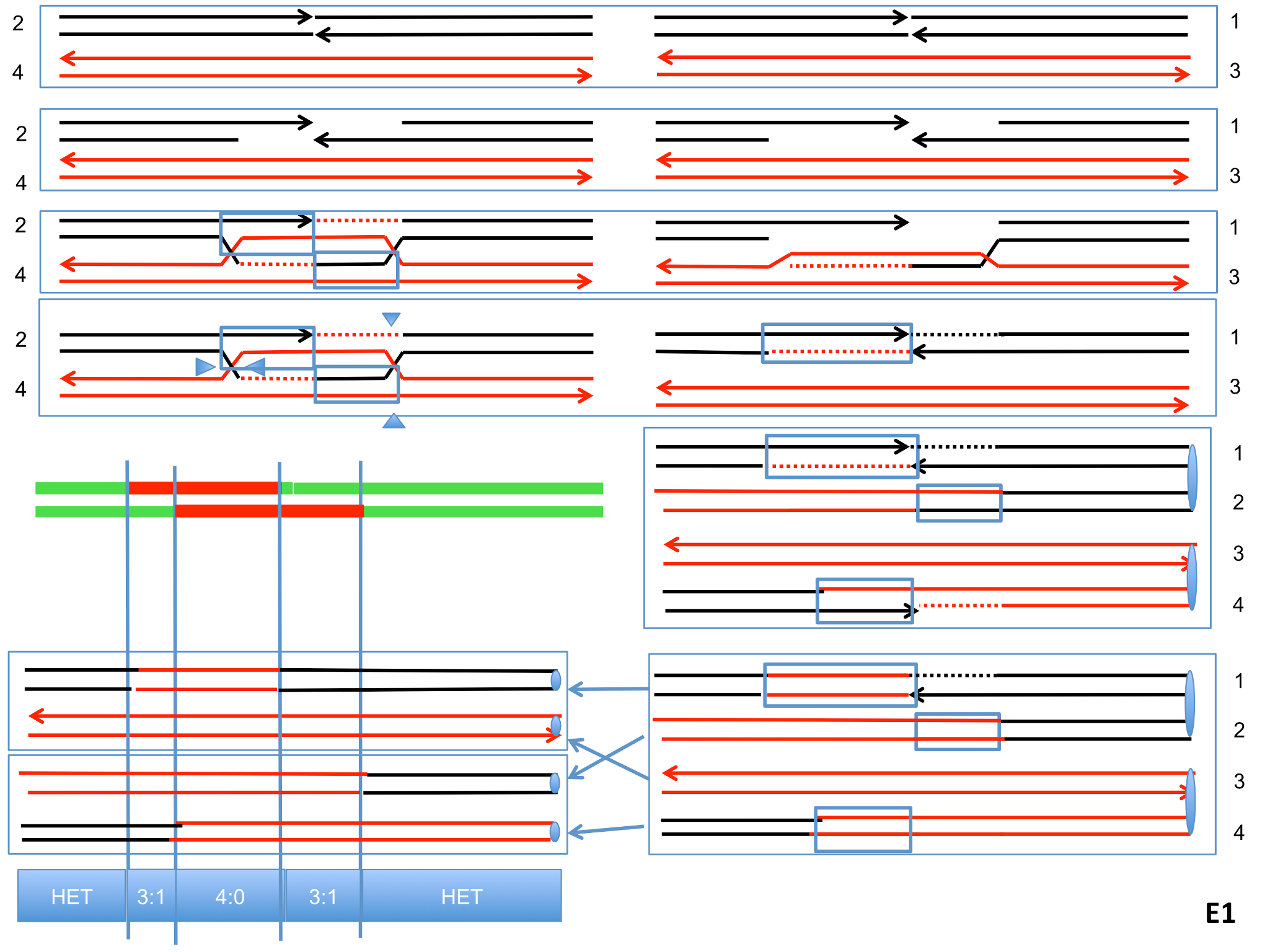

Supplement: Figure S5 — Recombination pathway resulting in Class E1. In this figure, we show the chromosomes as double-stranded DNA molecules with terminal arrow indicating the 3′ ends. Red and black lines show sequences from W303a- and YJM789-derived homologs, respectively. Dotted lines indicate DNA synthesis, and blue ovals represent centromeres. The event is initiated by a DSB in G1, resulting in two black chromatids broken at the same place. Chromatid 1 is repaired by SDSA, and Chromatid 2 by formation of a double Holliday junction (processing of the junction indicated by blue triangles). Regions of heteroduplexes (pairing of black and red strands) are shown in blue rectangles; these regions are repaired to generate either two red strands or two black strands before chromosome segregation. The net result of these events is a 3∶1/4∶0/3∶1 conversion tract. (TIF) [file pgen.1003894.s005.tif]

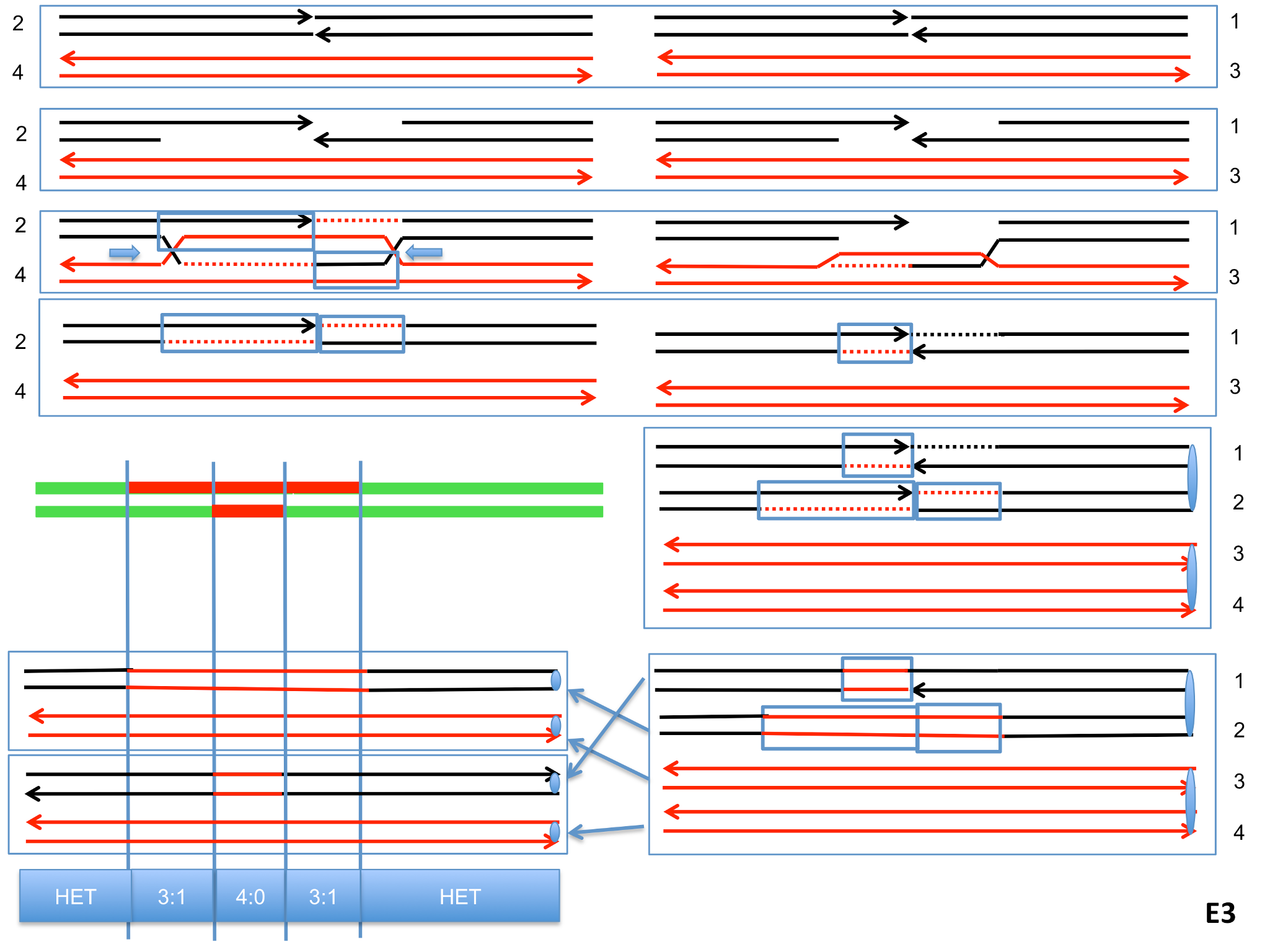

Supplement: Figure S6 — Recombination pathway resulting in Class E3. Symbols are identical to those used for Figure S5. Two sister chromatid breaks are repaired, one by SDSA and one by dissolution of a double Holliday junction (indicated by blue arrows). These events result in a 3∶1/4∶0/3∶1 conversion event similar to that shown in Figure S6. The difference between the two types of conversion tracts is that homozygous regions of the 3∶1 tracts are in the same sector in Class E3 and in different sectors in Class E2. (TIF) [file pgen.1003894.s006.tif]

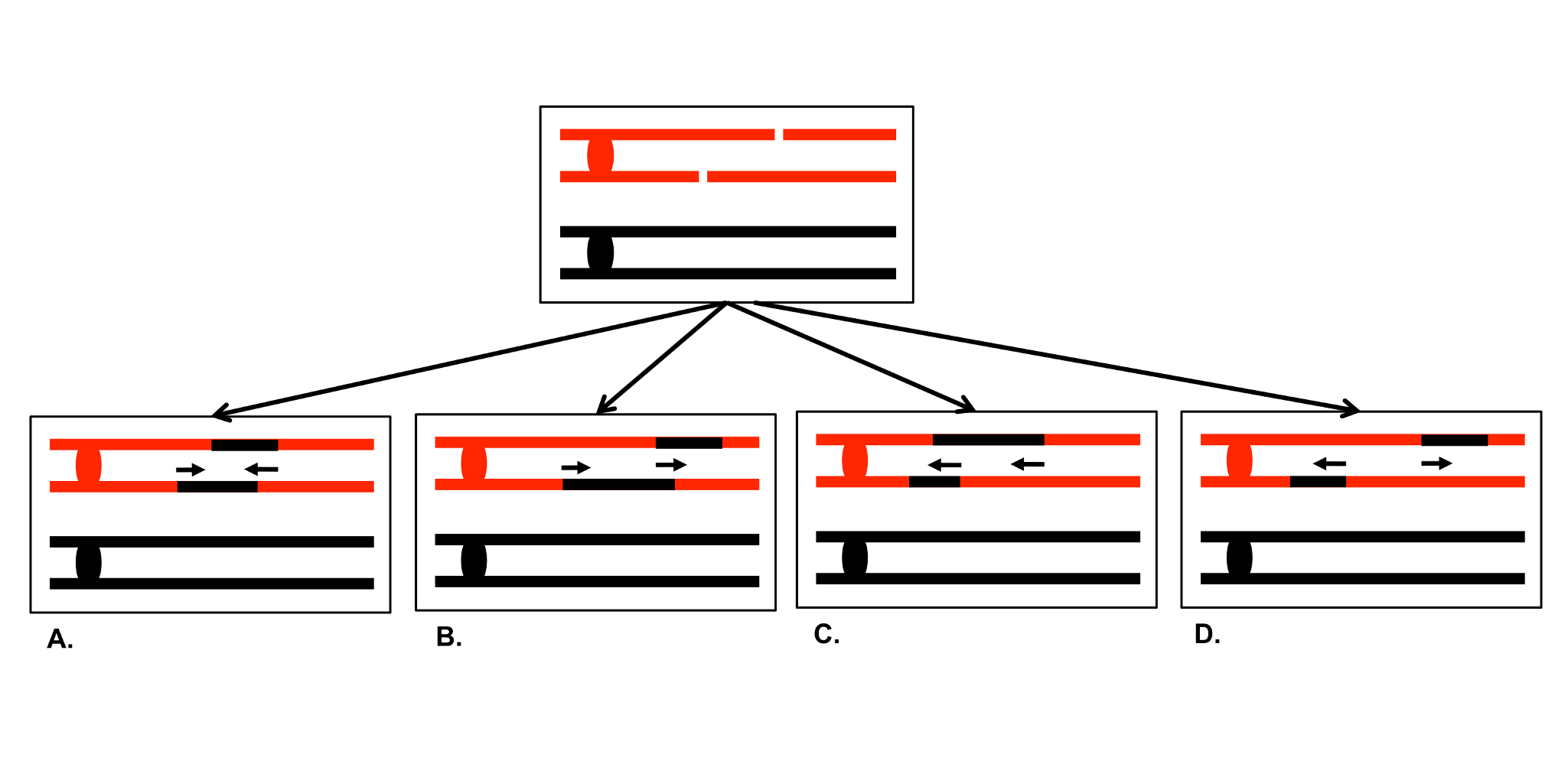

Supplement: Figure S7 — Patterns of LOH expected as a consequence of sister chromatids with DSBs that are close together, but not at the same position. In this figure, we show conversion tracts that are propagated unidirectionally from the DSB as observed by Mitchel et al. (2010) [30]. Assuming that conversion tracts from the two DSBs are propagated independently, four patterns (Figure S7A–S7D) are expected. One-quarter of the events should result in two 3∶1 conversion tracts separated by a region of heterozygous SNPs (Figure S7D). (TIF) [file pgen.1003894.s007.tif]

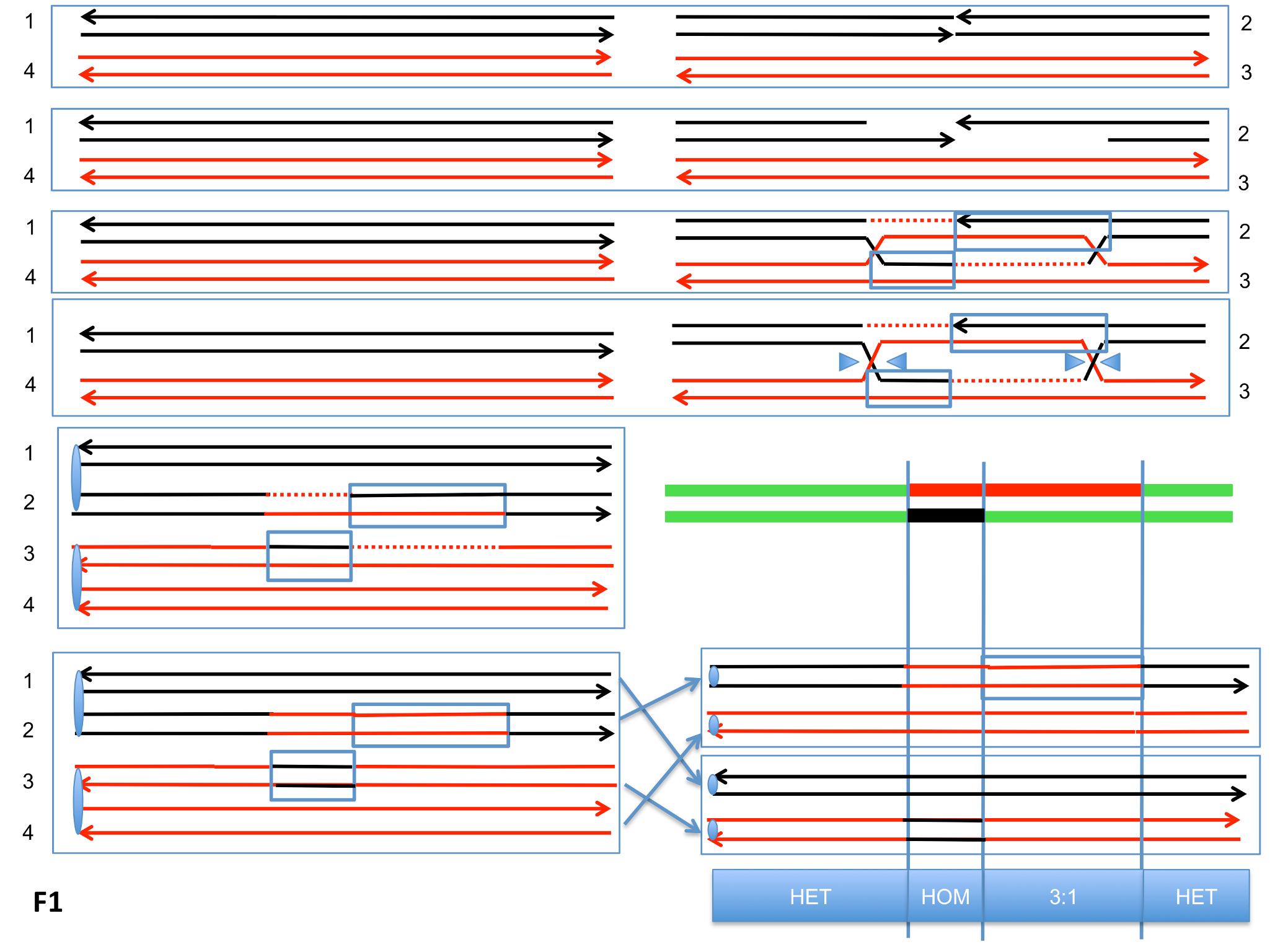

Supplement: Figure S8 — Recombination pathway resulting in Class F1. The event is initiated by a single DSB on Chromatid 2. Formation of a double Holliday junction results in two regions of heteroduplex. If the double Holliday junction is cleaved symmetrically to form a non-crossover, and the left heteroduplex is corrected to yield two black strands and the right heteroduplex is corrected to yield two red strands, the net result of these events will be a sectored colony with a 3∶1 conversion event and a region (HOM) that is homozygous for the W303a-derived SNPs in one sector and for YJM789-derived SNPs in the other. Note that this pattern of LOH is different from that expected by a single crossover in which the LOH region extends to the end of the chromosome. (TIF) [file pgen.1003894.s008.tif]

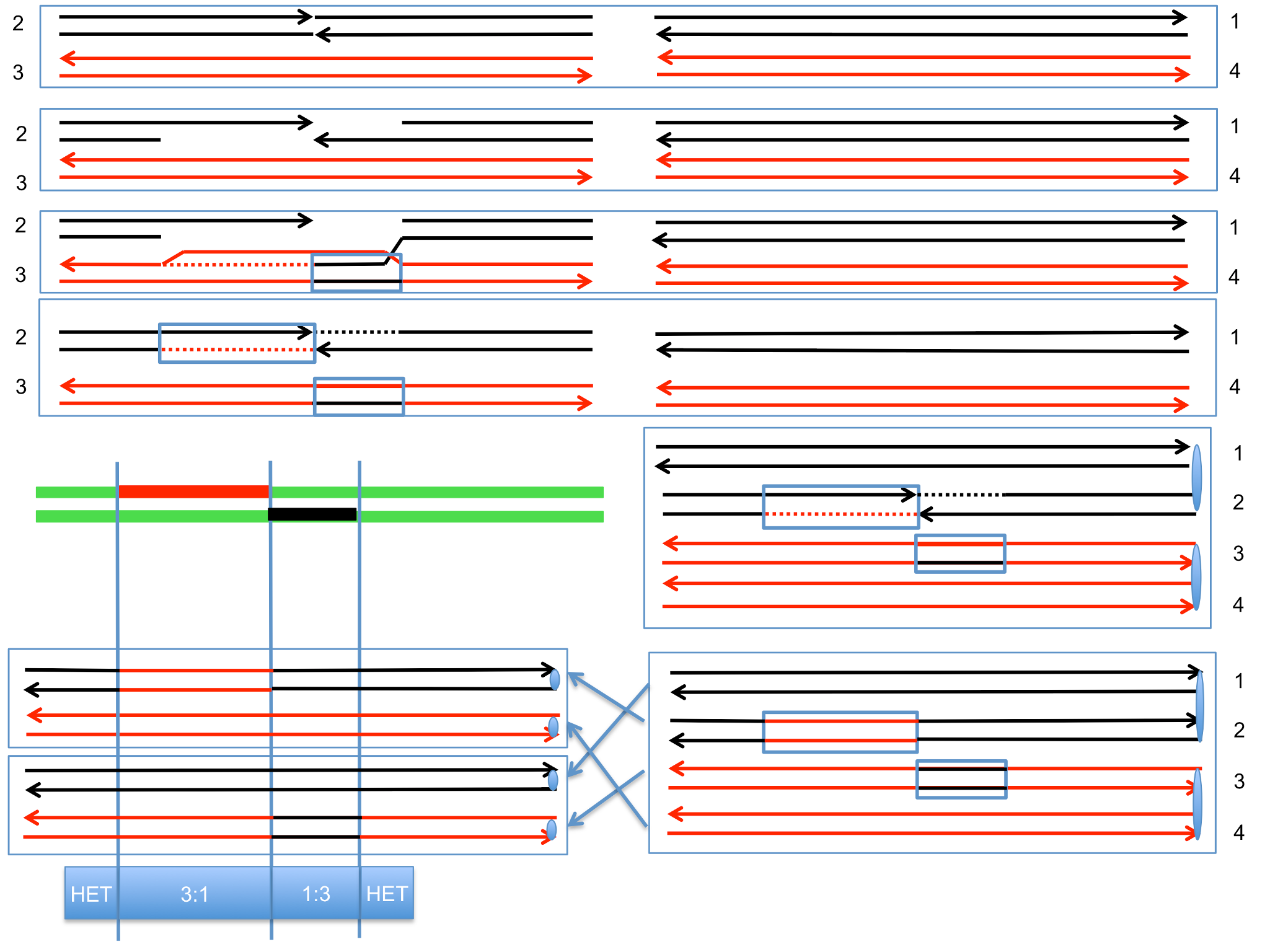

Supplement: Figure S9 — Generation of Class G1 by the SDSA pathway. In this figure, we show repair of a single DSB by the SDSA pathway. Mismatches formed in the heteroduplex during strand invasion are repaired. Following dissociation of the invading strand, there are two other regions of heteroduplex (boxed in blue). If the left heteroduplex is corrected to yield two red strands and the right heteroduplex is corrected to generate two black strands, a Class G1 sectored colony can be generated. (TIF) [file pgen.1003894.s009.tif]

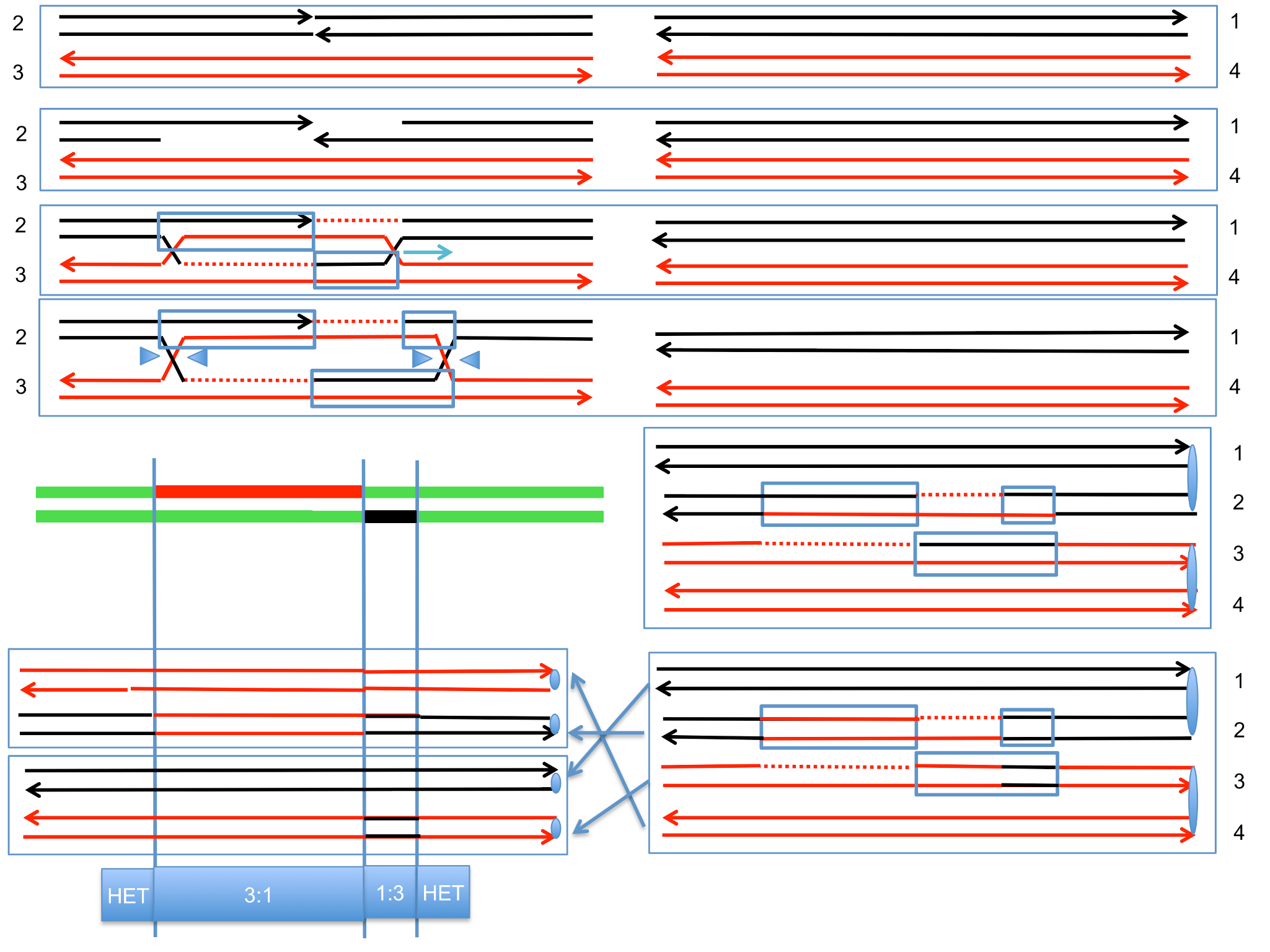

Supplement: Figure S10 — Generation of Class G1 by branch migration of a double Holliday junction. A single DSB is repaired by forming a double Holliday junction. Following its formation, branch migration occurs (shown by blue arrow), extending the length of the right-hand heteroduplex. Patchy repair within this heteroduplex produces the observed LOH pattern. (TIF) [file pgen.1003894.s010.tif]

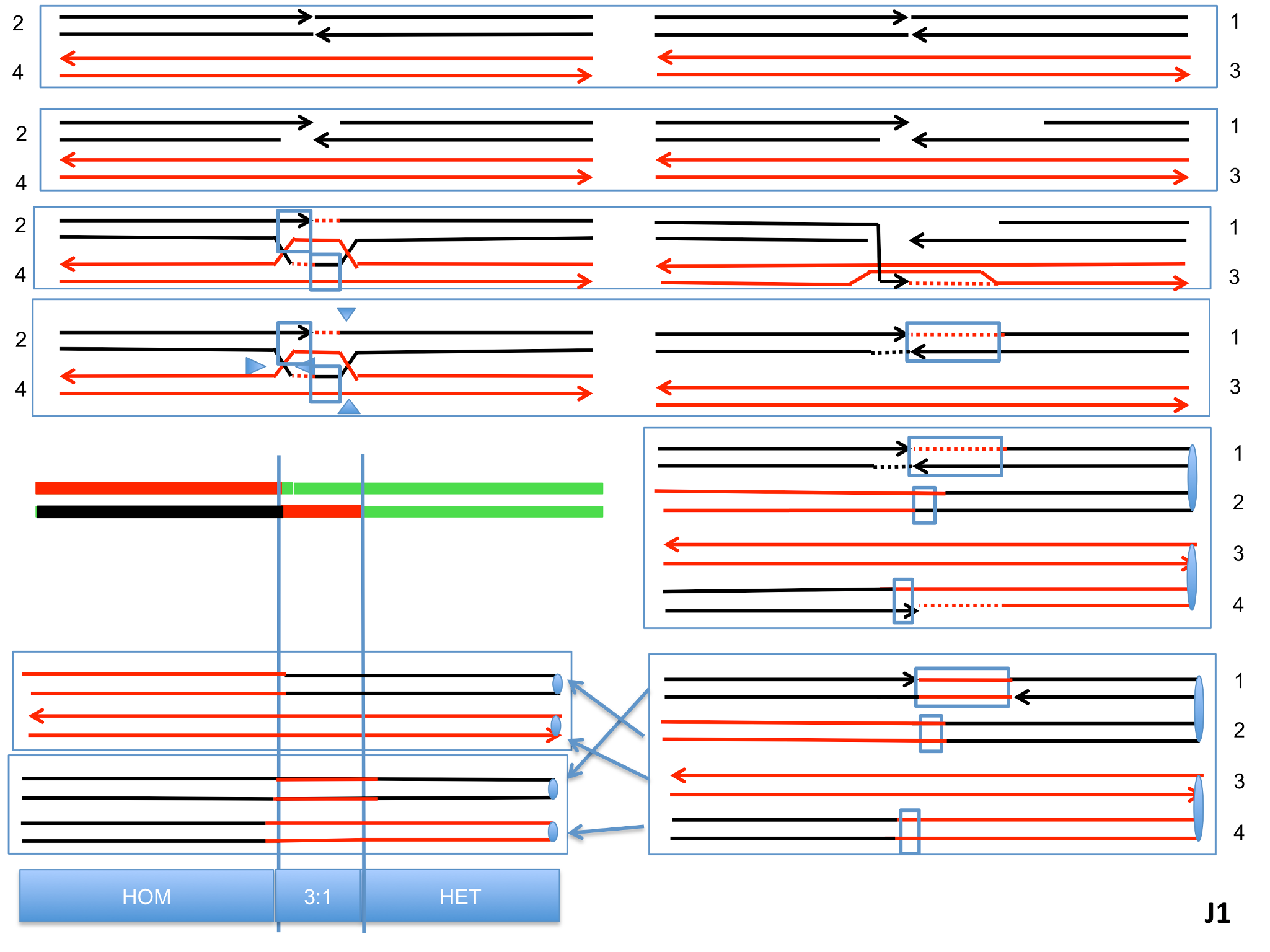

Supplement: Figure S11 — Mechanism to generate Class J1. As discussed in Text S1, the homozygous portion of a 3∶1 conversion tract is usually associated with the sector that is homozygous for SNPs from the same strain in the crossover LOH region. For example, in Class H3, the homozygous red portion of the 3∶1 tract is in the red sector. This pattern is expected for a conversion-associated crossover (Figure S14 of [7]). In Classes J1–J4, however, the conversion event is in the “wrong” sector. This pattern can be explained as a consequence of repair of two DSBs as shown in this figure. In this depiction, we assume that the very small conversion tracts occur between SNPs and are, therefore, not detectable. Similar events have been observed previously (Figure S1 of [2]). (TIF) [file pgen.1003894.s011.tif]

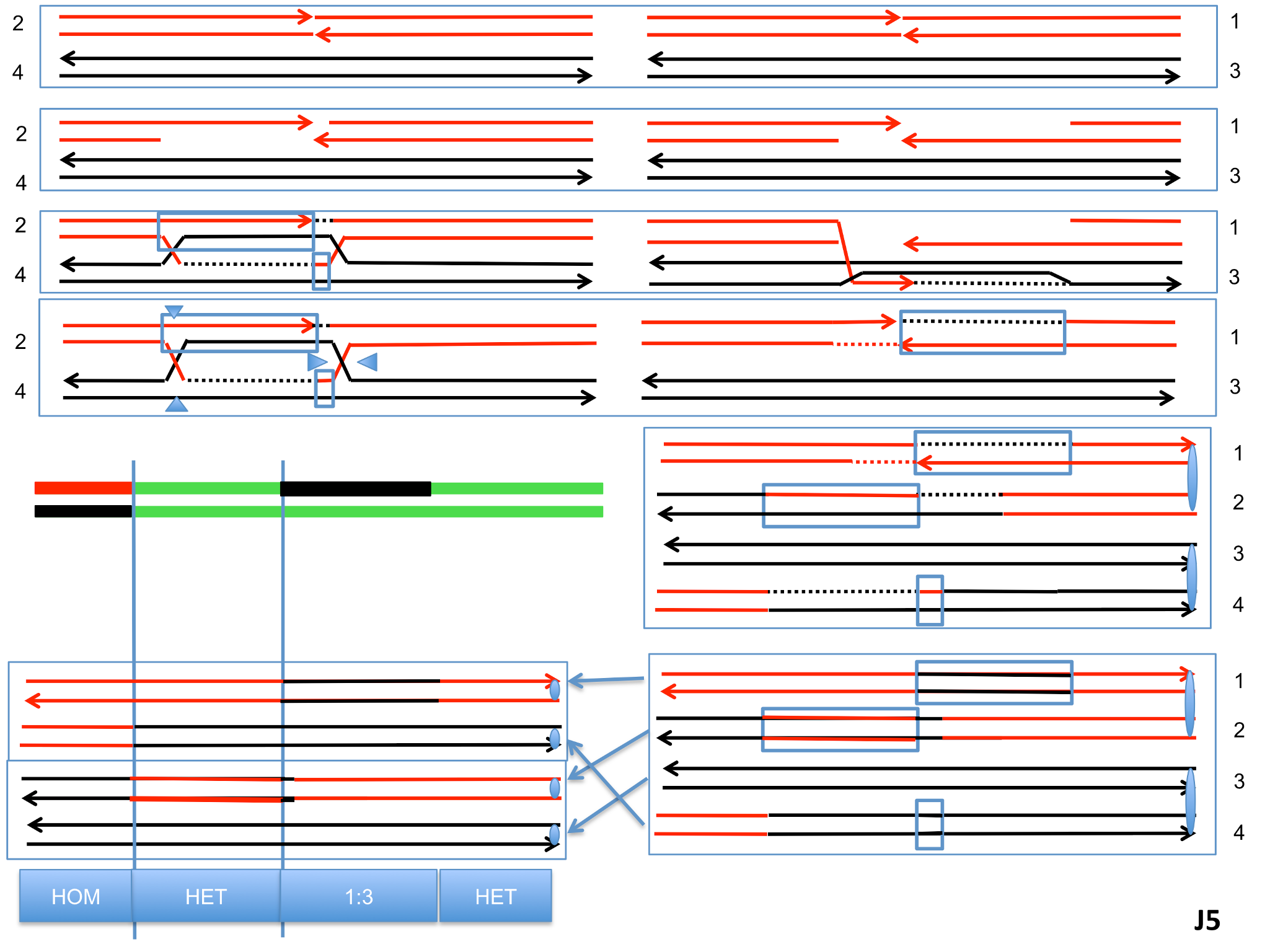

Supplement: Figure S12 — Mechanism to generate Class J5. In this class, a 3∶1 conversion tract is separated from the crossover by a heterozygous region. This event involves the repair of two DSBs associated with the formation of two large regions of heteroduplex. Conversion-type repair of the left heteroduplex is associated with SDSA. The right heteroduplex undergoes restoration-type repair and is associated with a crossover. (TIF) [file pgen.1003894.s012.tif]

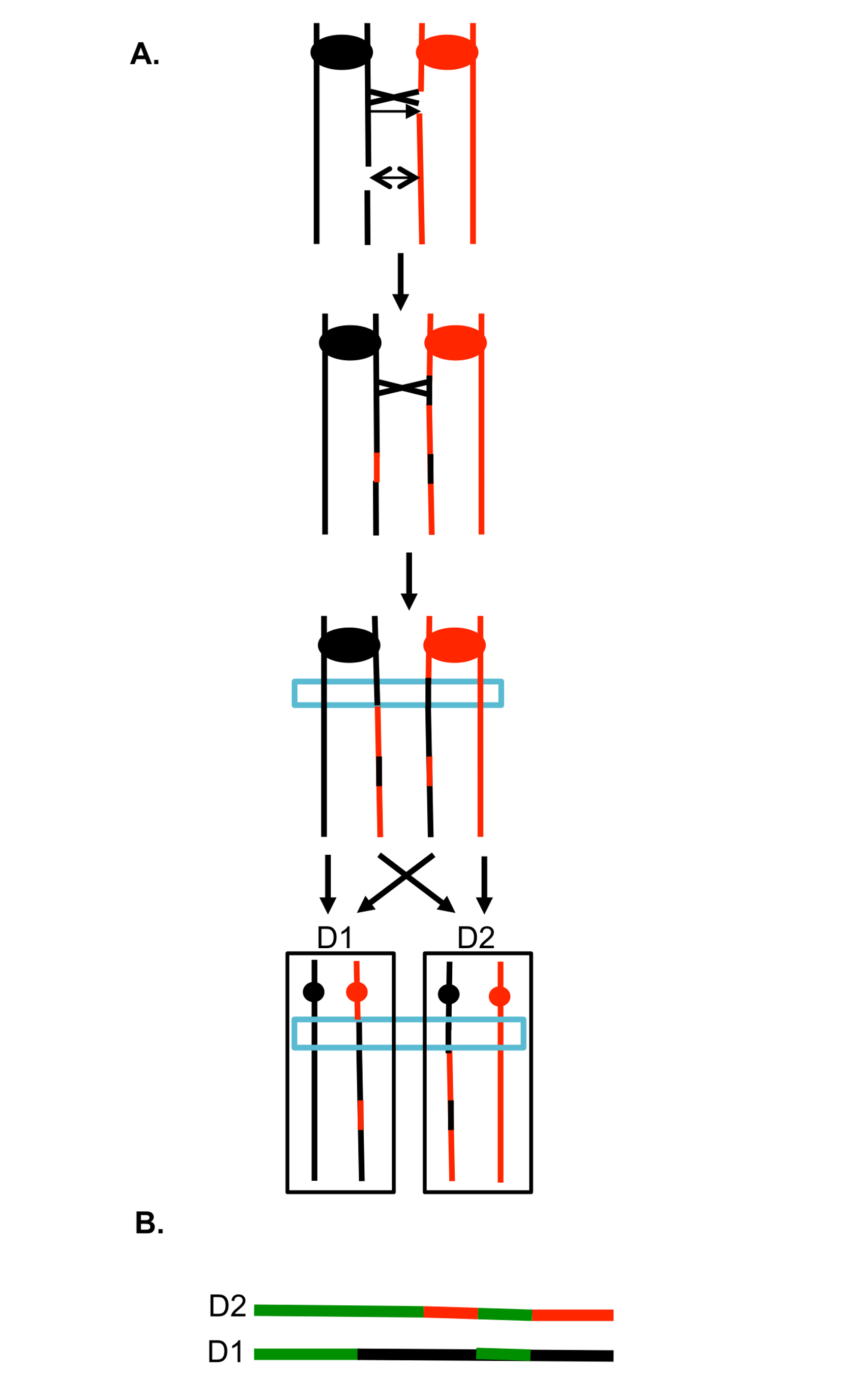

Supplement: Figure S13 — Model to explain Class L1 and related sectors initiated with two independent DSBs. For sectors in which transitions between different LOH regions were greater than 15 kb apart, we assume that two independent initiation events are involved. A. Two independent recombination events producing Class L1. In this figure, we show one DSB that is repaired, resulting in a 3∶1 conversion tract associated with a crossover. The second DSB is repaired by forming a double Holliday junction and resolving the intermediate in the non-crossover mode similar to that shown in Figures 1B and S8. Restoration-type repair would result in a red patch within the black homolog and a black patch within the red homolog. B. Depiction of Class L1 showing regions of conversion in each sector. (TIF) [file pgen.1003894.s013.tif]

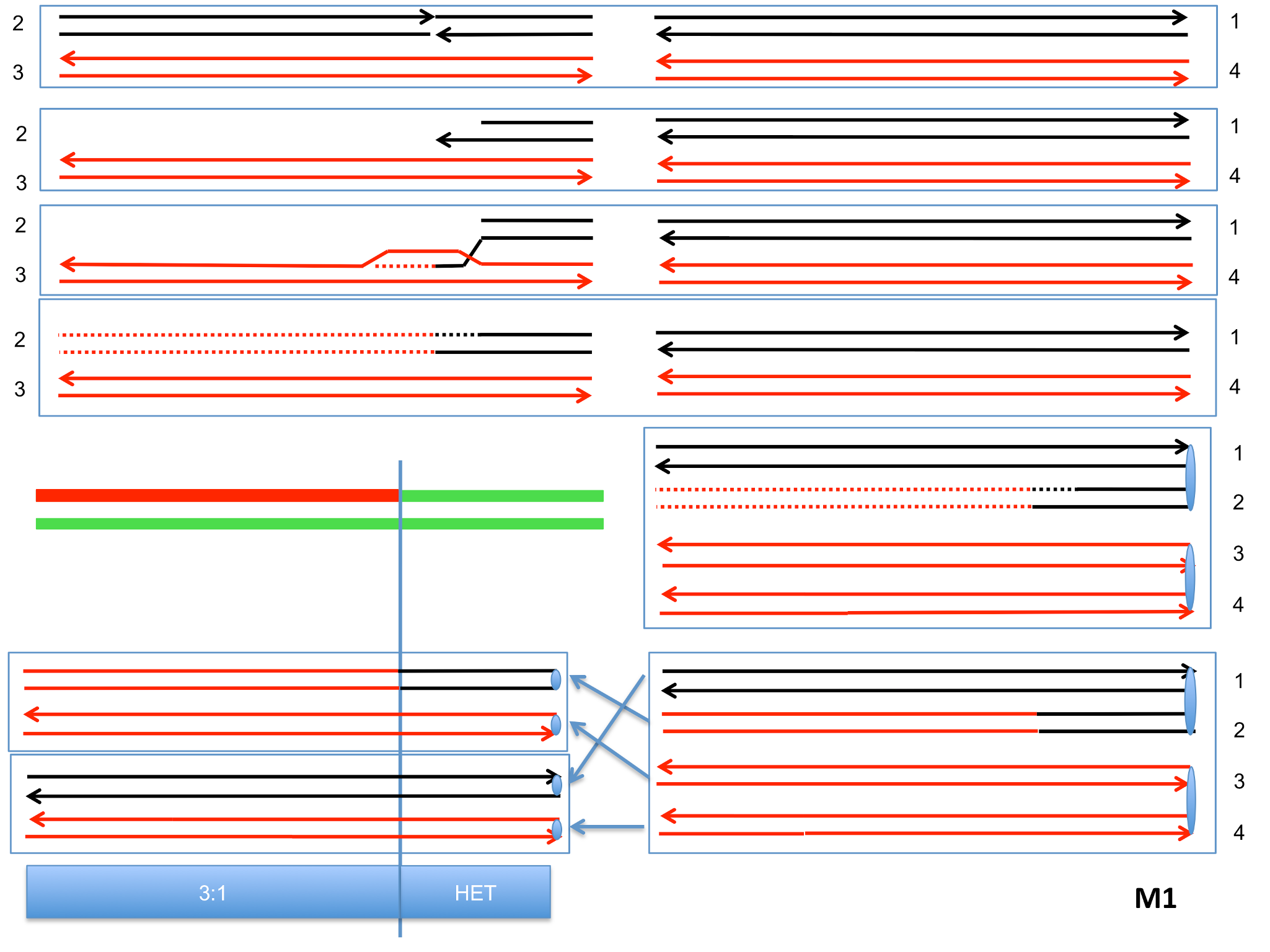

Supplement: Figure S14 — Generation of Class M1 by BIR. One broken black chromatid initiates a BIR event that copies a red chromatid to the end of the chromosome. (TIF) [file pgen.1003894.s014.tif]

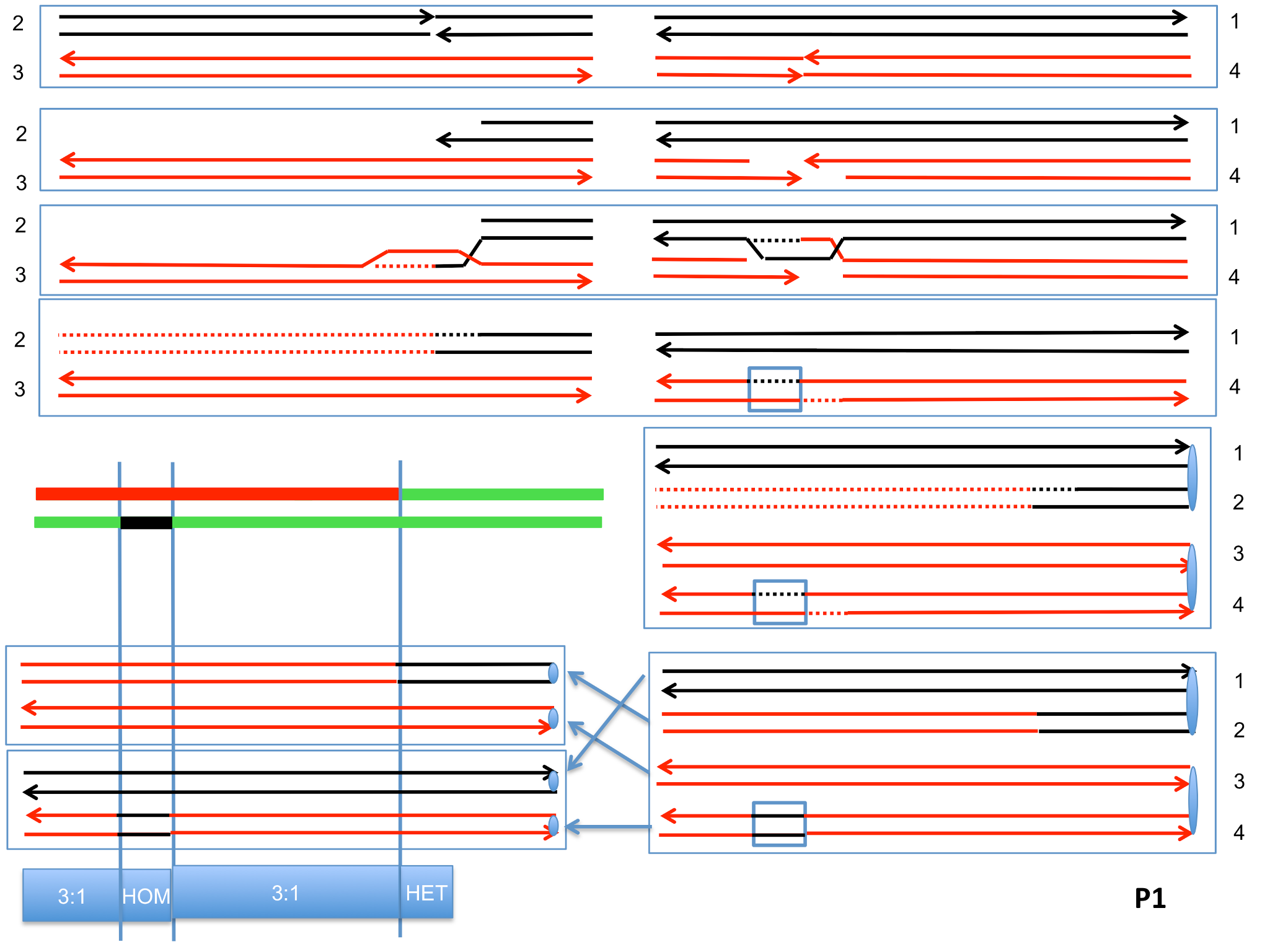

Supplement: Figure S15 — Generation of Class P1 by two independent repair events. One of the broken chromatids is repaired by SDSA and the second is repaired by a BIR event. (TIF) [file pgen.1003894.s015.tif]
