# Supplementary material for: Genome-Wide High-Resolution Mapping of UV-Induced Mitotic Recombination Events in Saccharomyces cerevisiae
Source: PLoS Genet. 2013 Oct 31;9(10):e1003894. doi: 10.1371/journal.pgen.1003894 (PMC3814309; doi:10.1371/journal.pgen.1003894)
Supplement: Text S1 — Supplemental materials and methods, and discussion. (DOC) [file pgen.1003894.s026.doc]

Text S1:

**Genome-Wide High-Resolution Mapping of UV-Induced Mitotic Recombination Events in *Saccharomyces cerevisiae***

Yi Yin and Thomas D. Petes

**Supplemental Materials and Methods**

***Yeast strains***

The diploid strain PG311 is a hybrid derived by crosses of haploids isogenic with W303a and YJM798 [1]. PG311 is heterozygous for all SNPs that distinguish W303a and YJM789 except for a small region (SGD coordinates 412715-414085) on chromosome X that is homozygous for SNPs derived from W303a and the region centromere-distal to SGD coordinate 685 kb on chromosome XIII, also homozygous for W303a-derived SNPs [2]. The genotype for PG311 is: *MAT***a***/ MATα::NAT ade2-1/ade2-1 can1-100/can1∆*::*SUP4-o ura3-1/URA3 trp1-1/TRP1 his3-11,15/HIS3 leu2-3,112/LEU2 V9229::HYG/V9229 V261553::LEU2/V261553 GAL2/gal2 RAD5/RAD5*. Since PG311 has *MAT****a***, but lacks *MATα* information, the strain is incapable of undergoing meiosis and can be synchronized in G1 using *α*-factor, as described below. We also examined the frequency of UV-induced mitotic crossovers in PSL101, the progenitor diploid of PG311. This strain is identical to PG311 except that it has both *MAT***a** and *MATα* information, and lacks the small region of LOH on chromosome X described above.

As a control for the SNP microarray analysis, we used genomic DNA isolated from the diploid strain JSC24-2 that is very similar to PG311 but does not have LOH regions on chromosomes X and XIII. JSC24-2 was constructed by mating haploids PSL2 (isogenic with W303a) and PSL5 (isogenic with YJM789), the same strains used in the construction of PG311 [1], to generate JSC23. In JSC23, the *MATα* gene was replaced with *NAT* using the same procedure as performed with PG311 [1] to generate JSC24-2. The related diploid strain JSC25-1 [3] was used for the experiments in which G2-synchronized cells were micromanipulated as single cells to specific positions on solid medium. With this strain, mitotic crossovers on the right arm of chromosome IV can be identified. The genotype for JSC25-1 is: *MAT****a****/MATα::HYG ade2-1/ade2-1 can1-100∆::NAT/ CAN1∆::NAT ura3-1 /ura3 trp1-1/TRP1 his3-11,15/HIS3 leu2-3,112/LEU2 RAD5/RAD5 IV1510386::KANMX-can1-100/IVI1510386::SUP4-o GAL2/gal2*.

We examined crossovers within the ribosomal DNA (rDNA) and in flanking regions of chromosome XII in a diploid derivative of AMC45 [4]. This derivative (YYy13) is a *MATα::NAT* version of AMC45 in which *MATα *was deleted as described previously [1]; two independent transformants (YYy13.7 and YYy13.9) were used in our studies. AMC45 and YYy13 are isogenic with the haploid strain MS71 [5]. YYy13 has the genotype: *MAT****a****/ MATα::NAT* *ade5-1/ade5-1 leu2-3/leu2-3 trp1-289/trp1-289 ura3-52/ura3-52 his7-2/his7-2 XII451250::HYG/XII451250 XII460158::TRP1/XII460158 XII490694/XII490694::K. lactis URA3.* This strain is heterozygous for markers centromere-proximal to the rDNA (*HYG*), within the rDNA (*TRP1*), and centromere-distal to the rDNA (*URA3*). The *TRP1* gene is located about 940 kb from the centromere-proximal end of the rDNA and about 230 kb from the centromere-distal end [4].

To confirm that the linkage of markers in AMC45 was not altered in YYy13, we sporulated and dissected tetrads of YYy13. Since YYy13 lacks *MATα* information, sporulation medium contained 5 mM nicotinamide to allow expression of silent mating type information [2]. We confirmed the correct linkages of the *HYG*, *TRP1*, and *URA3* markers. Interestingly, the frequency of meiotic recombination within the rDNA was elevated in YYy13 relative to AMC45. The map distances in the rDNA were 10 cM in YYy13 and 1 cM in AMC45. This difference presumably reflects nicotinamide-induced unsilencing of recombination in the rDNA [6].

***Cell synchronization and UV treatment***

In the experiments in which PG311 cells were synchronized and plated directly, cells were arrested in G1 using *α*-factor and in G2 using nocodazole using the conditions described by Lee and Petes (2010) [7]. After two hours, the cells were plated on omission medium lacking arginine, and immediately treated with UV (254 nm) using a TL-2000 UV Translinker at doses varying between 1 and 15 J/m^2^. The plates were then covered with foil and incubated for two days. For several experiments involving the 10 and 15 J/m^2^ treatment of G1-synchronized cells, we used two minor modifications of the procedure. About 20% of the sectored colonies were isolated using omission medium lacking arginine and containing canavanine; on such plates, PG311 cells that have not undergone a recombination event on chromosome V fail to grow [1]. The second modification is that about half of the cells formed colonies at room temperature and the other half were incubated at 30^0^ C. Although neither of these variables resulted in significant differences in frequency of recombination events, we indicate the conditions used to obtain each sectored colony in Table S1. For all experiments involving the “single-cell” procedure (described below), G2-synchronized cells, or 1 J/m^2^ UV treatment, the treated cells were plated on omission medium lacking arginine, and allowed to form colonies at 30^0^C.

For some experiments, PG311 and PSL101 strains were synchronized in G1 using a different approach. The cells were grown in liquid YPD until they reached stationary phase [8].The cells were transferred into fresh YPD for one hour, and then plated for irradiation as described above; >90% of the cells were unbudded at the time of UV treatment.

In the “single-cell” experiments, cells were arrested in G1 using *α*-factor or in G2 using nocodazole. Cells were incubated with the synchronizing agents for periods varying from 1 hour and 40 minutes to two hours. The treated cells were then transferred to a small area of a plate containing omission medium lacking arginine and single cells were micromanipulated to defined positions on the plate using a tetrad dissection apparatus. For each experiment, about 90 cells were moved in a 20-minute time period. The cells were then irradiated with 15 J/m^2^ of UV.

We also did a “single-cell’ experiment using unsynchronized cells. For this experiment, exponentially-growing cells were used (O.D. _660_=0.2-0.4). We transferred single cells in which the bud was approximately the same size as the daughter to defined positions on the plate; such cells should be in G2 of the cell cycle. These cells were treated with UV and incubated at 30^0^C. to allow colony formation.

For most experiments, cells were purified from each sector of red/white sectored colonies. As described in the text, we also analyzed some colonies with red, white, and pink sectors. Genomic DNA was isolated from each of these strains by standard techniques and examined by SNP microarrays.

***Analysis of UV-induced and spontaneous mitotic recombination events in YYy13.***

In the experiments for monitoring UV-induced recombination in the rDNA cluster, we arrested cells in G1 for two hours with *α*–factor, and then plated 50-200 cells on omission medium lacking arginine. The cells were then treated with 15 J/m^2^ of UV. After incubation at 30°C for two days, colonies were replica-plated onto YPD+ hygromycin and omission media lacking either uracil or tryptophan. Colonies that formed sectors on any of these three types of plates were purified. The purified single colonies derived from these sectors were re-checked on the same three types of plates. In addition, we replica-plated patches of cells derived from Ura^+^ sectors to medium containing 5-fluroro-orotate [4]. The colonies from some Ura^+^ sectors produced 5-FOA^R^ papillae at a frequency of 10^-6^, whereas other Ura^+^ sectors fail to produce such papillae. As described in the Results section, the ability or inability to form 5-FOA-resistant papillae indicates that the strain is heterozygous or homozygous, respectively, for the *URA3* gene. On the basis of these tests, sectored colonies of seven different phenotypic classes were observed. These events are described in detail in the Results section.

**Supplemental Discussion**

**Analysis of sectored colonies derived from “single-cell” experiments.**

As described in the text, in some experiments, we irradiated G1-synchronized cells and micromanipulated the irradiated cells to specific locations on plates. Thus, sectored colonies generated from this procedure were derived from single unbudded cells. In examining about 1000 colonies, we found seven red/white sectors, two pink/red sectors, and two pink/white/red sectors. We purified single colonies from each sector, isolated DNA from cells derived from these colonies, and examined the patterns of LOH using SNP microarrays. The results of this analysis are summarized in Figure S1.

All seven red/white sectored colonies had reciprocal LOH events on chromosome V consistent with a crossover (Figure S1A). In both pink/red sectors, the pink sectors had a small region of LOH on chromosome V, whereas the red sector was monosomic for chromosome V (Figure S1B), retaining the W303a-derived chromosome and losing the *SUP4-o-*containing YJM789-derived chromosome. In one of the pink/white/red sectors (Figure S1C), the white and red sectors had reciprocal patterns of LOH and the pink sector was monosomic for a recombinant chromosome and shared the LOH patterns observed for all of the chromosomes in the white sector. The simplest explanation of this pattern is that there was a reciprocal crossover on chromosome V during the first division and a subsequent loss of a chromosome from the “white” daughter cell. The pink/red sectored colonies 407PR and 409PR were examined for unselected LOH events on other chromosomes. Since we do not know whether chromosome V was lost in the first division or in a later division, we did not characterize the recombination events in 409PR as SCBs or DSCBs.

We found one pink/white/red sectored colony (78PWR) with patterns of LOH consistent with persistence of recombinogenic lesions into the second cell cycle (Figure S1D). The pink sector was heterozygous for all SNPs on chromosome V, and the red and white sectors had reciprocal patterns of LOH. Since this result indicates that a crossover was induced in one of the daughter cells, recombinogenic DNA damage persisted into the second cell cycle. We also examined the three sectors of 78PWR for unselected markers on other chromosomes. These markers showed the pattern expected if the DNA damage was recombinogenic in the first cell cycle. One example of a conversion event on chromosome II is shown in Figure S2. The pink sector had a larger conversion tract than the tracts of the white or red sectors that were identical (Tables S4 and S5). These results suggest that most recombination events are induced in the first division following UV treatment. In addition, since unrepaired mismatches within a heteroduplex would lead to different patterns of LOH in the white and red sectors, these results are consistent with the efficient repair of DNA mismatches (the absence of post-mitotic segregation).

**Analysis of UV-induced recombination events in the ribosomal DNA and in intervals flanking the rDNA**

As described in the main text, analysis of recombination events involving the ribosomal DNA cluster was done in the diploid YYy13, a diploid with heterozygous markers located centromere-proximal to the cluster (*HYG*), within the cluster (*TRP1*), and centromere-distal to the cluster (*URA3*) (Figure 7, Figures S3 and S4). YYy13 cells synchronized in G1 were treated with UV at a dose of 15 J/m^2^ and plated non-selectively. The resulting colonies were replica-plated to medium lacking uracil or lacking tryptophan or containing hygromycin. As described in the main text, crossovers occurring between the homologs in the *CEN12-HYG*, *HYG-TRP1*, and *TRP1-URA3* intervals could be identified unambiguously by their patterns of marker segregation within the sectors. In addition to crossovers (Figure 7), we also found UV-induced BIR events (Figure S3) and intrachromatid recombination events (Figure S4).

In Figure S3, we illustrate six different BIR events. Figures S3A and S3B show BIR events initiated by DSBs located between the *CEN12* and the *HYG* marker on the *HYG*-containing chromosome (Figure S3A) and the *URA3*-containing homolog (Figure S3B). Similarly, Figures S3C and S3D show BIR events initiated by DSBs located between the *HYG* and the *TRP1* markers on the *HYG*-containing chromosome (Figure S3C) and the *URA3*-containing homolog (Figure S3D). Lastly, Figures S3E and S3F show BIR events initiated by DSBs located between the *TRP1* and the *URA3* markers on the *HYG*-containing chromosome (S3E) and the *URA3*-containing homolog (S3F). The Ura^+^ sectors observed with crossovers (Figure 7) are homozygous for the wild-type URA3 gene and, therefore, do not form 5-FOA^R^ papillae on medium containing 5-FOA. In contrast, some of the Ura^+^ sectors associated with BIR will form 5-FOA^R^ papillae, indicated as 5-FOA^Paps^ in Figures S3 and S4. Unlike the patterns of marker segregation observed in the crossover events, these patterns do not uniquely distinguish among all possible BIR events. Although the BIR events shown in Figures S3A, S3C, and S3E are unique, the BIR events shown in Figures S3B. S3D, and S3F result in identical sector phenotypes. The latter three classes of BIR events, however, can be distinguished by dissecting the Ura^-^ sectors of the diploids. Dissection of the Ura^-^ sector shown in Figure S3B will result in four Hyg^R^ Trp^+^ Ura^-^ spores. A BIR event in which the position of invasion is between the *HYG* and *TRP1* genes (Figure S3D) will result in two spores that are Hyg^R^ Trp^+^ Ura^-^ and two spores that are Hyg^S^ Trp^+^ Ura^-^. Lastly, a BIR event that initiates between the *TRP1* and *URA3* genes (Figure S3F) will produce Ura^-^ diploids that, when sporulated, result in two Hyg^R^ Trp^+^ Ura^-^ spores and two Hyg^S^ Trp^-^ Ura^-^ spores. Finally, it should be noted that one type of BIR event (Figure S3E) does not result in a colony that is sectored for any of the markers and will not be detectable in our studies. The number and types of BIR events are summarized in Table 1.

A sub-set of intrachromosomal recombination events can also be distinguished in YYy13 as events in which the colony has Trp^+^/Trp^-^ sectors but remains heterozygous for the *HYG* and *URA3* markers (Figure S4). Loss of the *TRP1* marker can occur by a single-stranded annealing reaction (Figure S4A), a “pop-out” of the *TRP1* gene by an intrachromatid crossover, or an unequal sister-chromatid crossover in which the exchange occurs between misaligned *TRP1* insertions (Figure S4B). On the basis of past studies [9-11], it is likely that both unequal sister-chromatid crossovers and single-strand annealing events produce sectored Trp^+^/Trp^-^ colonies.

**Associations of gene conversion tracts with elements of chromosome structure and sequence**

The conversion tracts observed in our study should contain the initiating site of the recombinogenic DNA lesions. Consequently, we examined conversion tracts to determine whether they were enriched for various elements of chromosome structure or sequence. This analysis, similar to that performed previously for spontaneous recombination events on chromosome IV [3], involves several steps. The primary dataset used for the analysis are the unselected gene conversion and crossover events depicted in Tables S2 and S3. Events on the left arm chromosome V were excluded. Most (377 of 410) tracts used in the analysis were derived from red/white sectored colonies in which the conversion tracts were derived from a single site. However, we included 18 events in which there were two initiating sites within one chromosome arm (boxed in Table S2), and 15 unselected conversion events from the pink/red sectored colony 409PR (Table S5). In summary, for the analysis of unselected conversion events of all types, we analyzed 410 conversion events derived from 48 sectored colonies.

For each of these conversion/crossover events, we defined a “window” consisting of the sequences from the first transition of the leftmost marker defining the tract to the last transition of the rightmost marker. For example, for the conversion event shown in Class C1 in Table S2, the transitions used in the analysis are marked by the letters “a” and “c”. In sectored colony 8RW, the leftmost transition point is 985827 and the rightmost point is 992929. Since the SGD coordinates that were used for constructing the microarrays and the transition points shown in Table S2 were based on the SGD values of 2009, we obtained the translation of the 2009 coordinates to the 2011 coordinates from SGD (<http://www.yeastgeome.org/>); this correction usually involved an adjustment of 20 bp or less, except on chromosome XI in which the adjustment was about 300 bp. The conversion window for the Class C1 event of 8RW was about 7 kb. There were eight unselected crossovers that had no detectable associated conversion tracts. For these events, we used the window between the closest heterozygous and homozygous SNPs. In a similar manner, we determined the conversion/crossover windows for all 410 events. We then determined the number of individual chromosome structure/sequence elements listed in Tables S9 and S10 within these windows, and used chi-square analysis to determine whether there was an over-representation of these elements based on the number of the elements in the genome. An example of this analysis is discussed below using the tRNA genes as a representative chromosome element.

We found 66 tRNA genes within the 410 conversion tracts in 48 sectored colonies. The sum of the lengths of the conversion tracts was 4.1 Mb. The expected number of tRNA genes within the conversion tracts was calculated in multiple steps. First, we determined the number of tRNA genes in the genomic region of PG311 represented by our SNP microarray. The regions of each chromosome that were examined in our microarray analysis are shown in Table S8. We excluded from our analysis the left arm of chromosome V (the selected region), the sub-telomeric regions, and a previously-defined region on the right arm of XIII that is homozygous for W303a-derived SNPs [2]. The region examined contains about 90% of the total genome. The number of tRNA genes in the examined region (based on SGD) of the genome is 264. In the genomes of 48 strains, therefore, the total number of tRNA genes is 12672. The sum of all examined sequences among the 48 strains was 538 Mb. Thus, the expected number of tRNA genes within conversion tracts if the distribution is random is: 12672 x (4.1 Mb/538 Mb) = 96. The expected number of tRNA genes not involved in conversion tracts is: 12672 – 96 = 12576. We compared the observed number of tRNA sequences located within and outside of the conversion tracts (66 and 12606) with these expected numbers using “chisq.test” function in Excel. The uncorrected p value for this comparison was 0.002, which remains significant following correction for multiple comparisons [12].

Table S9 shows similar calculations applied to other elements of chromosome structure/sequence described in SGD including non-coding RNAs (ncRNA), small nucleolar and nuclear RNAs (snoRNA and snRNA, respectively), retrotransposons (primarily Ty1 and Ty2), centromere sequences, ARS elements, and solo LTRs (primarily delta elements). ARS elements were classified as early/middle ARS and late ARS according to the data of Fachinetti *et al.* (2010) [13]. Other than the tRNA genes, the only class of element that was significantly under-represented within the conversion tracts was solo LTR genes. For purposes of this analysis, the solo LTRs were defined as those that were >1 kb away from a Ty element. Since retrotransposons have a preference for integration near tRNA genes [14], we cannot determine whether the tRNA genes or the solo LTRs influence mitotic recombination. It is possible that neither element is causally related to the mitotic recombination phenotype.

The top third of Table S9 shows the analysis of all unselected conversion/crossover tracts. We also did a separate analysis of the unselected conversion tracts that had been classified as SCB (205 events) or DSCB (167 events) tracts. For this analysis, the whole tract was included from the rightmost to the leftmost transition. Since we did not include the data derived from the pink/red sectored colony (409PR), we had only 47 sectored colonies in the dataset instead of 48. The data analysis for SCB and DSCB tracts considered separately are also in Table S9. Of the events that could be unambiguously characterized about 55% were SCB and 45% were DSCB.

In addition to examining conversion tracts for over- or under-representation of chromosome elements annotated in SGD, we looked at these relationships for chromosome sequence/structure elements defined in other studies (Table S10) including: palindromes >16 bp [15], quadruplex DNA (four tracts of three Gs separated by less than 25 bp; [16]), highly- and weakly-transcribed genes [17], pause sites for the Rrm3p helicase [18], peaks of gamma-H2AX [19], replication termination (TER) sites [13], and regions with delayed repair of UV damage [20]. To determine the relationship between transcription and the conversion tracts, we used the dataset of Nagalakshmi *et al.* (2008) [17] to identify genes that ranked in the top or bottom 5% for transcription rates, and we determined whether these genes were over- or under-represented within the conversion windows. Pause sites for Rrm3p and peaks of gamma-H2AX activity were examined since it has been hypothesized that these regions represent chromosome fragile sites. TER regions have been associated with DNA polymerase pausing, and we previously showed that such sites were significantly associated with spontaneous mitotic conversion tracts [3]. We also examined regions of the genome in which the repair of UV damage was inefficient. Teng *et al.* (2011) [20] used physical methods to map regions of the yeast genome in which repair of dimers was not detectable two hours post-irradiation. We looked for associations between these regions and our conversion tracts.

As in Table S9, we separately analyzed all conversion/crossover tracts, tracts associated with SCB events, and tracts associated with DSCB events. After corrections of the p values for multiple comparisons, no significant associations with any of these elements were detected.

**Descriptions of conversion/crossover classes**

In Tables S2, S4, and S6, we depict various classes of UV-induced recombination events that were observed in colonies with a crossover on chromosome V. Most of these events can be explained as outcomes of the repair of one (SCB) or two (DSCB) broken DNA molecules by the pathways shown in Figure 1. To be classified as “simple” events, we require that mismatches within a heteroduplex are repaired to generate a conversion event, rather than restoration-type repair, and that repair of mismatches within a heteroduplex be non-patchy. In addition, we assume that the Holliday junctions formed during recombination do not branch migrate. As discussed below, our explanation of some of the recombination events (termed “complex”) invoke restoration-type repair, patchy repair and/or branch migration. Since many of the types of recombination events observed in the present study have been previously depicted [2], we will refer to these figures below. For patterns that have not been observed previously, we will show one explanatory supplementary figure for each class. Although some of the complex events can be explained by more than one series of events, we attempted to choose the model with the fewest deviations from the standard mechanisms shown in Figure 1.

We will first discuss the classes shown in Table S2. Classes A-E, H, I, and M are simple events (as defined above), and all other classes represent complex events. Class A events (3:1 conversions unassociated with a crossover) reflect the repair of one broken chromatid by the SDSA pathway, and are shown in Figure S1 in [2]. Class B events (4:0 conversions unassociated with a crossover) can be explained as the repair of two broken chromatids by the SDSA pathway in which the conversion tracts are of equal length (Figure S2 in [2]). Class C events, 3:1/4:0 hybrid conversion tracts unassociated with a crossover, can be explained as reflecting the repair of two DSBs by the SDSA pathway in which one repair tract is longer than the other (Figure S3 in [2]). Class D events (a 3:1 conversion tract in which the homozygous region is in different sectors) is explicable as a consequence of the repair of two DSBs by the SDSA pathway in which the conversion tracts are propagated in different directions (Figure S4 in [2]). Class E events are 3:1/4:0/3:1 conversion tracts unassociated with crossovers of two different types. In Classes E1 and E2, the homozygous regions of the 3:1 tracts are in different sectors, and in Classes E3 and E4, these regions are in the same sector. Classes E1 and E2 can be explained as a consequence of the repair of two DSBs, one by the SDSA pathway and one by the canonical DSBR pathway (Figure S5). Classes E3 and E4 also involve the repair of two DSBs, one by the SDSA pathway and one by repair of mismatches in heteroduplexes in a double HJ intermediate, followed by the dissolution of the recombined strands (Figure S6).

Class E events could also result from two independent, but closely associated, breaks on sister chromatids (Figure S7). If gene conversion tracts were propagated unidirectionally from these breaks, four different patterns of repair are expected. One-quarter of the events would be expected to have 3:1 tracts interrupted by a heterozygous region (Figure S7D). We observed 121 conversion tracts unassociated with crossovers with a 4:0 region, but only one sectored colony with two closely-linked 3:1 tracts. We conclude, therefore, that most 4:0 and hybrid 3:1/4:0 tracts are a consequence of breaks located at the same position on two sister chromatids.

Class F events are complex, and involve either restoration-type repair for whole repair tracts (F1 and F2) or patchy repair within one conversion tract (F3-F10). An example of the F1 class is shown in Figure S8, and an example of the F3 class is shown in Figure S6 of [2]. In Figure S8, the term “HOM” refers to an interstitial region of reciprocal homozygosity in the two sectors. Such regions can be generated by restoration-type repair of a double Holliday junction intermediate that is resolved symmetrically to generate a non-crossover product (Figure 1B). The Class G events are also complex and involve conversions with two different donors within one sectored colony. For example, in Class G1, a conversion tract in which information from the W303a-derived homolog is copied adjacent to a conversion tract in which information from the YJM789-derived homolog is copied. This type of event can be explained as a consequence of repair of a single DSB by SDSA in which mismatches in the heteroduplex formed during the strand invasion step are repaired prior to strand dissociation; following dissociation of the invading strand, the other two heteroduplexes are repaired, one by conversion-type repair and the other by restoration-type repair (Figure S9). Alternatively, Class G1 could be a consequence of repair of a single DSB resulting in a double HJ that branch migrates to produce a symmetric heteroduplex. Patchy repair within the resulting heteroduplex could produce the observed pattern (Figure S10). Similar mechanisms have been proposed previously (Figure S10 in [2]).

In the remaining classes, repair of the DSBs are associated with crossover (Classes H-L) or BIR events (Classes K-P). As for the previous events, mechanisms to explain many of the patterns conversion and crossovers were shown previously [2]. Class H events are crossovers without an observed conversion tract (H1 and H2; Figure S13 in [2]) or with 3:1 tracts (H3-H6; Figure S14 in [2]). Class H3-H6 events are assumed to reflect SCBs. In contrast, most Class I events (Classes I1-I10 and I13-I19) have 4:0 or 3:1/4:0 hybrid tracts associated with crossovers and are assumed to reflect DSCBs. The figures associated with the various Class I events in [2] are: Figure S16 (Classes I1-I3), Figure S17 (Classes I7-I10), Figure S18 (Classes I4-I6), Figure S19 (Classes I13-I15), Figure S20 (Classes I16-I19). In Classes I11 and I12, no 4:0 conversion event is observed. The simplest explanation of the “split” pattern for the 3:1 conversion tract, however, can be most simply explained as a consequence of DSCB repair (Figure S21 in [2]).

Class J events represent complex conversions associated with crossovers. Classes J1-J4 appear to be simple 3:1 conversion events associated with a crossover. However, unlike the 3: 1 events of Classes H3-H6 of Table S2, the homozygous portion of the conversion tract is in the wrong sector to reflect repair of a single SCB. More specifically, if a single DSB occurs on the red chromatid, the homozygous portion of 3:1 tract should be located in the sector that has the terminal red homozygous region associated with the crossover (Figure S14 of [2]). 3:1 conversion events in which the homozygous region is in the wrong sector can be explained as a consequence of the repair of a DSCB [1,2]. An example of the mechanism is shown in Figure S11. In this figure, we show two short regions of heteroduplex that we hypothesize are not detected because they fall between the diagnostic SNPs. Classes J5-J8 can be explained by repair of DSCBs in which one of the heteroduplex tracts associated with the crossover undergoes restoration-type repair; one example of this mechanism is shown in Figure S12. Classes J9-J16 have conversion tracts with multiple transitions between heterozygous and homozygous SNPs. These types of events can be explained as a consequence of patchy repair of mismatches within a heteroduplex and have been observed previously [2]. For example, Class J11 of the present study has the identical pattern to Class J2 by St. Charles *et al.*, (2012); the proposed mechanism of this event is depicted in Figure S23 of [2].

Class K events are similar to Class G (conversions with donors from both homologs within one sectored colony) expect that these events are associated with a crossover. As for the Class G, these patterns can be explained by allowing both conversion-type repair and restoration-type repair in different heteroduplexes or by patchy repair within a symmetric heteroduplex.

We interpret Class L as representing two independent recombinogenic lesions on one chromosome arm. If adjacent transitions were >15 kb, we considered the events independent; the distances that defined the average distance are shown in Table S2 for the Class L events. Classes L1-L11 involve independent conversions and crossovers, whereas Classes L12-L14, we interpret as double crossovers. One example of how double events can be produced the observed classes is shown for Class L1 in Figure S13. In this figure, we show a conversion event in which information is exchanged between the two homologs; a related type of conversion was shown in Figure 1B and Figure S8. Centromere-proximal to the conversion event, we show an independent conversion and crossover involving the same two chromatids. Following segregation of the recombined chromosomes, we obtain a Class L1 sectored colony (Figure S13B).

Class M events reflect BIR. In Classes M1-M4, there is a single non-reciprocal region of LOH that extends to the telomere. These classes are consistent with a SCB in which the broken fragment centromere-distal to the break is lost and the fragment proximal to the break invades one of the chromatids derived from the other homolog (Figure S14). Classes M5-M7 have the patterns of LOH expected for a DSCB in which two BIR events occur within a single cell and Class M8 has the pattern expected for repair of two broken chromatids, one by BIR and one by SDSA. Similarly, Class N1 is explicable as representing the repair of two broken chromatids, one by BIR and one by patchy repair of mismatches within a heteroduplex generated by SDSA. In Classes O1 and O2, there is a change in donors in the conversions associated with the BIR events. The change in donors can be explained by the mechanism shown in Figure S9. We hypothesize that Class P events, similar to Class L events, involve repair of two independent DSBs, one by gene conversion unassociated with a crossover and one by BIR (Figure S15).

In the last class, Class Q, one sector has undergone chromosome loss and the second sector has an interstitial region of LOH. Since only three of the four chromosomes participating in this event were recovered, we cannot determine whether the event reflects a SCB or a DSCB. No chromosome loss events were observed for any unselected chromosome.

As observed in the present study and our previous analysis of mitotic recombination events, most of the complex LOH patterns can be explained in more than one way. In the supplementary figures, we attempted to explain the events with the smallest number of modifications to the models shown in Figure 1. In addition, it is possible that some of the complex events may involve pathways of recombination inherently different from those shown in Figure 1. For example, the repair of DSBs could involve multiple cycles of end invasion, heteroduplex formation and mismatch repair, disassociation of the invading end, and re-invasion. This type of template switching has been demonstrated to occur during BIR [21]. Another possibility is that reciprocal LOH events could reflect double BIR events, following the aberrant processing of a Holliday junction [22]. Perhaps the most likely explanation of the complexity of patterns of mitotic recombination is that reciprocal recombination can utilize a wide variety of intermediates that can be processed in a wide variety of ways.

**References**

1. Lee PS, Greenwell PW, Dominska M, Gawel M, Hamilton M, et al. (2009) A fine-structure map of spontaneous mitotic crossovers in the yeast Saccharomyces cerevisiae. PLoS Genet 5: e1000410.

2. St Charles J, Hazkani-Covo E, Yin Y, Andersen SL, Dietrich FS, et al. (2012) High-resolution genome-wide analysis of irradiated (UV and gamma-rays) diploid yeast cells reveals a high frequency of genomic loss of heterozygosity (LOH) events. Genetics 190: 1267-1284.

3. St Charles J, Petes TD (2013) High-resolution mapping of spontaneous mitotic recombination hotspots on the 1.1 Mb arm of yeast chromosome IV. PLoS Genet 9: e1003434.

4. Casper AM, Mieczkowski PA, Gawel M, Petes TD (2008) Low levels of DNA polymerase alpha induce mitotic and meiotic instability in the ribosomal DNA gene cluster of Saccharomyces cerevisiae. PLoS Genet 4: e1000105.

5. Kokoska RJ, Stefanovic L, DeMai J, Petes TD (2000) Increased rates of genomic deletions generated by mutations in the yeast gene encoding DNA polymerase delta or by decreases in the cellular levels of DNA polymerase delta. Mol Cell Biol 20: 7490-7504.

6. Imai S, Armstrong CM, Kaeberlein M, Guarente L (2000) Transcriptional silencing and longevity protein Sir2 is an NAD-dependent histone deacetylase. Nature 403: 795-800.

7. Lee PS, Petes TD (2010) From the Cover: mitotic gene conversion events induced in G1-synchronized yeast cells by gamma rays are similar to spontaneous conversion events. Proc Natl Acad Sci U S A 107: 7383-7388.

8. Ma W, Resnick MA, Gordenin DA (2008) Apn1 and Apn2 endonucleases prevent accumulation of repair-associated DNA breaks in budding yeast as revealed by direct chromosomal analysis. Nucleic Acids Res 36: 1836-1846.

9. Gangloff S, Zou H, Rothstein R (1996) Gene conversion plays the major role in controlling the stability of large tandem repeats in yeast. EMBO J 15: 1715-1725.

10. Szostak JW, Wu R (1980) Unequal crossing over in the ribosomal DNA of Saccharomyces cerevisiae. Nature 284: 426-430.

11. Zamb TJ, Petes TD (1982) Analysis of the junction between ribosomal RNA genes and single-copy chromosomal sequences in the yeast Saccharomyces cerevisiae. Cell 28: 355-364.

12. Hochberg Y, Benjamini Y (1990) More powerful procedures for multiple significance testing. Stat Med 9: 811-818.

13. Fachinetti D, Bermejo R, Cocito A, Minardi S, Katou Y, et al. (2010) Replication termination at eukaryotic chromosomes is mediated by Top2 and occurs at genomic loci containing pausing elements. Mol Cell 39: 595-605.

14. Ji H, Moore DP, Blomberg MA, Braiterman LT, Voytas DF, et al. (1993) Hotspots for unselected Ty1 transposition events on yeast chromosome III are near tRNA genes and LTR sequences. Cell 73: 1007-1018.

15. Lisnic B, Svetec IK, Saric H, Nikolic I, Zgaga Z (2005) Palindrome content of the yeast Saccharomyces cerevisiae genome. Curr Genet 47: 289-297.

16. Capra JA, Paeschke K, Singh M, Zakian VA (2010) G-quadruplex DNA sequences are evolutionarily conserved and associated with distinct genomic features in Saccharomyces cerevisiae. PLoS Comput Biol 6: e1000861.

17. Nagalakshmi U, Wang Z, Waern K, Shou C, Raha D, et al. (2008) The transcriptional landscape of the yeast genome defined by RNA sequencing. Science 320: 1344-1349.

18. Azvolinsky A, Giresi PG, Lieb JD, Zakian VA (2009) Highly transcribed RNA polymerase II genes are impediments to replication fork progression in Saccharomyces cerevisiae. Mol Cell 34: 722-734.

19. Szilard RK, Jacques PE, Laramee L, Cheng B, Galicia S, et al. (2010) Systematic identification of fragile sites via genome-wide location analysis of gamma-H2AX. Nat Struct Mol Biol 17: 299-305.

20. Teng Y, Bennett M, Evans KE, Zhuang-Jackson H, Higgs A, et al. (2011) A novel method for the genome-wide high resolution analysis of DNA damage. Nucleic Acids Res 39: e10.

21. Smith CE, Llorente B, Symington LS (2007) Template switching during break-induced replication. Nature 447: 102-105.

22. Ho CK, Mazon G, Lam AF, Symington LS (2010) Mus81 and Yen1 promote reciprocal exchange during mitotic recombination to maintain genome integrity in budding yeast. Mol Cell 40: 988-1000.
